# Two Rhodium(III) Ions Confined in a [18]Porphyrin Frame: 5,10,15,20‐Tetraaryl‐21,23‐Dirhodaporphyrin

**DOI:** 10.1002/chem.202201513

**Published:** 2022-07-06

**Authors:** Grzegorz Vetter, Agata Białońska, Paulina Krzyszowska, Sebastian Koniarz, Ewa Pacholska‐Dudziak

**Affiliations:** ^1^ Faculty of Chemistry University of Wroclaw ul. Joliot-Curie 14 50-383 Wroclaw Poland

**Keywords:** fluxionality, metallacycles, porphyrinoids, rhodium, tellurium

## Abstract

Tetraaryl‐21,23‐dirhodaporphyrin and a series of related monorhodaporphyrins have been obtained by tellurium‐to‐rhodium exchange in a reaction of tetraaryl‐21,23‐ditelluraporphyrin with [RhCl(CO)_2_]_2_. These organometallic metallaporphyrins contain rhodium(III) centers embedded in rhodacyclopentadiene rings, incorporated within the porphyrin frames. The skeletons of 21,23‐dirhodaporphyrin and 21‐rhoda‐23‐telluraporphyrin are strongly deformed in‐plane from the rectangular shape typical for porphyrins, due to rhodium(III) coordination preferences, the large size of the two core atoms, and the porphyrin skeleton constrains. These two metallaporphyrins exhibit fluxional behavior, as studied by ^1^H NMR and DFT, involving the in‐plane motion and the switch of the rhodium center(s) between two nitrogen donors. A side product detected in the reaction mixture, 21‐oxa‐23‐rhodaporphyrin, results from tellurium‐to‐oxygen exchange, occurring in parallel to the tellurium‐to‐rhodium exchange. The reaction paths and mechanisms have been analyzed. The title 21,23‐dirhodaporphyrin contains a bridged bimetallic unit, Rh_2_Cl_2_, in the center of the macrocycle, with two rhodium(III) ions lying approximately in the plane of the porphyrinoid skeleton. The geometry of the implanted Rh_2_Cl_2_ unit is affected by macrocyclic constrains.

## Graphical abstract

**Pack more into the box**: How to fit two metal ions in one porphyrin. Porphyrins’ default coordination mode allows the binding of a single metal cation in the central NNNN cavity. In order to allow the accommodation of two metal ions inside a standard porphyrin perimeter, they are introduced *in place of two inner NH groups*, yielding a 21,23‐dimetallaporphyrin. The 18‐π‐electron aromatic circuit is maintained, playing a crucial role in macrocycle stabilization, and enforcing the metal centers’ proximity.

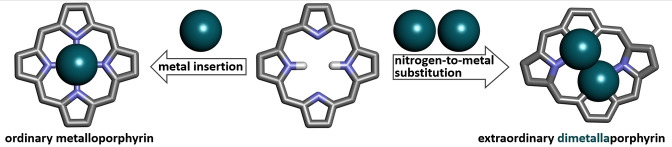

## Introduction

Porphyrin is one of the most widely studied macrocycles in coordination chemistry. The default binding mode in which the four porphyrin core donors serve as equatorial coordinators of a metal ion placed inside the cavity, within the N_4_ plane or slightly above, allows typically for a single central cation binding. Formation of multi‐cation porphyrin complexes is, however, possible by several means.

**Scheme 1:**
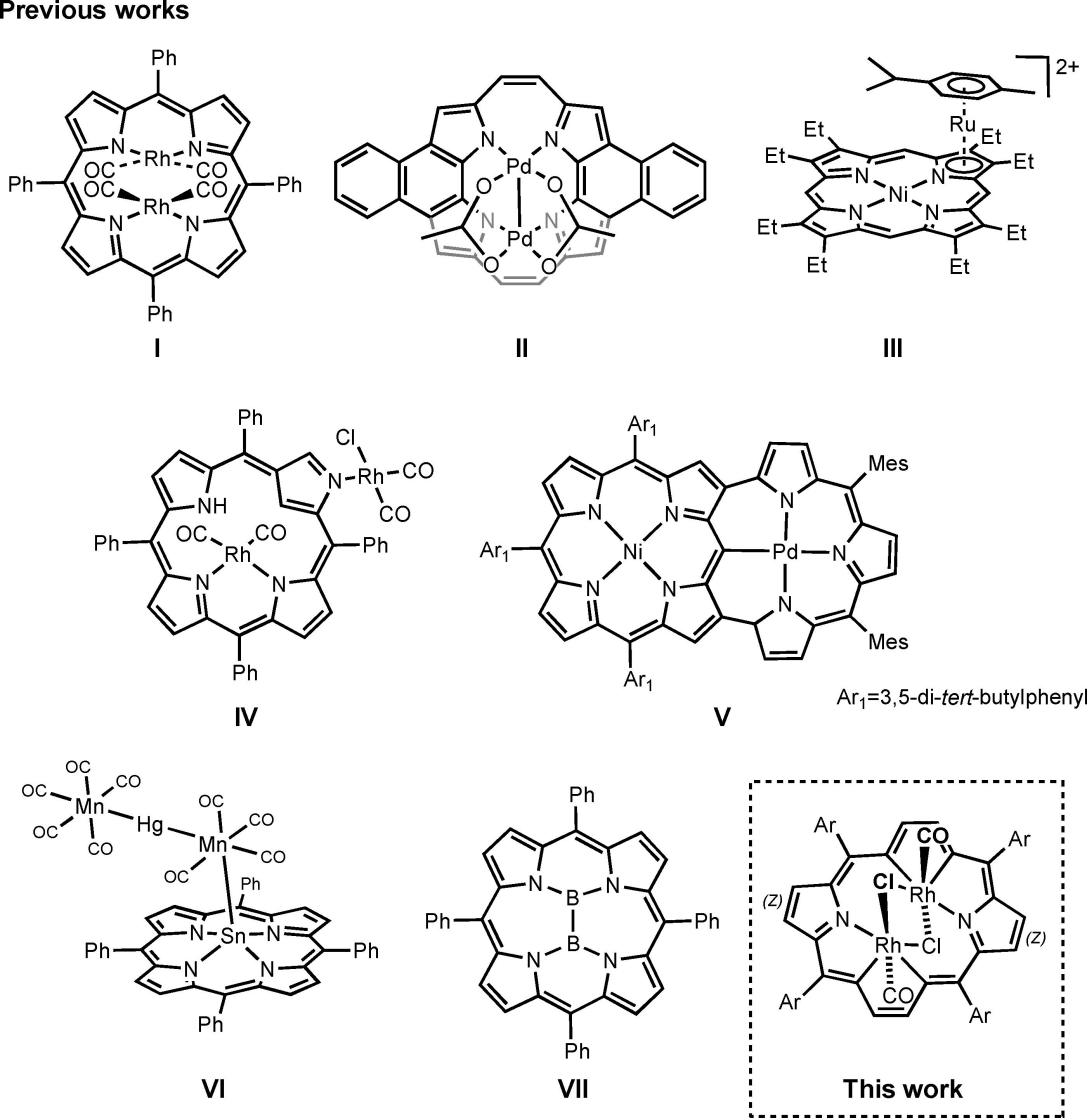
Chosen examples of bi‐ and polymetallic porphyrinoids.

The porphyrin can act as a bidentate ligand towards metal ions, allowing for formation of bis‐metallic complexes exemplified by a bis‐rhodium(I) complex (**I**, Scheme 1),^[1]^ with two metal centers located above and below the macrocyclic plane. A series of bimetallic porphyrin complexes of similar kind with two metal ions distant from the porphyrin plane, were reported for several low‐valent metals, such as rhenium(I), technetium(I), thallium(I) or gold(I).[^2^, ^3^, ^4^] The design of bis‐strapped porphyrins, equipped with additional donors hanging over each side of a porphyrin, allowed to encage two large metal cations, such as lead(II), bismuth(III), mercury(II) and thallium(I) on two sides of a porphyrin.^[5]^ An exceptional *cis*‐bimetallic complex was reported for a derivative of a [18]porphyrin isomer, dinaphthoporphycene, where both palladium ions sit above the macrocyclic core and exhibit significant metal–metal bonding interaction (**II**, Scheme 1).^[6]^

Another strategy of multiple metal ion binding is an employment of donors, other than core atoms, present within the porphyrin structure, that is, attached coordinating groups or integral components of the metalloporphyrin skeleton. Thus, in metal(II) complexes of azuliporphyrin, the azulene moiety serves as the π‐coordination platform to accommodate Ru_4_(CO)_9_ unit.^[7]^ In nickel(II)OEP complex one pyrrole ring serves as a π ligand for an arenophile, Ru(cymene) dication (**III**, Scheme 1).^[8]^ The peripheral nitrogen atom of N‐confused porphyrin binds rhodium ion(I) in a bis[rhodium(I)] complex (**IV**, Scheme 1).^[9]^ Such an N‐bound external rhodium(I) ion served also as bridge linking two inverted porphyrin units in an unprecedented gable porphyrin binding a trirhodium cluster.^[10]^

Another approach to build bimetallic organometallic complexes merges the standard N_4_ metal binding with peripheral carbon–metal σ‐bonding imported from pincer–ligand chemistry. Thus, *ortho*‐pyridyl substituents at β‐positions of neighboring pyrroles served as the pincer ligand for platinum(II) bound to the outer rim of nickel(II)porphyrin.^[11]^ Porphyrin–pincer hybrids evolved in subsequent studies into double‐ and triple‐cavity hybrid macrocycles, like “earring porphyrins” (**V**, Scheme 1).[^12^, ^13^] An entirely different type of a multi‐metal porphyrin derivative can be formed on the basis of σ metal–metal bonds extending over a metalloporphyrin ring,^[14]^ exemplified by a tin(II)porphyrin with attached Mn(CO)_4_−Hg−Mn(CO)_5_ unit (**VI**, Scheme 1).^[15]^

Insertion of two central ions into a porphyrin cavity is possible in case of boron, the only element small enough to fit in the standard [18]porphyrin core. Thus, a variety of bis‐boron structures, in both *cis* and *trans* arrangements, have been described for the porphyrin and related four‐pyrrole ligands.^[16]^ The enforced close proximity of two boron atoms stimulates unusual chemistry, like spontaneous reductive coupling, to give complexes with boron–boron bonds, like a rare example of a doubly reduced planar 20 π electron isophlorin complex (**VII**, Scheme 1).[^17^, ^18^]

Recently, an unconventional mode of a metal incorporation in a porphyrin, has been discovered, leading to formation of a 21‐*metallaporphyrin*, that is, a porphyrin with a metal atom located *in place* of NH group. Such a metalloporphyrinoid formally represents a 21‐heteroporphyrin, where a heteroatom replacing NH unit is chosen among metallic elements, and *metallacyclopentadiene ring* is embedded in place of a pyrrole ring. This class of stable, aromatic, organometallic macrocycles was limited to 21‐metalla‐23‐telluraporphyrins, containing one transition metal ion, palladium(II),^[19]^ platinum(II) or platinum(IV).^[20]^

## Results and Discussion

Herein, we present the synthesis of 21,23‐dirhodaporphyrin, the first 21,23‐dimetallaporphyrin, entrapping two transition metal cations in proximity, within the plane of a [18]porphyrin skeleton. Along with the bimetallic compound, we report a series of monometallic rhodaporphyrins.

### Synthesis and reaction paths

**Scheme 2:**
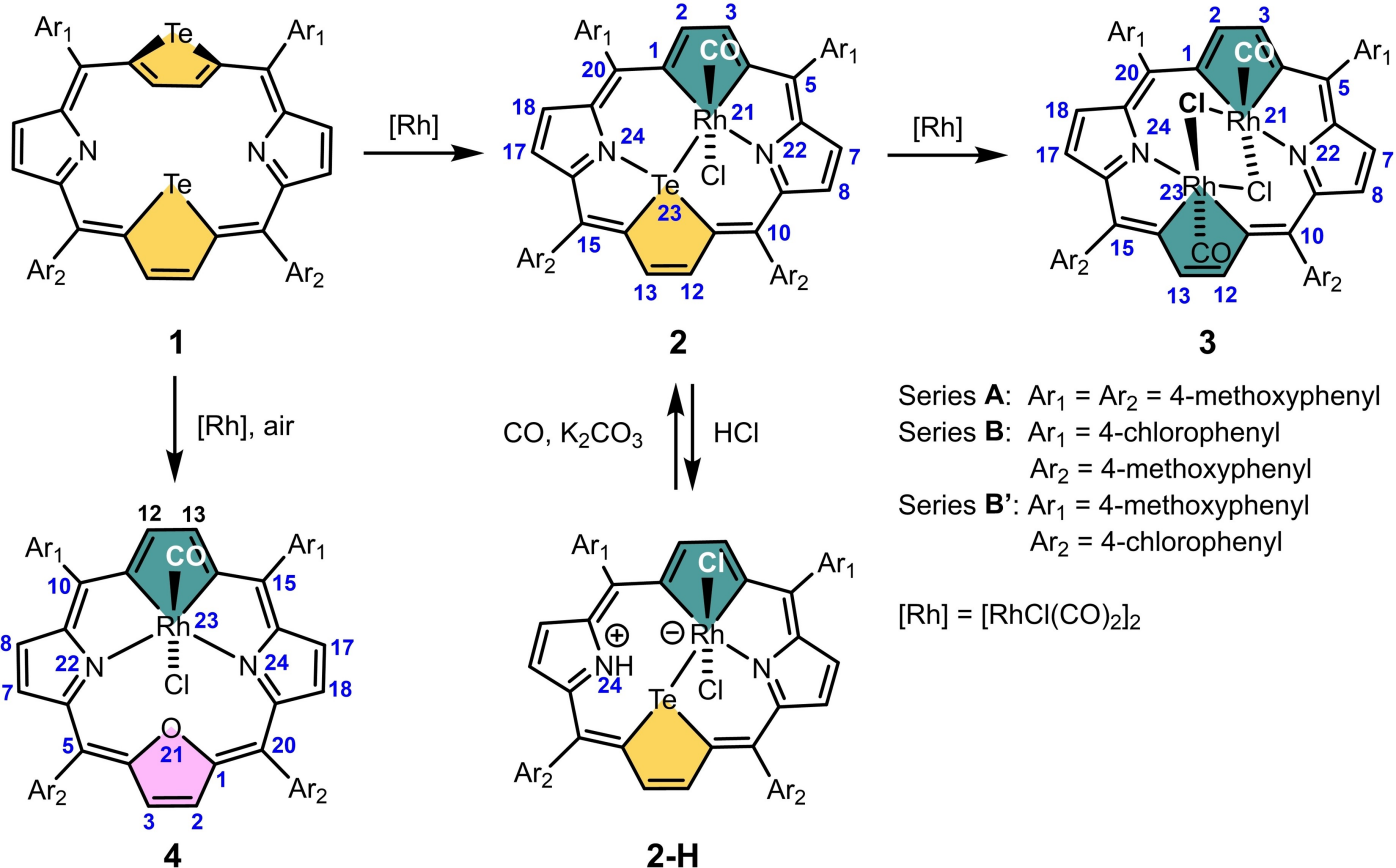
Synthetic procedures leading to rhodaporphyrins.

The synthetic strategy leading to rhodaporphyrins follows the tellurium‐replacement method developed in our previous works,[^19^, ^20^] where 21,23‐ditelluraporphyrin^[21]^ treated by metal(II) salts yielded 21‐metalla‐23‐telluraporphyrins. The choice of rhodium, among transition metals, is based on its documented potential to replace tellurium in tellurophenes to form rhodacyclopentadienes.^[22]^ The synthetic work is summarized in Scheme 2. The reactions were carried out for two series of *meso*‐tetraaryl derivatives (more in the Supporting Information), and these substituents will be omitted in the text for clarity. The spectroscopic, X‐ray and DFT data shown, were obtained for tetrakis(4‐methoxyphenyl) derivatives (series **A**), except for compound **5** (see below).

The reaction of 21,23‐ditelluraporphyrin, **1**, with [RhCl(CO)_2_]_2_ was carried out in boiling toluene, yielding a mixture of four different diamagnetic and aromatic 21‐rhodaporphyrins: **2**, **2**‐H, **3** and **4**, containing rhodium(III) centers. The common feature of all these compounds is the presence of rhodacyclopentadiene ring incorporated into the porphyrin skeleton, while the coordination environment of rhodium(III) and the mode of macrocycle deformation varies. The formation of 21‐rhoda‐23‐telluraporphyrin **2** required one heteroatom replacement, while the remaining tellurium atom served as a donor, completing the transition metal coordination sphere. Macrocycle **2** (11 to 30 % yield, depending on aryl substituents) with octahedral central ion coordinating two axial ligands: carbonyl and chloride anion, resembles known 21‐platina‐23‐telluraporphyrins containing platinum(IV).^[20]^

The macrocycle **2**‐H, closely related to **2**, was isolated from the reaction mixture in low yield (0.7–3 % yield), but can be easily obtained from **2** by treatment with hydrogen chloride. The transformation **2**→**2**‐H leads to protonation of a nitrogen atom accompanied by one axial ligand exchange, that is, neutral carbonyl to anionic chloride. The reaction is reversible upon addition of CO_(g)_ and K_2_CO_3_. Two other products, 21,23‐dirhodaporphyrin **3** (2.8–9 % yield) resulting from double tellurium to rhodium substitution, and the unscheduled product, 21‐oxa‐23‐rhodaporphyrin **4** (0–0.8 % yield), reveal new structural features. Tellurium to rhodium substitution in **2** leading to **3**, can be performed in boiling toluene with 9 % yield.

**Scheme 3:**
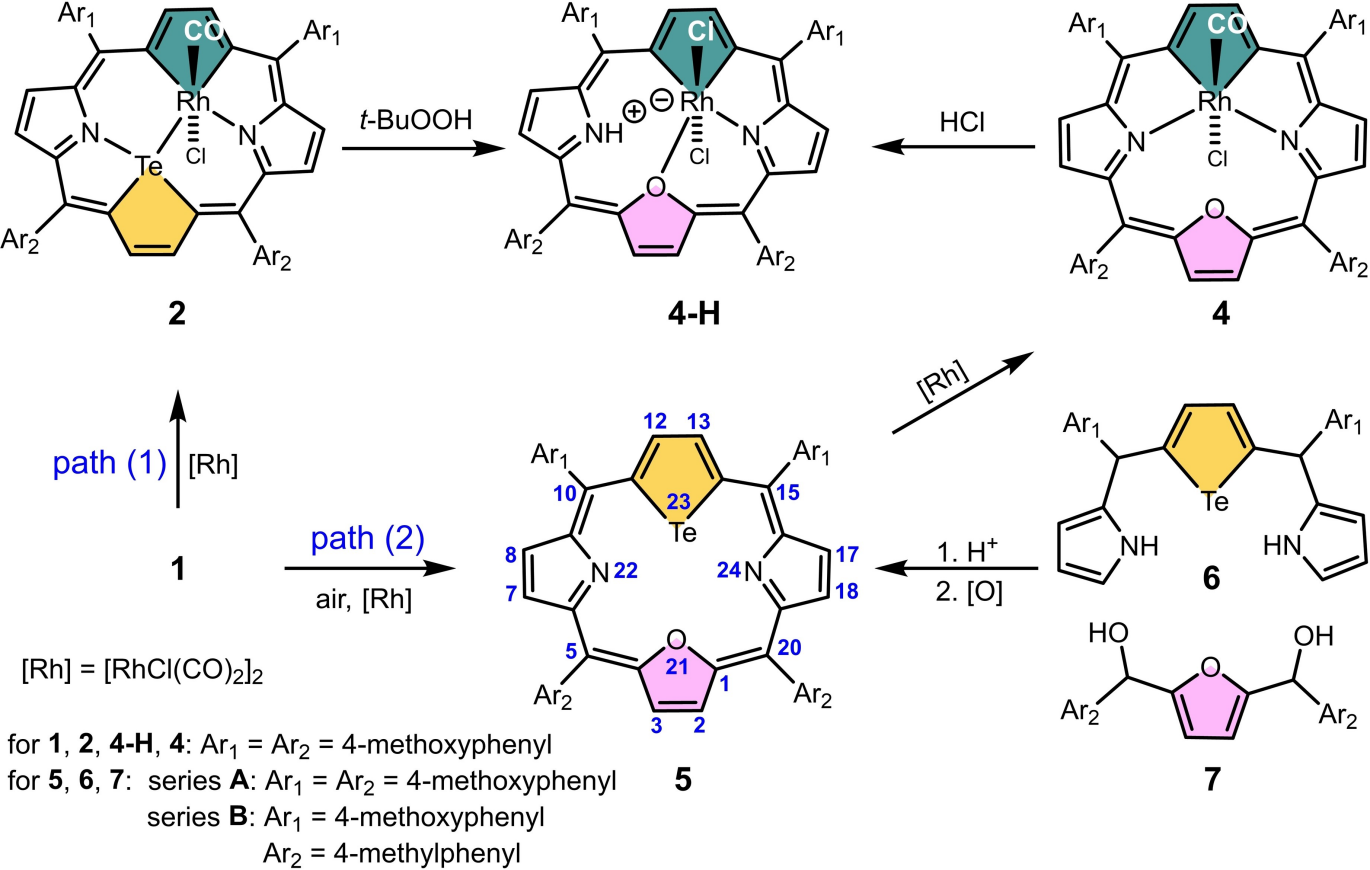
Paths leading to 21‐oxa‐23‐rhodaporphyrins **4** and **4**‐H.

Formation of 21‐oxa‐23‐rhodaporphyrin **4** from 21,23‐ditelluraporphyrin **1** arises from a double substitution: one tellurium to rhodium substitution is accompanied by tellurium to oxygen atom exchange, that is, tellurophene to furan ring transformation. Formation of this product has been avoided in strictly anaerobic conditions of a Schlenk line, leading to the conclusion that the oxygen atom built in the furan unit of **4** originates from the presence of oxygen in a typical reaction mixture. Similar reactivity of the tellurophene ring incorporated into an aromatic telluraporphyrin towards oxygen, has been reported for 21‐telluraporphyrin, which undergoes a light‐promoted oxidation in air to 21‐hydroxy‐21‐telluraporphyrin, and further transforms to 21‐oxaporphyrin with peroxides, H_2_O_2_ or MCPBA.[^23^, ^24^] In attempts to get more insight in the reaction path of **1** to **4** transformation, we considered two possible step sequences: 1) the oxygen introduction follows or 2) precedes the rhodium insertion (Scheme 3). If the first possibility was feasible, after initial conversion of **1** into **2**, compound **2** treated with oxygen should yield **4**. Although **2** proved fully air‐stable even at 110 °C, giving no confirmation for path (1), we found an oxidant, *tert*‐butyl hydroperoxide, capable of tellurium to oxygen substitution in **2**. The oxidation of **2**, however, did not yield **4**, but another 21‐oxa‐23‐rhodaporphyrin **4**‐H, a N‐protonated product related to **4** and a congener of **2**‐H. Alternatively, **4**‐H can be obtained by treating **4** with hydrogen chloride. Low yields of both paths leading to **4**‐H and its limited stability, impeded full characterization of this species.

If path (2) operates, **1** transforms to 21‐oxa‐23‐telluraporphyrin **5**, which subsequently undergoes transmetalation with rhodium, yielding **4**. Diheteroporphyrin **5**, incorporating two well known in heteroporphyrin chemistry building blocks, furan and tellurophene, was not present in the literature. We performed the synthesis of **5** (8.5 % yield), according to the well established [3+1] scheme^[25]^ from known precursors, **6** and **7**[^26^, ^27^] (Scheme 3). Macrocycle **5** proved susceptible to tellurium to rhodium substitution and gave the product **4** in satisfactory yield of 22 %. The possibility of a low‐yield transformation of 21,23‐ditelluraporphyrin **1** into 21‐oxa‐23‐telluraporphyrin **5** during the rhodium insertion, was confirmed by the presence of traces of **5** detected in the reaction mixture, which leads to the conclusion that path (2) of **1** to **4** transformation appears likely. The method of choice to obtain **4** in quantity allowing its characterization was based on the rational synthesis of **5** from **6** and **7**, followed by reaction with [RhCl(CO)_2_]_2_.

### Molecular structures

**Figure 1:**
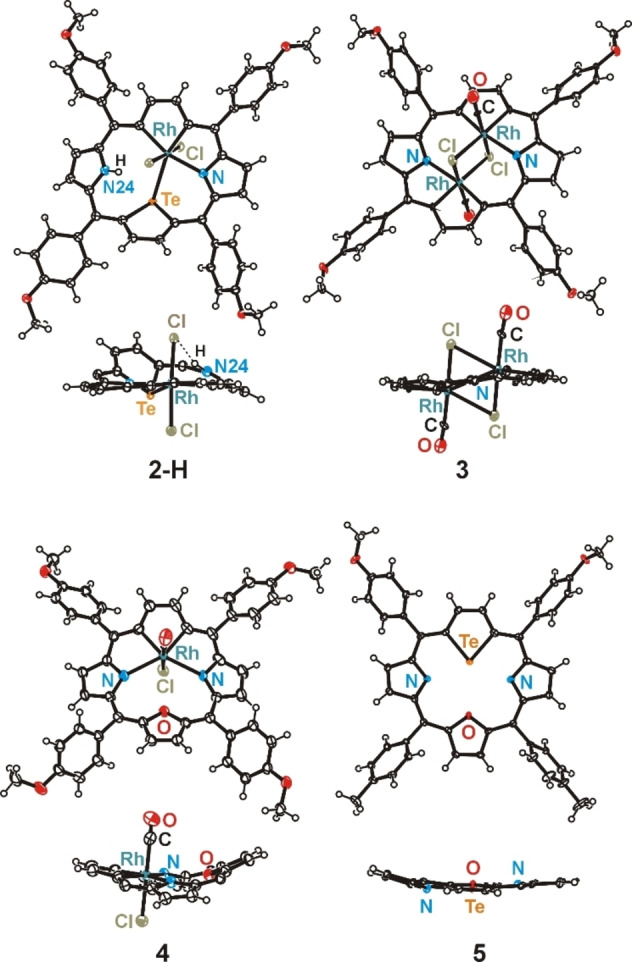
Molecular structures of **2**‐H, **3**, **4** and **5**. Displacement ellipsoids represent 50 % probability. In side views, the aryl rings are omitted for clarity.

Structures of rhodaporphyrins **2**, **2**‐H, **3** and **4** as well as new ligands **1**_(B)_ and **5**, were provided by X‐ray diffraction analysis (Figure 1 and Figures S39, S43, and S44 in the Supporting Information). In order to complement low quality structural data (structure **2**), which did not allow for detailed analysis of bond lengths and angles, and to support further discussion on reaction mechanism, we performed DFT calculations for studied coordination compounds. DFT‐optimized geometries are in accord with these found by X‐ray crystallography (Figures S39–S42, Tables S2 and S3).

**Figure 2:**
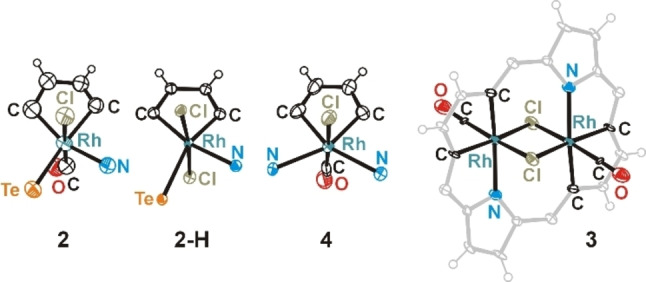
The coordination environment of rhodium(III) in rhodaporphyrins **2**, **2**‐H, **3** and **4**.

Presented molecular structures exhibit significant deformation of rhodaporphyrins skeletons, however their distortion mode varies. In molecules **2** and **2**‐H the presence of two large atoms inside the porphyrin core, tellurium and rhodium, in combination with coordination preferences of rhodium(III) ion, impose specific rhomboidal in‐plane deformation of the porphyrin skeleton. This structural feature is very characteristic for all known 21‐metalla‐23‐telluraporphyrins.[^19^, ^20^] The coordination geometry around rhodium(III) in **2** and **2**‐H is distorted octahedral, (NTeCC)ClC and (NTeCC)Cl_2_, respectively (Figure 2), and the bond lengths around the central ion are within ranges typical for rhodium(III) (Table S3).^[28]^ Substantial nonplanarity of **2** and **2**‐H is apparent as protrusion of tellurium atom from the macrocyclic plane and, in case of **2**‐H, as strong tilt of the protonated pyrrole ring. The tellurophene ring inclination reflects the propensity of organotellurium ligands for a side‐on coordination,^[28]^ and similar distortion has been previously reported for 21‐platina‐23‐telluraporphyrins.^[20]^ The nonplanarity of 21‐rhoda‐23‐telluraporphyrins is strongly influenced by nitrogen‐24 protonation (Figure S50). Whereas N24 in **2** is oriented towards tellurium atom and not much tilted, in **2**‐H the NH group is pointing to an axial chloride and this pyrrole ring is significantly leaned out of porphyrin C_4‐*meso*_ plane (24°). The NH hydrogen atom is situated at the hydrogen bond distance from axial chloride (N⋅⋅⋅Cl 3.264(12) Å), which is close to mean literature value (N⋅⋅⋅Cl 3.181(6) Å).^[29]^

The two‐fold symmetry of 21‐oxa‐23‐rhodaporphyrin **4**, with the mirror plane perpendicular to the C_4_‐*meso* plane and passing through rhodium and oxygen atoms is a new structural feature in the class of 21‐metalla‐23‐heteroporphyrins. Apparently, this molecule resembles the parent 21‐oxa‐23‐telluraporphyrin **5** (Figure 1) with regard to the skeleton symmetry, underlining the 21,23‐diheteroporphyrin nature of **4**, however the degree of the skeleton saddle deformation is substantial. The higher molecular symmetry (*C*_s_) of macrocycle **4** as compared to **2** (*C*_1_) is connected with a different bonding pattern within the core atoms, resulting from specific interatomic distances between available donor atoms and rhodium ion. In **4** the furan ring situated opposite to rhodacyclopentadiene unit exposes oxygen atom towards the rhodium(III) ion, but the Rh⋅⋅⋅O distance of 3.22 Å is much longer than any Rh−O bond (1.92–2.40 Å),^[28]^ shorter, however, than the sum of van der Waals radii (3.55–3.94 Å).[^30^, ^31^] The rhodium(III) ion coordinates two nitrogen donors from adjacent pyrrole rings, forming two extraordinarily long Rh−N bonds (2.357(3) and 2.376(3) Å, compared to typical 1.95–2.30 Å),^[28]^ as a result of macrocyclic constrains. These constrains also affect angles around rhodium(III) center, giving rise to a very large N−Rh−N angle of 124.7(1)° in a strongly distorted octahedron CCNN(CCl). On the other hand, coordination of metal ion to two opposite N‐donors leads to reduced N⋅⋅⋅N distance (4.192(4) Å) in macrocycle **4**, compared to parent **5** (4.679(4) Å) and to strong saddle distortion of the metallaporphyrin (Table S2).

Dirhodaporphyrin, **3**, is constructed of two rhodacyclopentadiene rings and two pyrrole units incorporated in a relatively planar macrocyclic frame. Introduction of two rhodium atoms to one porphyrin skeleton in place of two NH units, reduced the number of heteroatoms potentially serving as donors per one metal center, comparing to **2** or **4**. Thus, each octahedral rhodium(III) center in **3** is surrounded by only three porphyrin donors (CCN), one carbonyl and two chloride anions shared by both metal ions (Figure 2). The binuclear dichloride‐bridged unit Rh_2_Cl_2_ is surrounded by the porphyrin N_2_C_20_ frame which is approximately perpendicular (86°) to the Rh_2_Cl_2_ plane. The Rh⋅⋅⋅Rh distance, equal 2.8668(13) Å is significantly shorter than analogous distances in octahedral binuclear rhodium complexes linked by two chloride bridges (3.42–4.01 Å),^[28]^ which can be attributed to a squeezing effect of the macrocyclic frame. Even shorter Rh⋅⋅⋅Rh distance (2.639(1) Å; X‐ray) in Rh_2_L_2_ was found in dinuclear hydridorhodium(III) complexes (L=H), for which Authors suggest absence of Rh−Rh bond on the basis of geometrical parameters.^[32]^ For **3**, in which the short distance is forced by the porphyrin environment, DFT studies do not show a significant electron density between the rhodium centers (Figure S54) and the NBO analysis did not show a localized Rh−Rh bond, thus, no metal–metal bond is claimed.

The overall shape of **3** exhibits similarity with **2** regarding the rhomboidal deformation. Importantly, the macrocyclic skeleton of 21,23‐dirhodaporphyrin **3** accommodating two large atoms is not much distorted from planarity, showing waved deformation mode with two rhodium atoms only 0.46 Å away from the C_4‐*meso*_ plane. Two heteroatoms extrusion allowed to gain more space for a bimetallic unit embedded in the [18]porphyrin perimeter.

### Spectroscopic studies

**Figure 3:**
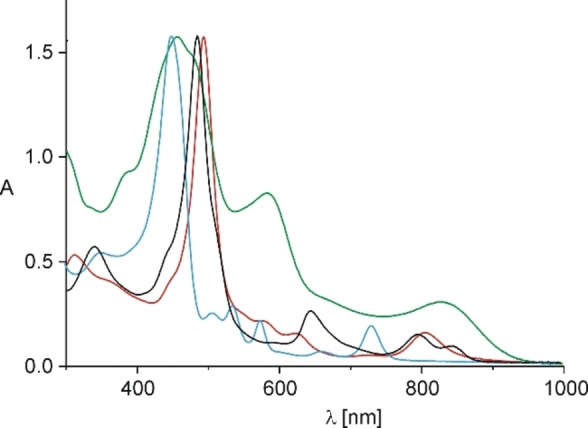
UV‐Vis electronic spectra (tetrakis(4‐methoxyphenyl) series; CH_2_Cl_2_) of **2**: red line, **2**‐H: green line, **3**: black line, **4**: blue line.

All the rhodaporphyrins exhibit spectroscopic features consistent with their aromatic character. Their solutions are deeply colored (red **2**, green **2**‐H, brown **3**, orange **4**), which is reflected in UV‐Vis electronic spectra (Figure 3), with intense Soret bands (**2**: 494 nm, **2**‐H: 467 nm, **3**: 487 nm, **4**: 453 nm) and well defined Q‐bands (Q1: **2**: 806 nm, **2**‐H: 869 nm, **3**: 845 nm, **4**: 735 nm), characteristic for aromatic heteroporphyrins.^[33]^

**Figure 4:**
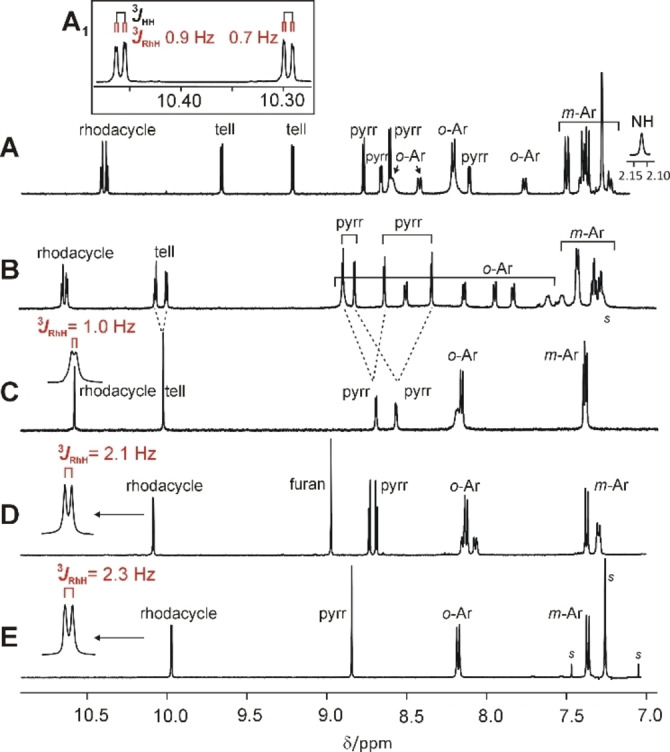
^1^H NMR (500 MHz) spectra of rhodaporphyrins; (tetrakis(4‐methoxyphenyl) series; OCH_3_ signals not shown. A) **2**‐H (CDCl_3_, 300 K, inset A_1_: [D_8_]toluene, 380 K), B) **2** (CD_2_Cl_2_, 190 K), C) **2** (CD_2_Cl_2_, 300 K), D) **4** (CD_2_Cl_2_, 300 K), E) **3** (CDCl_3_, 300 K).

^1^H NMR spectra of rhodaporphyrins (Figure 4) show large chemical shifts of all β‐protons, corresponding with the macrocyclic aromaticity. Accordingly, the inner NH proton present in **2**‐H is significantly shifted upfield. For all these species, the most downfield shifted signals (9.9–10.7 ppm) are assigned to protons of rhodacyclopentadiene subunits on the basis of their splitting by ^103^Rh nucleus, with relatively small ^3^ *J*_RhH_ coupling constants, ranging from 0.7 Hz (**2**‐H) to 2.3 Hz (**3**), characteristic for this nuclide. In case of ^13^C NMR spectra, Rh−^13^ C≡O signals show large ^1^ *J*_RhC_ coupling constants (71–75 Hz), similar to literature values for terminal carbonyls (70 Hz),^[34]^ allowing their easy discrimination from α‐rhodacycle carbon‐13 signals, appearing in the same spectral range, but featuring much smaller ^1^ *J*_RhC_ coupling constants (26–30 Hz), similar to ^1^ *J*_RhCsp2_ detected in rhodium(III) benziporphyrin complexes (23–26 Hz).^[35]^

### Fluxional behavior of rhodaporphyrins

The number of β‐proton signals is indicative of the molecular symmetry, provided that dynamic processes are absent or slow on the ^1^H NMR timescale. Thus, in ^1^H NMR spectrum of **4** the chemically equivalent pairs of β‐rhodacycle and β‐furan protons appear as ^103^Rh‐split doublet and a singlet respectively (Figure 4D), consistently with the molecule *C_s_* symmetry found in the solid state. The *C*_1_ symmetry of **2** detected in the solid state corresponds to eight different chemical environments of β‐hydrogens, and the adequate ^1^H NMR spectral pattern was observed for **2** at 190 K (Figure 4B). Presence of only four β‐hydrogen signals at room temperature (Figure 4C) indicates dynamic behavior of **2** in solution, thus variable temperature studies in 190–300 K range were performed, in order to follow the dynamic process (Figure S4). Two AB patterns visible at 190 K, assigned to rhodacyclopentadiene and tellurophene β‐hydrogens, broadened on sample heating and finally coalesced, giving two sharp singlets at 260 K, one of which split into a doublet with small ^3^ *J*_RhH_ (1.0 Hz), when the lines narrowed enough at 300 K. Similarly, four β‐pyrrole signals detected at 190 K, were dynamically averaged to two doublets, which became sharp at 300 K.

**Figure 5:**
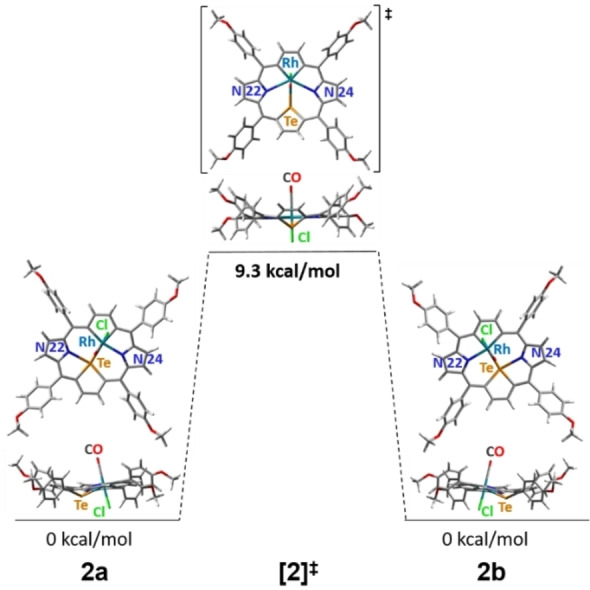
Conformers of **2** participating in the dynamic process (DFT optimized structures).

The dynamic process responsible for these observations is the fluxional behavior of **2** involving two enantiomeric forms, **2a** and **2b** (Figure 5). The rhodium(III) central ion switches between two nitrogen donors, N22 and N24, changing the equatorial coordination environment from CCTeN24 in **2a** to CCTeN22 in **2b**. This shift is accompanied by the carbon skeleton conformation changes within the macrocyclic plane. DFT optimized geometries of stationary conformers **2a** and **2b** were based on the X‐ray structure of **2**. The calculated symmetric transient form, [**2**]^≠^, with heptacoordinate rhodium(III) ion possesses a significant tellurophene tilt reflected by interplanar tellurophene–C_4‐*meso*_ angle, 36°, compared to 25° in **2a**/**b**. The calculated energy barrier of the **2a**⇌**2b** switch, 9.3 kcal/mol, is comparable to the experimental value estimated from coalescence temperatures for β‐tellurophene and β‐rhodacyclopentadiene, Δ*G* ^≠^=11.5(1) kcal/mol, and close to 11.0 kcal/mol obtained for analogous dynamics in the palladium(II) analog of **2**.^[19]^

For **2**‐H the ^1^H NMR room temperature spectrum (Figure 4A) includes eight β‐hydrogen signals, which is consistent with *C_1_* molecular symmetry found in the solid state. Neither β‐hydrogen signal averaging, nor presence of EXSY correlations on a NOESY map, confirmed conformational changes of the macrocyclic skeleton, thus, the structure can be considered rigid, with the exception for rotating aryl groups. The difference in dynamic properties between **2** and **2**‐H can be attributed to protonation of one nitrogen atom in **2**‐H, rendering this atom no longer available for metal coordination. The NH proton signal shows relatively large chemical shift of 2.13 ppm (300 K), as compared to negative *δ* values for NH hydrogens in heteroporphyrins, for example, −1.5 ppm for 21‐telluraporphyrin. It can be ascribed to formation of an intramolecular NH⋅⋅⋅Cl hydrogen bond and strong out of plane pyrrole tilt, as detected in the solid state. Analogous to **2**‐H ^1^H NMR characteristics were observed for **4**‐H (Figure S29).

**Figure 6:**
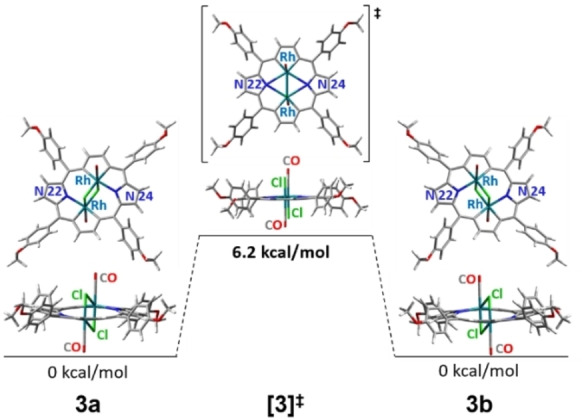
Conformers of **3** participating in the dynamic process (DFT optimized structures).

^1^H NMR spectrum of 21,23‐dirhodaporphyrin, **3**, measured at 300 K (Figure 4E), comprising of one rhodium‐split doublet (^3^ *J*_RhH_=2.3 Hz), one β‐pyrrole singlet, two aryl (*o*‐ and *m*‐) signals and one OCH_3_ peak, corresponds to a structure with higher symmetry (*D*_2h_), than obtained from the solid state studies of **3** (*C*_i_). Thus, fast at ^1^H NMR timescale dynamic behavior at two levels was assumed: 1) aryl rotation typical for porphyrins, 2) the skeleton of 21,23‐dirhodaporphyrin fluxional motion, where rhodium(III) ions switch between two nitrogen donors in a concerted movement. In order to slow down molecular motions, the temperature has been lowered and at 220 K as the aryl rotation decelerated, *o*‐ and *m*‐aryl signals doubled giving four separate signals, reflecting the differentiation of ligands on each rhodium(III) on two sides of the macrocyclic plane (Figure S20). We also awaited doubling of β‐hydrogen signals, expecting two AB patterns for β‐protons at slow exchange limit. However, even at the lowest temperature available for the solvent used (175 K, supercooled CD_2_Cl_2_), the β‐hydrogen signals did not split nor broadened, implying that the slow exchange conditions were not fulfilled. DFT studies of the **3a**⇌**3b** interconversion passing through a symmetric transient state [**3**]^≠^ were performed, addressing energetics of the processes (Figure 6).

The stationary form **3a**, optimized on the basis of X‐ray structure, switches to **3b** superimposable with **3a**. The optimized transient form [**3**]^≠^ is symmetrical and practically planar, with Rh−Rh distance shortened to 2.69 Å, as compared to 2.91 Å in **3a**/**b** (calculated values). The computed activation barrier, 6.2 kcal/mol is relatively low, and moreover, the calculated chemical shift difference between β‐protons of pyrrole (*δ*_H8_–*δ*_H7_) is only 0.05 ppm, while for β‐rhodacycle (*δ*_H2_–*δ*_H3_), even less, 0.02 ppm. Such a low energy barrier in combination with small frequency differentiation (Δ*ν* 30 and 12 Hz, respectively, at 600 MHz spectrometer), did not allow to accomplish the slow exchange limit in the available temperature range.

### Tellurium‐to‐rhodium substitution mechanism

**Scheme 4:**
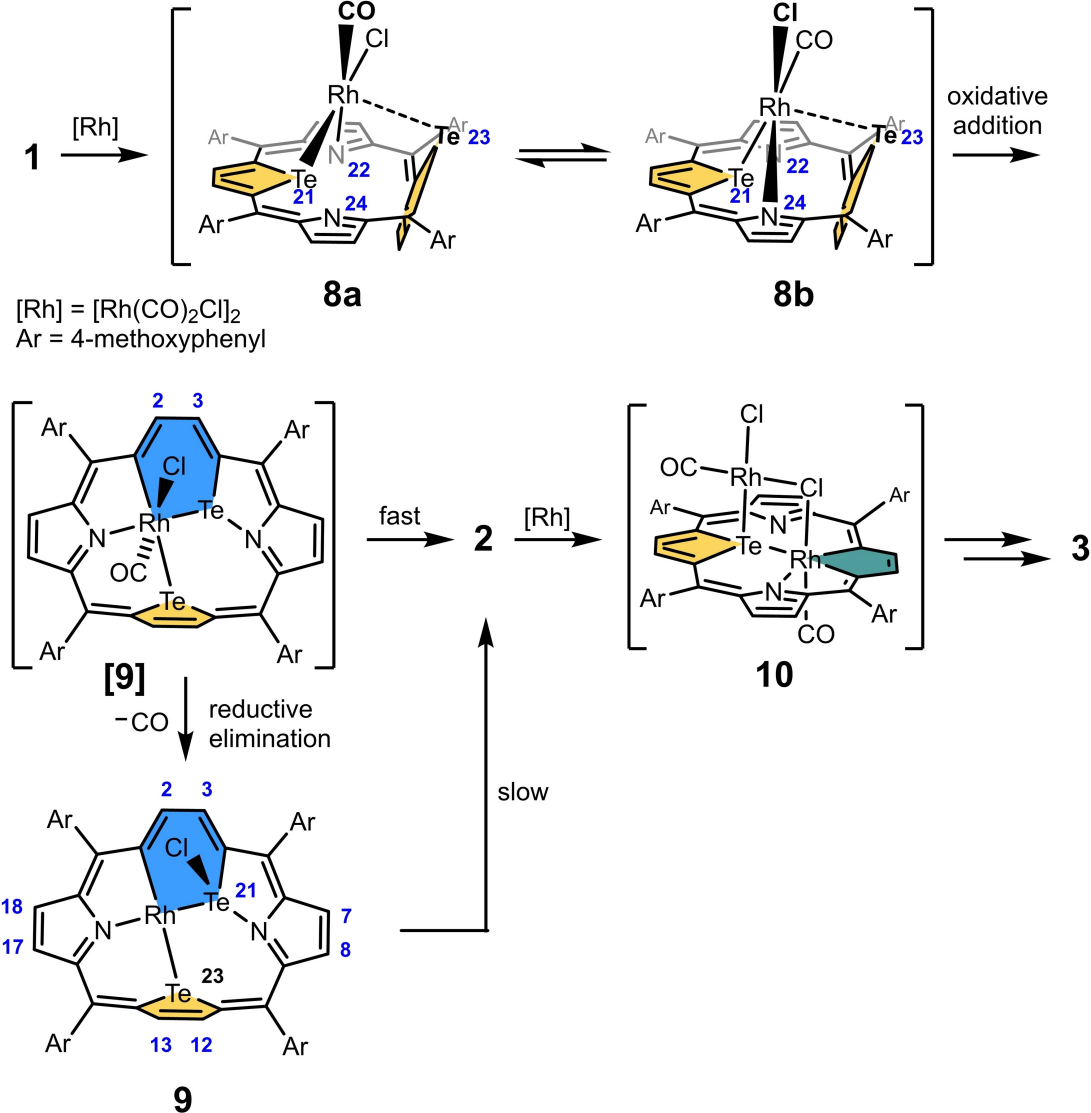
Proposed transformation mechanism.

The described tellurium to rhodium exchange proceeds more easily than analogous tellurium to palladium and tellurium to platinum substitutions.[^19^, ^20^] The transformation of **1** into **2** proceeds readily at room temperature, while direct syntheses of palladium and platinum analogs required heating to 40 °C and 110 °C respectively. High reactivity of telluraporphyrins towards rhodium(I) is also reflected by feasibility of both tellurium atoms replacement leading to formation of **3**. In consequence, intermediates of the substitution reaction are relatively reactive, however possible to trap below or at room temperature. Thus, for the reaction of **1** with [RhCl (CO)_2_]_2_ carried out at room temperature in CD_2_Cl_2_, two new reactive species, which finally transformed into **2**, were detected in ^1^H NMR spectrum (Figure S36). In order to trap the more reactive intermediate, with proposed structure **8** (Scheme 4), a mixture of **1** and [RhCl (CO)_2_]_2_ in CD_2_Cl_2_ was prepared and immediately cooled down to 280 K; formation of **8** was instant. The low temperature ^1^H NMR spectrum (180 K, Figure S34) is consistent with weak aromaticity of the molecule and the chemical shifts of β‐protons reflect the molecular geometry, that is, the presence of one Te‐inside oriented tellurophene ring (8.59 and 8.48 ppm) and the second tellurophene Te‐out pointing (7.00 and 5.87 ppm). The presence of four tellurophene signals and differentiation of *ortho*‐H signals of each aryl ring indicate *C*_1_ molecular symmetry of the complex. On temperature rising signals broaden and coalesce in pairs (Figure S36), revealing dynamic behavior of **8**, and finally the spectrum at 280 K showing four β‐H signals reflects the fast exchange condition. Above 280 K **8** is transformed into the product **2**. Considering the spectral data for **8** and assuming that an initial reaction step involves formation of a side‐on rhodium(I) complex, in analogy to previously reported palladium(II) congener exhibiting strikingly similar ^1^H NMR spectrum,^[19]^ we proposed the structure of intermediate **8** as depicted in Scheme 4. In porphyrin chemistry, similar coordination of Rh^I^Cl(CO) unit to a bidentate site of the macrocycle can be regarded as typical.[^1^, ^9^, ^36^] Variable temperature NMR features of **8** are in accord with the complex switch between two enantiomeric forms, **8a** and **8b**, where rhodium(I) ion flips between two nitrogen donors, while the Rh−Te bond is maintained. Compound **8** was subjected to DFT calculations and the reliability of the optimized structure (Figure S51) was confirmed by a good agreement of calculated and experimental ^1^H NMR chemical shifts (Figure S55). Thus, the tellurophene unit (Te23) position, almost perpendicular to the C_4‐*meso*_ plane, preventing efficient π‐delocalization, is in accord with very weak macrocyclic aromaticity of **8**, and the inverted conformation of this ring agrees with relative upfield shifts of the β‐protons.

The proposed subsequent transformation step includes rhodium(I) insertion into Te−C bond through oxidative addition, generating rhodium(III) organometallic species containing a six‐membered rhodatelluracyclohexadiene ring (RhTeC_4_). Formation of corresponding, well characterized metallatelluracycles, eventually transforming to metallaindenes, was reported for reactions of benzo[*b*]tellurophene with several metal carbonyl clusters.^[37]^ The structure [**9**] (Scheme 4), coined as a probable intermediate was subjected to DFT optimization (Figure S52), yielding a structure with tellurophene ring almost perpendicular to C_4‐*meso*_ plane (80°). On the other hand, in experiments aiming in observation of intermediates of **1** to **2** transformation, a species **9** has been tracked, with ^1^H NMR characteristics expected for a structure with the RhTeC_4_ ring. Thus, **9** was obtained from **1** and [RhCl(CO)_2_]_2_ in CDCl_3_ at room temperature under ^1^H NMR control (Figures S35 and S36). The spectrum corresponds to a compound of *C*_1_ symmetry with weak macrocyclic aromaticity, and is distinguished by the presence of a pair of β‐H signals, split with an unusual for heteroporphyrins coupling constant, ^3^ *J*_HH_=10.8 Hz (H2, H3). Such a large ^3^ *J*_HH_ value and the presence of an additional spin‐spin coupling ascribed to ^103^Rh nucleus (^3^ *J*_RhH_=1.7 Hz) in one of two signals (H2) can be explained by the presence of an expected six‐membered rhodatelluracycle, embedded in the macrocyclic structure. For metallatelluracycles ^3^ *J*_HH_ coupling constants of the same order (9.7–11.1 Hz)^[37]^ were reported, and in analogous PtSC_4_ ring a value of 12 Hz was observed.^[38]^ The chemical shift range of β‐protons in **9** (6.72–8.56), indicating weak macrocyclic aromaticity, gives evidence of a significant tilt of one heterocyclic ring, limiting the π‐electron conjugation. Despite several matching characteristics, a mismatch between the experimental (**9**) and calculated (for [**9**]) proton chemical shift, ruled out their identity (Figure S52).

We put forward the hypothesis that the reactive [**9**] is formed by an oxidative addition from **8**, and transforms to the observed **9**, preserving the six‐membered ring, the strong tellurophene tilt and low macrocyclic aromaticity. The postulated structure **9** presented in Scheme 4 possesses all these features and its reliability is supported by good accordance of the calculated and experimental proton chemical shifts (Figure S55). The molecule **9** contains rhodium(I) center in a typical square planar coordination environment, thus the transformation of [**9**] to **9**, required CO dissociation and an intramolecular reductive elimination (more in Figure S52). The reactive [**9**] may be also transformed directly to **2**, which is in accord with the observation that the formation of **9** was not always reproducible.

Experiments aiming at spectroscopic observation of intermediates of the second tellurium to rhodium exchange in **2** to **3** transformation were also performed. [RhCl(CO)_2_]_2_ has been added to a solution of **2** in CDCl_3_ at room temperature and formation of a new set of signals, attributed to an intermediate **10**, has been observed in ^1^H NMR after 5 minutes. The spectrum (Figure S37) exhibits features of an aromatic porphyrin of *C*_1_ symmetry, with chemical shifts of β‐protons in the range consistent with macrocyclic aromaticity (8.02–10.45 ppm), displaying ^3^ *J*_βHβH_ coupling constants typical for pentacyclic units built‐in a porphyrin (4.5–6.1 Hz). We may assume that the reaction **2**→**3** also begins with a side‐on binding of a Rh(I)‐containing unit (RhCl(CO)) on one face of the macrocycle with formation of a mixed‐valence adduct **10**. The product does not exhibit a detectable by NMR dynamic switch characteristic for 21‐rhoda‐23‐telluraporphyrins. Compound **10** may be transformed into **3** on heating to 110 °C in toluene, while on standing for few days in chloroform solution at room temperature it decomposes to a mixture of **2** and **2**‐H. The structure **10** was proposed assuming binding of Rh^I^Cl(CO) unit to two donor atoms available for coordination, localized on one face of **2** in a proper geometric arrangement. Among several structures taken under consideration, supported by DFT calculations (Figure S53), the one with the lowest calculated energy is shown in Scheme 4. The final reaction step, **10** to **3** transformation, required high temperature and subsequent intermediates were not detected.

## Conclusion

The 21,23‐dirhodaporphyrin, **3**, can be formally regarded as a dinuclear organometallic complex of an annulene–porphyrin hybrid. Such crossbreeds, porphyrins devoid of two nitrogen atoms, named 21,23‐divacataporphyrin^[26]^ or 21,23‐dideazaporphyrin^[39]^ are known; however, a direct metal ion insertion into these macrocycles has never been reported. Monometallic 21‐rhoda‐23‐telluraporphyrin, **2**, and 21‐oxa‐23‐rhodaporphyrin, **4**, are derived from heteroanalogs of a 21‐vacataporphyrin, capable of forming organometallic transition metal complexes, but still, the activation of two inner C−H bonds has never occurred.^[40]^

Perceived from a metallacycle perspective, metallaporphyrins may be considered as expanded metallacyclopentadienes,^[41]^ inserted in a stabilizing aromatic macrocyclic scaffold. The chemistry of metallacycles, in particular of metallacyclopentadienes has gained a lot of attention recently, owing to the electrifying discovery of several types of unconventional metal‐assisted aromaticity, including Craig‐type Möbius aromaticity.[^42^, ^43^]

In conclusion, the removal of two core donor atoms of a porphyrin allowed the introduction of a Rh_2_Cl_2_ binuclear unit into the center of the macrocyclic scaffold. The 18‐π‐electron aromatic circuit of a metallaporphyrin plays a crucial role in macrocycle stabilization, and in the case of 21,23‐dirhodaporphyrin, **3**, imposes the close proximity of the two metal ions. This unprecedented dimetallaporphyrin starts a new chapter on bimetallic porphyrinoids and opens perspectives for multimetallic derivatives formation. Bearing in mind that the efficacy and diversity of transformations mediated by rhodium porphyrins is vast and still growing,^[36]^ 21,23‐dirhodaporphyrin may be regarded as a good candidate for catalytic applications. A large catalytic potential of chloride‐bridged dinuclear rhodium(III) complexes has been recently documented.^[44]^

## Experimental Section

Experimental procedures and data are given in the Supporting Information.

Deposition Numbers 2128521 (for **2**), 2128522 (for **2**‐H), 2128523 (for **3**), 2128524 (for **4**), 2128525 (for **5**), and 2128526 (for **1**_B_) contain the supplementary crystallographic data for this paper. These data are provided free of charge by the joint Cambridge Crystallographic Data Centre and Fachinformationszentrum Karlsruhe Access Structures service.

## Conflict of interest

The authors declare no conflict of interest.

1

## Supporting information

As a service to our authors and readers, this journal provides supporting information supplied by the authors. Such materials are peer reviewed and may be re‐organized for online delivery, but are not copy‐edited or typeset. Technical support issues arising from supporting information (other than missing files) should be addressed to the authors.

Supporting Information

## Data Availability

Research data are not shared.

## References

[1] A. Takenaka , Y. Sasada , T. Omura , H. Ogoshi , Z.-I. Yashida , J. Chem. Soc. Chem. Commun. 1973, 792–793.

[2] D. V. Partyka , T. J. Robilotto , M. Zeller , A. D. Hunter , T. G. Gray , Proc. Natl. Acad. Sci. USA 2008, 105, 14293–14297. 18780788 10.1073/pnas.0806520105 PMC2567207

[3] M. Tsutsui , C. P. Hrung , D. Ostfeld , T. S. Srivastava , D. L. Cullen , E. F. Meyer , J. Am. Chem. Soc. 1975, 97, 3952–3965. 1159208 10.1021/ja00847a015

[4] J.-J. Lai , S. Khademi , E. F. Meyer , D. L. Cullen , K. M. Smith , J. Porphyrins Phthalocyanines 2001, 5, 621–627.

[5] S. Le Gac , B. Boitrel , J. Porphyrins Phthalocyanines 2016, 20, 117–133.

[6] T. Sarma , B. S. Kumar , P. K. Panda , Angew. Chem. Int. Ed. 2015, 54, 14835–14839; 10.1002/anie.201508409 26486503

[7] M. J. Białek , L. Latos-Grażyński , Chem. Commun. 2014, 50, 9270–9272. 10.1039/c4cc04271a 25002235

[8] K. K. Dailey , G. P. A. Yap , A. L. Rheingold , T. B. Rauchfuss , Angew. Chem. Int. Ed. Engl. 1996, 35, 1995–1997.

[9] A. Srinivasan , H. Furuta , A. Osuka , Chem. Commun. 2001, 1666–1667. 10.1039/b104004a 12240434

[10] M. Toganoh , T. Niino , H. Maeda , B. Andrioletti , H. Furuta , Inorg. Chem. 2006, 45, 10428–10430. 17173392 10.1021/ic061741p

[11] K. Yoshida , S. Yamaguchi , A. Osuka , H. Shinokubo , Organometallics 2010, 29, 3997–4000.

[12] Y. Rao , T. Kim , K. H. Park , F. Peng , L. Liu , Y. Liu , B. Wen , S. Liu , S. R. Kirk , L. Wu , B. Chen , M. Ma , M. Zhou , B. Yin , Y. Zhang , D. Kim , J. Song , Angew. Chem. Int. Ed. 2016, 55, 6438–6442; 10.1002/anie.201600955 27038255

[13] W. Stawski , M. Kijewska , M. Pawlicki , Chem. Asian J. 2020, 15, 8–20. 31693799 10.1002/asia.201901422

[14] J.-M. Barbe, R. Guilard, in *Porphyr. Handb*., **2000**, pp. 211–244.

[15] S. Onaka , Y. Kondo , M. Yamashita , Y. Tatematsu , Y. Kato , M. Goto , T. Ito , Inorg. Chem. 1985, 24, 1070–1076.

[16] P. J. Brothers , Chem. Commun. 2008, 2090–2102. 10.1039/b714894a 18438481

[17] A. Weiss , M. C. Hodgson , P. D. W. Boyd , W. Siebert , P. J. Brothers , Chem. Eur. J. 2007, 13, 5982–5993. 17570718 10.1002/chem.200700046

[18] J. Conradie , P. J. Brothers , A. Ghosh , Inorg. Chem. 2019, 58, 4634–4640. 30874434 10.1021/acs.inorgchem.9b00201

[19] E. Pacholska-Dudziak , M. Szczepaniak , A. Książek , L. Latos-Grażyński , Angew. Chem. Int. Ed. 2013, 52, 8898–8903; 10.1002/anie.201304493 23881715

[20] E. Pacholska-Dudziak , G. Vetter , A. Góratowska , A. Białońska , L. Latos-Grażyński , Chem. Eur. J. 2020, 26, 16011–16018. 32511814 10.1002/chem.202002677

[21] E. Pacholska , L. Latos-Grażyński , Z. Ciunik , Angew. Chem. Int. Ed. 2001, 40, 4466–4469; 10.1002/1521-3773(20011203)40:23<4466::aid-anie4466>3.0.co;2-l 12404447

[22] K. Badyal , W. R. McWhinnie , H. L. Chen , T. A. Hamor , J. Chem. Soc. Dalton Trans. 1997, 1579–1585.

[23] L. Latos-Grażyński , E. Pacholska , P. J. Chmielewski , M. M. Olmstead , A. L. Balch , Angew. Chem. Int. Ed. Engl. 1995, 34, 2252–2254.

[24] M. Abe , D. G. Hilmey , C. E. Stilts , D. K. Sukumaran , M. R. Detty , Organometallics 2002, 21, 2986–2992.

[25] I. Gupta , M. Ravikanth , Coord. Chem. Rev. 2006, 250, 468–518.

[26] E. Pacholska-Dudziak , L. Szterenberg , L. Latos-Grażyński , Chem. Eur. J. 2011, 17, 3500–3511. 21341322 10.1002/chem.201002765

[27] C.-H. Lee , H.-J. Kim , D.-W. Yoon , Bull. Korean Chem. Soc. 1999, 20, 276–280.

[28] C. R. Groom , I. J. Bruno , M. P. Lightfoot , S. C. Ward , Acta Crystallogr. Sect. B Struct. Sci. Cryst. Eng. Mater. 2016, 72, 171–179. 10.1107/S2052520616003954 PMC4822653 27048719

[29] T. Steiner , Acta Crystallogr. Sect. B 1998, 54, 456–463. 10.1107/s090744499701500x 9761853

[30] S. S. Batsanov , Inorg. Mater. 2001, 37, 871–885.

[31] S. Alvarez , Dalton Trans. 2013, 42, 8617–8636. 23632803 10.1039/c3dt50599e

[32] D. Hanke , K. Wieghardt , B. Nuber , R. S. Lu , R. K. McMullan , T. F. Koetzle , R. Bau , Inorg. Chem. 1993, 32, 4300–4305.

[33] L. Latos-Grażyński in Porphyrin Handbook (Eds.: K. M. Kadish , K. M. Smith , R. Guilard ), Academic Press, 2000, pp. 361–416.

[34] J. M. Ernsting , S. Gaemers , C. J. Elsevier , Magn. Reson. Chem. 2004, 42, 721–736. 15307053 10.1002/mrc.1439

[35] K. Hurej , M. Pawlicki , L. Szterenberg , L. Latos-Grażyński , Angew. Chem. Int. Ed. 2016, 55, 1427–1431; 10.1002/anie.201508033 26643286

[36] S. J. Thompson , M. R. Brennan , S. Y. Lee , S. J. Thompson , S. J. Thompson , M. R. Brennan , G. Dong , Chem. Soc. Rev. 2018, 47, 929–981. 29188830 10.1039/c7cs00582b

[37] A. J. Arce , A. Karam , Y. De Sanctis , R. Machado , M. V. Capparelli , J. Manzur , Inorg. Chim. Acta 1997, 254, 119–130.

[38] M. Hernández , G. Miralrio , A. Arévalo , S. Bernès , J. J. García , C. López , P. M. Maitlis , F. Del Rio , Organometallics 2001, 20, 4061–4071.

[39] T. D. Lash , S. A. Jones , G. M. Ferrence , J. Am. Chem. Soc. 2010, 132, 12786–12787. 20795621 10.1021/ja105146a

[40] E. Pacholska-Dudziak , J. Skonieczny , M. Pawlicki , L. Szterenberg , Z. Ciunik , L. Latos-Grażyński , J. Am. Chem. Soc. 2008, 130, 6182–6195. 18412350 10.1021/ja711039c

[41] W. Ma , C. Yu , T. Chen , L. Xu , W. X. Zhang , Z. Xi , Chem. Soc. Rev. 2017, 46, 1160–1192. 28119972 10.1039/c6cs00525j

[42] D. Chen , Q. Xie , J. Zhu , Acc. Chem. Res. 2019, 52, 1449–1460. 31062968 10.1021/acs.accounts.9b00092

[43] D. Chen , Y. Hua , H. Xia , Chem. Rev. 2020, 120, 12994–13086. 33073994 10.1021/acs.chemrev.0c00392

[44] Y. Kita , S. Hida , K. Higashihara , H. S. Jena , K. Higashida , K. Mashima , Angew. Chem. Int. Ed. 2016, 55, 8299–8303; 10.1002/anie.201601748 27088539

