# Supplementary material for: Two Rhodium(III) Ions Confined in a [18]Porphyrin Frame: 5,10,15,20‐Tetraaryl‐21,23‐Dirhodaporphyrin
Source: Chemistry. 2022 Jul 6;28(46):e202201513. doi: 10.1002/chem.202201513 (PMC9545270; doi:10.1002/chem.202201513)
Supplement: Supplementary file 1 — Supporting Information [file CHEM-28-0-s001.pdf]

Chemistry–A European Journal

Supporting Information

Two Rhodium(III) Ions Confined in a [18]Porphyrin Frame: 5,10,15,20-Tetraaryl-21,23-Dirhodaporphyrin

Grzegorz Vetter, Agata Białońska, Paulina Krzyszowska, Sebastian Koniarz, and Ewa Pacholska-Dudziak*

Table of Contents

Experimental Procedures 3

Figure S1. Variable temperature ^{1}H NMR spectra of $\mathbf{1_B}$; 600 MHz, CD_2Cl_2, 220–300 K. 9

Figure S2. ^{13}C NMR spectrum of $\mathbf{1_B}$; 150 MHz, CD_2Cl_2, 220 K. 10

Figure S3. HRMS ESI (+MS) spectra of $\mathbf{1_B}$: measured (top) and simulated (bottom) calc. for $C_{46}H_{30}Cl_2N_2O_2Te_2$, $[M+H]^+$. 11

Figure S4. Variable temperature ^{1}H NMR spectra of $\mathbf{2_A}$; 600 MHz, CD_2Cl_2, 190–300 K. 11

Figure S5. ^{13}C NMR spectrum of $\mathbf{2_A}$; 150 MHz, CD_2Cl_2, 190 K. 12

Figure S6. HRMS ESI (+MS) spectra of $\mathbf{2_A}$: measured (top) and simulated (bottom) calc. for $C_{48}H_{36}N_2O_4RhTe$, $[M-Cl-CO]^+$. 13

Figure S7. Variable temperature ^{1}H NMR spectra of $\mathbf{2_B}$; 600 MHz, CD_2Cl_2, 180–300 K. 13

Figure S8. ^{13}C NMR spectrum of $\mathbf{2_B}$; 150 MHz, CD_2Cl_2, 180 K. 14

Figure S9. HRMS ESI (+MS) spectra of $\mathbf{2_B}$: measured (top) and simulated (bottom) calc. for $C_{46}H_{30}N_2O_2Cl_2RhTe$, $[M-Cl-CO]^+$. 15

Figure S10. Variable temperature ^{1}H NMR spectra of $\mathbf{2_{B'}}$; 600 MHz, CD_2Cl_2, 180–300 K. 15

Figure S11. ^{13}C NMR spectrum of $\mathbf{2_{B'}}$; 150 MHz, CD_2Cl_2, 180 K. 16

Figure S12. HRMS ESI (+MS) spectra of $\mathbf{2_{B'}}$: measured (top) and simulated (bottom) calc. for $C_{46}H_{30}N_2O_2Cl_2RhTe$, $[M-Cl-CO]^+$. 17

Figure S13. ^{13}C NMR spectrum of $\mathbf{2_A}$**-H**; 150 MHz, $CDCl_3$, 300 K. 18

Figure S14. HRMS ESI (+MS) spectra of $\mathbf{2_A}$**-H**: measured (top) and simulated (bottom) calc. for $C_{48}H_{37}Cl_2N_2O_4RhTe$, $[M+Na]^+$. 19

Figure S15. ^{1}H NMR spectra of $\mathbf{2_B}$**-H** (bottom) and $\mathbf{2_{B'}}$**-H** (top); 500 MHz, $CDCl_3$, 300 K. 19

Figure S16. ^{13}C NMR spectrum of $\mathbf{2_B}$**-H**; 150 MHz, $CDCl_3$, 300 K. 20

Figure S17. HRMS ESI (+MS) spectra of $\mathbf{2_B}$**-H**: measured (top) and simulated (bottom) calc. for $C_{46}H_{31}Cl_4N_2O_2RhTe$, $[M+Na]^+$. 21

Figure S18. ^{13}C NMR spectrum of $\mathbf{2_{B'}}$**-H**; 150 MHz, $CDCl_3$, 300 K. 22

Figure S19. HRMS ESI (+MS) spectra of $\mathbf{2_{B'}}$**-H**: measured (top) and simulated (bottom) calc. for $C_{46}H_{31}Cl_4N_2O_2RhTe$, $[M+Na]^+$. 23

Figure S20. Variable temperature ^{1}H NMR spectra of $\mathbf{3_A}$; 600 MHz, CD_2Cl_2, 180–300 K. 23

Figure S21. ^{13}C NMR spectrum of $\mathbf{3_A}$; 150 MHz, $CDCl_3$, 300 K. 24

Figure S22. HRMS ESI (+MS) spectra of $\mathbf{3_A}$: measured (top) and simulated (bottom) calc. for $C_{50}H_{36}N_2O_6Cl_2Rh_2$, $[M+Na]^+$. 25

Figure S23. ^{1}H NMR spectrum of $\mathbf{3_B}$; 600 MHz, $CDCl_3$, 300 K. 25

Figure S24. ^{13}C NMR spectrum of $\mathbf{3_B}$; 150 MHz, $CDCl_3$, 300 K. 26

Figure S25. HRMS ESI (+MS) spectra of $\mathbf{3_B}$: measured (top) and simulated (bottom) calc. for $C_{48}H_{30}N_2O_4Cl_4Rh_2$, $[M+Na]^+$. 27

Figure S26. Variable temperature ^{1}H NMR spectra of $\mathbf{4_A}$; 600 MHz, CD_2Cl_2, 220 K and 300 K. 27

Figure S27. ^{13}C NMR spectrum of $\mathbf{4_A}$; 150 MHz, $CDCl_3$, 300 K. 28

Figure S28. HRMS ESI (+MS) spectra of $\mathbf{4_A}$: measured (top) and simulated (bottom) calc. for $C_{49}H_{36}ClN_2O_6Rh$, $[M+H]^+$. 29

Figure S29. ^{1}H NMR spectrum of $\mathbf{4_A}$**-H**; 500 MHz, $CDCl_3$, 300 K. 29

Figure S30. HRMS ESI (+MS) spectra of $\mathbf{4_A}$**-H**: measured (top) and simulated (bottom) calc. for $C_{48}H_{37}Cl_2N_2O_5Rh$, $[M+Na]^+$. 30

Figure S31. ^{1}H NMR spectra of $\mathbf{5_A}$ and $\mathbf{5_B}$; 500 MHz, $CDCl_3$, 300 K. 30

Figure S32. ^{13}C NMR spectrum of $\mathbf{5_B}$; 125 MHz, $CDCl_3$, 300 K. 31

Figure S33. HRMS ESI (+MS) spectra of $\mathbf{5_B}$: measured (top) and simulated (bottom) calc. for $C_{48}H_{36}N_2O_3Te$, $[M+H]^+$. 32

Figure S34. Variable temperature ^{1}H NMR spectra of **8**; 600 MHz, CD_2Cl_2, 180–280 K. 32

Figure S35. ^{1}H NMR spectra of **9**; 500 MHz, $CDCl_3$, 300 K. 33

Figure S36. ^{1}H NMR monitored transformation **8** $\rightarrow$ **9** $\rightarrow$ $\mathbf{2_A}$; the most informative spectrum window is chosen; 500 MHz, CD_2Cl_2, 300 K. 33

Figure S37. ^{1}H NMR spectrum of **10**; 500 MHz, $CDCl_3$, 300 K. 34
Figure S38. Absorption spectra (CH_2Cl_2) of $\mathbf{1_B}$, $\mathbf{5_B}$, **10**; $\mathbf{2_B}$, $\mathbf{2_{B'}}$, $\mathbf{3_B}$; $\mathbf{2_B}$**-H**, $\mathbf{2_{B'}}$**-H**, $\mathbf{4_A}$**-H**. 35
X-ray crystallography 36
Table S1. Crystal data for $\mathbf{2_A}$, $\mathbf{2_A}$**-H**, $\mathbf{3_A}$, $\mathbf{4_A}$, $\mathbf{5_B}$, $\mathbf{1_B}$. 36
Figure S39. A: X-ray molecular structure of $\mathbf{2_A}$. **B**: Comparison of the X-ray (red) and DFT (blue) structures. 37
Figure S40. A: X-ray molecular structure of $\mathbf{2_A}$**-H**. **B**: Comparison of the X-ray (red) and DFT (blue) structures. 38
Figure S41. **A**: X-ray molecular structure of $\mathbf{3_A}$. **B**: Comparison of the X-ray (red) and DFT (blue) structures. 38
Figure S42. **A:** X-ray molecular structure of $\mathbf{4_A}$. **B**: Comparison of the X-ray (red) and DFT (blue-white) structures. 39
Figure S43. X-ray molecular structure of **5.** 39
Figure S44. X-ray molecular structure of $\mathbf{1_B}$**.** 39
Scheme S1. Numbering scheme for X-ray data of $\mathbf{2_A}$, $\mathbf{2_A}$**-H**, $\mathbf{3_A}$, and $\mathbf{4_A}$. 40
Table S2. Selected geometric parameters for X-ray structures and DFT-optimized structures of $\mathbf{2_A}$, $\mathbf{2_A}$**-H**, $\mathbf{3_A}$, and $\mathbf{4_A}$. 40
Table S3. Selected bond lenghts and distances (Å) for X-ray structures and DFT-optimized structures of $\mathbf{2_A}$, $\mathbf{2_A}$**-H**, $\mathbf{3_A}$, and $\mathbf{4_A}$. 41
Density Functional Theory Calculations 41
Calculations for 2 42
Figure S45. DFT-optimized structure of $\mathbf{2_A}$**.** 42
Figure S46. DFT-optimized structure of $\mathbf{2_A}$**-H**. 43
Figure S47. DFT-optimized structure of $\mathbf{3_A}$. 43
Figure S48. DFT-optimized structure of $\mathbf{4_A}$. 43
Figure S49. DFT-optimized structure of $\mathbf{4_A}$**-H**. 43
Figure S50. Comparison of **2** (red) and **2-H** (blue-white) geometry (DFT structures). 44
Calculations for 8 44
Figure S51. DFT-optimized structure of **8** and **8**-1. 44
Calculations for [9] and 9. 45
Figure S52. DFT-optimized structure of **[9]**, **[9**-1**]** and **9**. 45
Calculations for 10 46
Figure S53. DFT-optimized structures of **10** and its chosen isomers. 46
Figure S54. Total electron density for $\mathbf{2_A}$, $\mathbf{2_A}$**-H**, $\mathbf{3_A}$, $\mathbf{4_A}$ and **8**. 47
Figure S55. Calculated vs experimental ^{1}H NMR shifts for $\mathbf{4_A}$**-H, 8**, **8**-1**, 10** and **10**-1. 47
Table S4. Cartesian coordinates for $\mathbf{2_A}$, $\mathbf{2_A}$**-H**, $\mathbf{3_A}$, $\mathbf{4_A}$, $\mathbf{4_A}$**-H**, several possible isomers of **8** , **9** and **10**. 48
Table S5. Transition states cartesian coordinates: $\mathbf{2_A}^{\ddagger}$, $\mathbf{3_A}^{\ddagger}$. 58
References 60

Experimental Procedures

In syntheses of rhodaporphyrins two differently *meso*-substituted tetraaryl-21,23-ditelluraporphyrins **1** were used as tellurium-containing precursors: 5,10,15,20-tetrakis(4-methoxyphenyl)-substituted $\mathbf{1_A}$ and 5,20-bis(4-chlorophenyl)-10,15-bis(4-methoxyphenyl)-substituted $\mathbf{1_B}$, leading to two series of products **2**, **2-H** and **3**, while product $\mathbf{4_B}$ has not been detected. For products **2** and **2-H** with differentiated aryls, the rhodacyclopentadiene may be formed from the tellurophene ring situated between two chlorophenyl substituents or two methoxyphenyl rings in different yields, giving doubled number of products (**B** and **B'**). Most of the work presented here has been performed for tetrakis(4-methoxyphenyl) derivatives.

Synthesis of 10,15-bis(4-chlorophenyl)-5,20-bis(4-methoxyphenyl)-21,23-ditelluraporphyrin, $\mathbf{1_B}$ was performed according to the literature.[1] 2,5-Bis(4-chlorophenylhydroxymethyl)tellurophene was synthesised from 2,5-dilithiotellurophene[2] by analogy to 2,5-bis(arylhydroxymethyl)thiophene.[3]

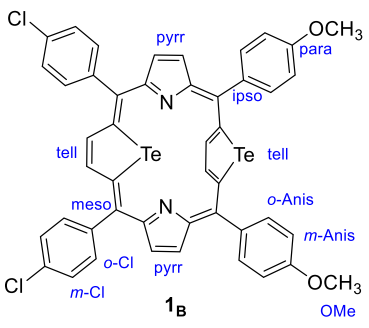

^{1}H NMR ($CDCl_3$, <u>300 K</u>, 500 MHz): δ 8.24 (br., 2H, tell), 8.04 (m, 4H, *o*-Anis), 7.92 (d, $^3J_{HH}$ = 4.5 Hz, 2H, pyrr), 7.62 (m, 4H, *o*/*m*-Cl), 7.55 (m, 4H, *o*/*m*-Cl), 7.46 (d, $^3J_{HH}$ = 4.2 Hz, 2H, pyrr), 7.10 (m, 4H, *m*-Anis), 6.34 (br., 2H, tell), 3.94 (s, 6H, OMe); **^{1}H NMR (CD_2Cl_2, <u>220 K</u>, 600 MHz):** δ 8.35 (s, 2H, tell), 8.06 (m, 4H, *o*-Anis), 7.97 (d, $^3J_{HH}$ = 4.5 Hz, 2H, pyrr), 7.63 (m, 2H, *m*-Cl), 7.57 (m, 2H, *m*-Cl), 7.53 (m, 4H, *o*-Cl), 7.45 (d, $^3J_{HH}$ = 4.5 Hz, 2H, pyrr), 7.10 (m, 4H, *m*-Anis), 6.22 (s, 2H, tell), 3.90 (s, 6H, OMe); **^{13}C NMR (CD_2Cl_2, 220 K, 150 MHz)**: δ 165.8 (α-pyrr), 161.1 (para-Anis), 158.8 (β-tell), 156.7 (α-tell), 152.2 (α-pyrr), 150.6 (α-tell), 144.4 (ipso), 141.9 (β-tell), 137.6 (meso), 137.5 (para-Cl), 135.4 (*o*-Anis), 134.3 (meso), 134.1 (β-pyrr), 133.1 (*o*-Cl), 133.0 (*o*-Cl), 132.9 (ipso), 131.0 (β-pyrr), 128.2 (*m*-Cl), 128.0 (*m*-Cl), 114.0 (*m*-Anis), 55.8 (OMe); **UV-Vis (nm, logε)** 676 (4.3), 470 (4.6), 370 (4.4); **HRMS (ESI)** *m/z* = 972.9894, calc. for $C_{46}H_{30}Cl_2N_2O_2{}^{130}Te_2$, $[M+H]^+$: 972.9835.

Synthesis of 5,10,15,20-tetrakis(4-methoxyphenyl)-21-carbonyl-21-chloro-21-rhoda-23-telluraporphyrin, $\mathbf{2_A}$: 5,10,15,20-tetrakis(4-methoxyphenyl)-21,23-ditelluraporphyrin, $\mathbf{1_A}$ (40 mg, $4.2\cdot10^{-5}$ mol) and $[Rh(CO)_2Cl]_2$ (16.2 mg, $4.2\cdot10^{-5}$ mol) were dissolved in 15 ml of toluene. Nitrogen was bubbled through the mixture for 15 minutes and the solution was refluxed for another 30 minutes. The solvent was evaporated and the products were purified by column chromatography on SiO_2 with CH_2Cl_2; $\mathbf{3_A}$ was eluted as the first brown band (2.8% yield), $\mathbf{2_A}$ as the second red band (20%), $\mathbf{2_A}$**-H** as the third green band (3.3%) and $\mathbf{4_A}$ as the fourth orange band (0.8%).
Synthesis of 5,20-bis(4-chlorophenyl)-10,15-bis(4-methoxyphenyl)-21-carbonyl-21-chloro-21-rhoda-23-telluraporphyrin, $\mathbf{2_B}$ and 10,15-bis(4-chlorophenyl)-5,20-bis(4-methoxyphenyl)-21-carbonyl-21-chloro-21-rhoda-23-telluraporphyrin $\mathbf{2_{B'}}$: The procedure was identical as for $\mathbf{2_B}$. $\mathbf{3_B}$ was eluted as the first brown band (9% yield), $\mathbf{2_B}$ as the second red band (11%), $\mathbf{2_B}$**-H** as the third brown band (0.7%), $\mathbf{2_{B'}}$ as the fourth dark red band (31%), and $\mathbf{2_{B'}}$**-H** as the fifth celadon band (1.6%).

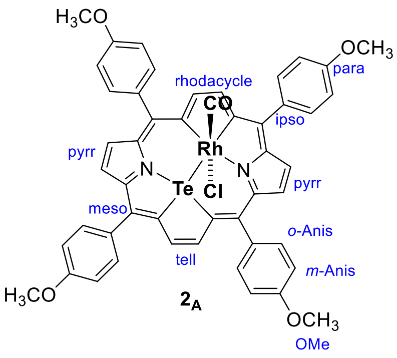

$\mathbf{2_A}$: **^{1}H NMR ($CDCl_3$, <u>300 K</u>, 500 MHz):** δ 10.59 (d, $^3J_{RhH}$ = 1.0 Hz, 2H, rhodacycle), 9.98 (s, 2H, tell), 8.65 (d, $^3J_{HH}$ = 4.5 Hz, 2H, pyrr), 8.55 (d, $^3J_{HH}$ = 4.5 Hz, 2H, pyrr), 8.16 (m, 8H, *o*-Anis), 7.35 (m, 8H, *m*-Anis), 4.091 (s, 6H, OMe), 4.090 (s, 6H, OMe); **^{1}H NMR (CD_2Cl_2, <u>190 K</u>, 600 MHz):** δ 10.65 (d, $^3J_{HH}$ = 4.8 Hz, 1H, rhodacycle), 10.62 (d, $^3J_{HH}$ = 4.8 Hz, 1H, rhodacycle), 10.07 (d, $^3J_{HH}$ = 6.0 Hz, 1H, tell), 10.00 (d, $^3J_{HH}$ = 6.0 Hz, 1H, tell), 8.89 (m, 2H, pyrr, *o*-Anis), 8.82 (d, $^3J_{HH}$ = 4,5 Hz, 1H, pyrr), 8.62 (d, $^3J_{HH}$ = 4.0 Hz, 1H, pyrr), 8.50 (m, 1H, *o*-Anis), 8.33 (d, $^3J_{HH}$ = 4.2 Hz, 1H, pyrr), 8.13 (m, 1H, *o*-Anis), 7.94 (m, 1H, *o*-Anis), 7.82 (m, 1H, *o*-Anis), 7.60 (m, 1H, *o*-Anis), 7.51 (m, 1H, *o*-Anis), 7.42 (m, 4H, *o*/*m*-Anis), 7.33–7.27 (m, 5H, *m*-Anis), 4.04 (br. s, 6H, OMe), 4.02 (s, 3H, OMe), 3.99 (s, 3H, OMe); **^{13}C NMR (CD_2Cl_2, 190 K, 150 MHz)**: δ 196.3 (d, $^1J_{RhC}$ = 26 Hz, α-

rhodacycle), 176.2 (d, $^1J_{RhCO}$ = 71 Hz, CO), 168.5 (d, $^1J_{RhC}$ = 26 Hz, α-rhodacycle), 167.1 (α-pyrr), 161.7 (β-rhodacycle), 159.6 (para), 159.4 (para), 159.2 (para), 158.5 (para), 157.1 (α-pyrr), 156.5 (α-tell), 153.3 (α-pyrr), 147.2 (α-pyrr), 146.8 (β-rhodacycle), 145.8 (β-tell), 144.3 (meso), 143.8 (meso), 143.6 (meso), 139.6 (β-pyrr), 139.5 (α-tell), 138.7 (β-tell), 137.3 (*o*-Anis), 136.7 (*o*-Anis), 136.0 (β-pyrr), 135.9 (ipso), 135.7 (*o*-Anis), 135.1 (*o*-Anis), 135.0 (*o*-Anis), 134.6 (*o*-Anis), 129.6 (β-pyrr), 129.1 (β-pyrr), 129.0 (ipso), 114.2 (*o*/*m*-Anis), 112.7 (*m*-Anis), 112.2 (*m*-Anis), 112.0 (*m*-Anis), 111.8 (*m*-Anis), 55.6 (2x, OMe), 55.5 (2x, OMe); **UV-Vis (nm, logε)**: 806 (3.8), 623 (3.8), 576 (3.9), 494 (4.8), 311 (4.3); **HRMS (ESI)** *m/z* = 937.0785, calc. for $C_{48}H_{36}N_2O_4Rh^{130}Te$, [M-Cl-CO]$^+$: 937.0787.

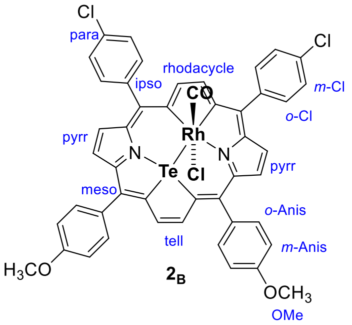

2_B: **^{1}H NMR (CD_2Cl_2, <u>300 K</u>, 600 MHz):** δ 10.44 (d, $^3J_{RhH}$ = 1.2 Hz, 2H, rhodacycle), 10.01 (s, 2H, tell), 8.70 (d, $^3J_{HH}$ = 4.6 Hz, 2H, pyrr), 8.51 (d, $^3J_{HH}$ = 4.6 Hz, 2H, pyrr), 8.18 (m, 4H, *o*-Anis), 8.16 (m, 4H, *o*-Cl), 7.82 (m, 4H, *m*-Cl), 7.39 (m, 4H, *m*-Anis), 4.08 (brs, 6H, OMe); **^{1}H NMR (CD_2Cl_2, <u>180 K</u>, 600 MHz):** δ 10.54 (d, $^3J_{HH}$ = 4.7 Hz, 1H, rhodacycle), 10.47 (d, $^3J_{HH}$ = 4.7 Hz, 1H, rhodacycle), 10.03 (d, $^3J_{HH}$ = 5.9 Hz, 1H, tell), 9.97 (d, $^3J_{HH}$ = 5.9 Hz, 1H, tell), 8.88 (m, 2H, pyrr, *o*-Cl), 8.74 (d, $^3J_{HH}$ = 4.5 Hz, 1H, pyrr), 8.63 (d, $^3J_{HH}$ = 4.0 Hz, 1H, pyrr), 8.54 (br, 1H, *o*-Cl), 8.47 (m, 1H, *o*-Anis), 8.29 (d, $^3J_{HH}$ = 4.0 Hz, 1H, pyrr), 8.14 (m, 1H, *o*-Cl), 8.03 (br, 1H, *o*-Cl), 7.93 (m, 1H, *m*-Cl), 7.84 (m, 3H, *o*-Anis, *m*-Cl), 7.76 (m, 1H, *m*-Cl), 7.71 (m, 1H, *m*-Cl), 7.60 (m, 1H, *o*-Anis), 7.50 (m, 1H, *m*-Anis), 7.41 (m, 1H, *m*-Anis), 7.31 (m, 1H, *m*-Anis), 7.26 (m, 1H, *m*-Anis), 4.04 (s, 3H, OMe), 3.98 (s, 3H, OMe); **^{13}C NMR (CD_2Cl_2, 180 K, 150 MHz)**: δ 196.3 (α-rhodacycle), 176.0 (d, $^1J_{RhCO}$ = 70 Hz, CO), 167.9 (α-rhodacycle), 167.4 (α-pyrr), 161.4 (β-rhodacycle), 159.6 (para-Anis), 159.4 (para-Anis), 157.2 (α-tell), 156.6 (α-pyrr), 152.9 (α-pyrr), 147.2 (α-pyrr), 146.1 (β-rhodacycle, β-tell), 144.9 (meso), 144.3 (α-tell), 141.8 (2x, meso, para-Cl), 141.2 (para-Cl), 140.3 (meso), 139.9 (β-pyrr), 139.3 (β-tell), 137.5 (*o*-Anis), 136.9 (*o*-Anis), 135.8 (*o*-Anis), 135.6 (β-pyrr), 135.2 (*o*-Cl), 134.7 (*o*-Cl), 134.3 (*m*-Cl), 133.7 (ipso), 133.0 (ipso), 130.1 (β-pyrr), 128.9 (*m*-Cl), 128.8 (β-pyrr), 128.6 (ipso), 127.0 (*m*-Cl), 126.8 (*m*-Cl), 114.3 (m-Anis), 114.0 (*m*-Anis), 112.7 (*m*-Anis), 112.1 (*m*-Anis), 55.6 (OMe), 55.5 (OMe); **UV-Vis (nm, logε)**: 798 (3.9), 621 (3.9), 570 (4.0), 491 (4.9), 314 (4.5); **HRMS (ESI)** *m/z* = 944.9679, calc. for $C_{46}H_{30}N_2O_2Cl_2Rh^{130}Te$, [M-Cl-CO]$^+$: 944.9796.

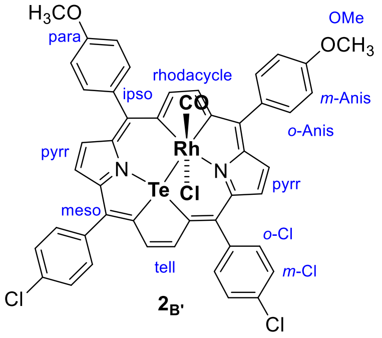

$2_{B'}$: **^{1}H NMR (CD_2Cl_2, <u>300 K</u>, 600 MHz):** δ 10.71 (d, $^3J_{RhH}$ = 1.1 Hz, 2H, rhodacycle), 10.03 (s, 2H, tell), 8.65 (d, $^3J_{HH}$ = 4.5 Hz, 2H, pyrr), 8.62 (d, $^3J_{HH}$ = 4.5 Hz, 2H, pyrr), 8.18 (m, 8H, *o*-Anis/Cl), 7.84 (m, 4H, *m*-Cl), 7.40 (m, 4H, *m*-Anis), 4.09 (brs, 6H, OMe); **^{1}H NMR (CD_2Cl_2, <u>180 K</u>, 600 MHz):** δ 10.79 (d, $^3J_{HH}$ = 4.6 Hz, 1H, rhodacycle), 10.77 (d, $^3J_{HH}$ = 4.6 Hz, 1H, rhodacycle), 10.10 (d, $^3J_{HH}$ = 6.2 Hz, 1H, tell), 10.01 (d, $^3J_{HH}$ = 6,2 Hz, 1H, tell), 8.91 (d, $^3J_{HH}$ = 4.5 Hz, 1H, pyrr), 8.88 (m, 2H, pyrr, *o*-Cl), 8.58 (d, $^3J_{HH}$ = 4.1 Hz, 1H, pyrr), 8.51 (m, 1H, *o*-Anis), 8.35 (d, $^3J_{HH}$ = 4.1 Hz, 1H, pyrr), 8.13 (m, 1H, *o*-Anis), 8.01 (m, 1H, *o*-Anis), 7.97 (m, 1H, *o*-Cl), 7.87 (m, 1H, *o*-Anis), 7.77 (m, 2H, *m*-Cl), 7.70 (m, 1H, *m*-Cl), 7.55 (m, 1H, *m*-Cl), 7.44 (m, 2H, *m*-Anis), 7.32 (m, 2H, *m*-Anis), 4.05 (s, 3H, OMe), 4.02 (s, 3H, OMe); **^{13}C NMR (CD_2Cl_2, 180 K, 150 MHz)**: δ 197.2 (α-rhodacycle), 175.6 (d, $^1J_{RhCO}$ = 71 Hz, CO), 169.4 (α-rhodacycle), 166.7 (α-pyrr), 162.6 (β-rhodacycle), 159.2 (para-Anis), 158.5 (para-Anis), 157.3 (α-pyrr), 155.4 (α-tell), 153.9 (α-pyrr), 147.9 (β-rhodacycle), 147.0 (α-pyrr), 145.5 (β-tell), 144.7 (meso), 144.4 (meso), 142.8 (α-tell), 142.3 (para-Cl), 141.2 (para-Cl), 139.2 (β-pyrr), 138.0 (β-tell), 136.5 (2x, β-pyrr, *m*-Cl), 136.0 (*o*-Anis), 135.4 (*o*-Anis), 135.2 (*m*-Cl), 135.0 (ipso), 134.9 (*o*-Cl), 134.8 (*o*-Anis), 134.3 (ipso), 134.1 (ipso), 131.0 (*m*-Cl), 129.6 (β-pyrr), 129.0 (β-pyrr), 128.9 (*m*-Cl), 128.8 (*o*-Cl), 127.5 (*o*-Anis), 127.0 (*m*-Cl), 114.2 (*m*-Anis), 111.9 (*m*-Anis), 111.8 (*m*-Anis), 55.5 (OMe), 55.4 (OMe); **UV-Vis (nm, logε)**: 796 (3.9), 626 (3.9), 577 (3.9), 493 (4.9), 313 (4.4); **HRMS (ESI)** *m/z* = 944.9696, calc. for $C_{46}H_{30}N_2O_2Cl_2Rh^{130}Te$, [M-Cl-CO]$^+$: 944.9796.

Synthesis of 5,10,15,20-tetrakis(4-methoxyphenyl)-21,21-dichloro-21-rhoda-23-telluraporphyrin, 2_A-H: Solution of **2_A** (5 mg) in $CDCl_3$ was purged with HCl(g) for one minute and after two weeks the

transformation of **2**$_{A}$ into **2**$_{A}$**-H** was complete. The reaction was conducted under ^{1}H NMR control ($CDCl_3$, 300 K).
2$_{B}$**-H** and **2**$_{B'}$**-H** were synthesized from **2**$_{B}$ and **2**$_{B'}$ in the same way.
Conversion of 2$_{A}$**-H into 2**$_{A}$: Solution of **2**$_{A}$**-H** (3 mg) in tuluene was purged with CO(g) in the presence of 50 mg K_2CO_3. After 30 min the transformation was complete.

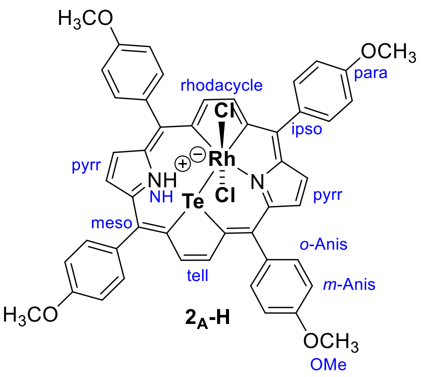

2$_{A}$**-H**: **^{1}H NMR (CDCl$_3$, 300 K, 500 MHz):** δ 10.39 (d, $^{3}J_{HH}$ = 5.1 Hz, 1H, rhodacycle*), 10.36 (d, $^{3}J_{HH}$ = 5.1 Hz, 1H, rhodacycle*), 9.64 (d, $^{3}J_{HH}$ = 5.2 Hz, 1H, tell), 9.19 (d, $^{3}J_{HH}$ = 5.2 Hz, 1H, tell), 8.75 (d, $^{3}J_{HH}$ = 5.1 Hz, 1H, pyrr), 8.64 (dd, $^{3}J_{HH}$ = 4.7 Hz, $^{4}J_{HH}$ = 2.1 Hz, 1H, pyrr), 8.59 (d, $^{3}J_{HH}$ = 4.9 Hz, 1H, pyrr), 8.57 (m, 2H, *o*-Anis) 8.40 (m, 1H, *o*-Anis), 8.19 (m, 4H, *o*-Anis), 8.09 (dd, $^{3}J_{HH}$ = 4.7 Hz, $^{4}J_{HH}$ = 2.1 Hz, 1H, pyrr), 7.74 (m, 1H, *o*-Anis), 7.48 (m, 2H, *m*-Anis), 7.39 (m, 1H, *m*-Anis), 7.38 (m, 2H, *m*-Anis), 7.35 (m, 2H, *m*-Anis), 7.21 (m, 1H, *m*-Anis), 4.11 (two s, 6H, OMe), 4.08 (s, 3H OMe), 4.06 (s, 3H OMe), 2.12 (br. s, 1H, NH); **^{13}C NMR (CDCl$_3$, 300 K, 150 MHz)**: δ 218.9 (d, $^{1}J_{RhC}$ = 27 Hz, α-rhodacycle), 193.1 (d, $^{1}J_{RhC}$ = 30 Hz, α-rhodacycle), 171.6 (α-pyrr), 166.0 (β-rhodacycle), 162.2 (para), 160.9 (para), 160.1 (2x, para), 154.3 (α-tell), 152.9 (α-tell), 152.7 (β-rhodacycle), 152.1 (α-pyrr), 150.1 (α-pyrr), 146.9 (meso), 146.2 (meso), 145.9 (α-pyrr), 144.4 (meso), 144.3 (β-tell), 143.8 (β-tell), 140.5 (β-pyrr), 138.8 (*o*-Anis), 138.4 (meso), 137.7 (*o*-Anis), 137.4 (*o*-Anis), 136.0 (*o*-Anis), 135.6 (ipso), 135.2 (*o*-Anis), 135.1 (*o*-Anis), 132.9 (ipso), 131.8 (β-pyrr), 129.7 (ipso), 128.6 (β-pyrr), 125.7 (β-pyrr), 115.5 (*m*-Anis), 114.3 (*m*-Anis), 113.5 (*m*-Anis), 113.1 (*m*-Anis), 56.0 (OMe), 55.9 (OMe), 55.8 (OMe), 55.7 (OMe); **UV-Vis (nm, logε):** 869 (3.6), 604 (4.0), 467 (4.3); **HRMS (ESI)** *m/z* = 1032.0255, calc. for $C_{48}H_{37}Cl_2N_2O_4Rh^{130}Te$, $[M+Na]^+$: 1032.0173.

*^{103}Rh splitting of the rhodacyclopentadiene signals were detected in following conditions: C_7D_6, 380 K, 600 MHz: 10.46 (dd, $^{3}J_{RhH}$ = 0.9 Hz, $^{3}J_{HH}$ = 5.1 Hz), 10.30 (dd, $^{3}J_{RhH}$ = 0.7 Hz, $^{3}J_{HH}$ = 5.1 Hz).

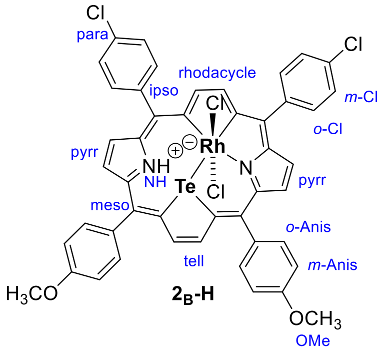

2$_{B}$**-H**: **^{1}H NMR (CDCl$_3$, 300 K, 500 MHz)**: δ 10.17 (AB, 2H, rhodacycle), 9.60 (d, $^{3}J_{HH}$ = 5.2 Hz, 1H, tell), 9.12 (d, $^{3}J_{HH}$ = 5.2 Hz, 1H, tell), 8.72 (d, $^{3}J_{HH}$ = 5.1 Hz, 1H, pyrr), 8.64 (dd, $^{3}J_{HH}$ = 4.7 Hz, $^{4}J_{HH}$ = 2.0 Hz, 1H, pyrr), 8.55 (m, 2H, *o*-Anis), 8.47 (d, $^{3}J_{HH}$ = 5.1 Hz, 1H, pyrr), 8.34 (m, 1H, *o*-Cl), 8.15 (m, 4H, *o*-Anis/Cl), 8.02 (dd, $^{3}J_{HH}$ = 4.7 Hz, $^{4}J_{HH}$ = 2.0 Hz, 1H, pyrr), 7.82 (m, 1H, *m*-Cl), 7.78 (m, 2H, *m*-Cl), 7.75 (m, 1H, *o*-Cl), 7.65 (m, 1H, *m*-Cl), 7.48 (m, 2H, *m*-Anis), 7.37 (m, 2H, *m*-Anis), 4.11 (s, 3H, OMe), 4.10 (s, 3H, OMe), 2.48 (brs, 1H, NH); **^{13}C NMR (CDCl$_3$, 300 K, 150 MHz)**: δ 218.2 ($^{1}J_{RhC}$ = 27 Hz, α-rhodacycle), 193.3 ($^{1}J_{RhC}$ = 30 Hz, α-rhodacycle), 171.9 (α-pyrr), 164,7 (β-rhodacycle), 162.6 (para-Anis), 161.1 (para-Anis), 155.6 (α-tell), 153.7 (α-tell), 152.5 (β-rhodacycle), 152.1 (α-pyrr), 149.7 (α-pyrr), 145.7 (α-pyrr), 145.0 (meso), 144.3 (2x, meso, β-tell), 144.2 (meso), 144.0 (β-tell), 140.9 (β-pyrr), 140.7 (para-Cl), 139.9 (meso), 137.9 (*o*-Cl), 137.7 (*o*-Anis), 137.6 (*o*-Anis), 135.7 (ipso), 135.5 (ipso), 135.9 (*o*-Cl), 134.8 (ipso), 132.6 (ipso), 131.4 (β-pyrr), 129.4 (β-pyrr), 128.9 (*m*-Cl), 128.4 (*m*-Cl), 127.9 (*m*-Cl), 125.5 (β-pyrr), 115.7 (*m*-Anis), 113.7 (*m*-Anis), 56.1 (OMe), 55.9 (OMe); **UV-Vis (nm, logε)**: 851 (3.4), 605 (3.8), 468 (4.1); **HRMS (ESI)** *m/z* = 1038.9090, calc. for $C_{46}H_{31}Cl_4N_2O_2Rh^{130}Te$, $[M+Na]^+$: 1038.9102.

$2_{B'}$-H: **^{1}H NMR ($CDCl_3$, 300 K, 500 MHz)**: δ 10.57 (d, $^3J_{HH}$ = 5.2 Hz, 1H, rhodacycle), 10.51 (d, $^3J_{HH}$ = 5.2 Hz, 1H, rhodacycle), 9.68 (d, $^3J_{HH}$ = 5.4 Hz, 1H, tell), 9.28 (d, $^3J_{HH}$ = 5.4 Hz, 1H, tell), 8.72 (d, $^3J_{HH}$ = 5.0 Hz, 1H, pyrr), 8.70 (dd, $^3J_{HH}$ = 4.6 Hz, $^4J_{HH}$ = 2.0 Hz, 1H, pyrr), 8.67 (d, $^3J_{HH}$ = 5.0 Hz, 1H, pyrr), 8.57 (m, 2H, *o*-Anis), 8.46 (m, 1H, *o*-Cl), 8.21 (m, 5H, pyrr, *o*-Anis/Cl), 7.96 (m, 2H, *m*-Anis), 7.84 (m, 2H, *m*-Cl), 7.72 (m, 1H, *o*-Cl), 7.42 (m, 1H, *m*-Cl), 7.37 (m, 2H, *m*-Anis), 7.25 (m, 1H, *m*-Cl), 4.09 (s, 3H, OMe), 4.07 (s, 3H, OMe), 1.80 (brs, 1H, NH); **^{13}C NMR ($CDCl_3$, 300 K, 150 MHz)**: δ 220.1 (d, $^1J_{RhC}$ = 27 Hz, α-rhodacycle), 194.7 ($^1J_{RhC}$ = 30 Hz, α-rhodacycle), 171.6 (α-pyrr), 167.6 (β-rhodacycle), 160.4 (para-Anis), 160.3 (para-Anis), 153.8 (β-rhodacycle), 152.5 (α-tell), 152.2 (α-pyrr), 151.3 (α-tell), 150.2 (α-pyrr), 148.8 (meso), 147.5 (meso), 145.9 (α-pyrr), 144.9 (β-tell), 143.4 (β-tell), 142.9 (meso), 141.0 (para-Cl), 140.3 (β-pyrr), 139.3 (*o*-Cl), 137.4 (ipso), 136.6 (*o*-Anis), 136.3 (ipso), 136.2 (meso), 135.5 (ipso), 135.4 (*o*-Anis/Cl), 135.3 (*o*-Cl), 132.6 (β-pyrr), 130.2 (*m*-Anis), 129.4 (ipso), 128.3 (*m*-Cl), 128.1 (β-pyrr), 126.5 (β-pyrr), 114.4 (*m*-Anis), 113.7 (*m*-Cl), 113.2 (*m*-Cl), 55.8 (2x, OMe); **UV-Vis (nm, logε)**: 857 (3.5), 600 (3.9), 474 (4.3); **HRMS (ESI)** *m/z* = 1038.9085, calc. for $C_{46}H_{31}Cl_4N_2O_2Rh^{130}Te$, $[M+Na]^+$: 1038.9102.

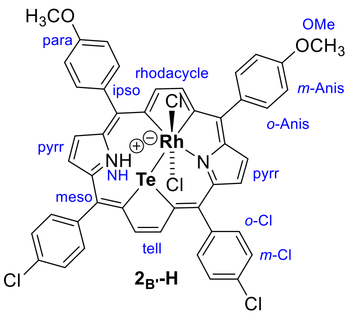

$2_{B'}$-H

Synthesis of 5,10,15,20-tetrakis(4-methoxyphenyl)-di-µ-chloro-21,23-dicarbonyl-21,23-dirhoda-porphyrin, 3_A, from 2_A: 10 mg ($1.0\cdot10^{-5}$ mol) of **2_A** and 40 mg ($1.0\cdot10^{-4}$ mol) of $[Rh(CO)_2Cl]_2$ were dissolved in 15 ml toluene. The solution was refluxed for 10 minutes. The solvent was evaporated and the product was purified by column chromatography on SiO_2 and **3_A** was eluted as a brown band (9% yield).

3_B was synthesized from **2_B** or **$2_{B'}$** in the same way.

Synthesis of 3_A in anaerobic conditions (Schlenk line): 23 mg ($2.4\cdot10^{-5}$ mol) of **1_A** and 9.3 mg ($2.4\cdot10^{-5}$ mol) of $[Rh(CO)_2Cl]_2$ were dissolved in 15 ml degassed toluene in a flask attached to a Schlenk line. The solution was refluxed for 20 minutes. The solvent was evaporated and the product was purified by column chromatography on SiO_2. **3_A** was eluted with CH_2Cl_2 as the main brown band (4% yield).

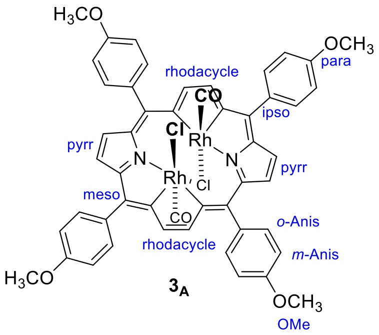

3_A

3_A: **^{1}H NMR ($CDCl_3$, 300 K, 500 MHz):** δ 9.97 (d, $^3J_{RhH}$ = 2.3 Hz, 4H, rhodacycle), 8.84 (s, 4H, pyrr), 8.17 (m, 8H, *o*-Anis), 7.36 (m, 8H, *m*-Anis), 4.11 (s, 12H, OMe); **^{13}C NMR ($CDCl_3$, 300 K, 150 MHz)**: δ 170.5 (d, $^1J_{RhCO}$ = 75 Hz, CO), 166.4 (d, $^1J_{RhC}$ = 29 Hz, α-rhodacycle), 160.2 (para), 157.8 (α-pyrr), 147.6 (d, $^2J_{RhC}$ = 2.9 Hz, β-rhodacycle), 142.1 (meso), 135.4 (β-pyrr), 135.1 (*o*-Anis), 131.7 (ipso), 113.7 (*m*-Anis), 55.8 (OMe); **UV-Vis (nm, logε)**: 845 (3.2), 801 (3.4), 647 (3.7), 487 (4.5), 342 (4.0); **HRMS (ESI)** *m/z* = 1060.9958, calc. for $C_{50}H_{36}N_2O_6Cl_2Rh_2$, $[M+Na]^+$: 1060.9923.

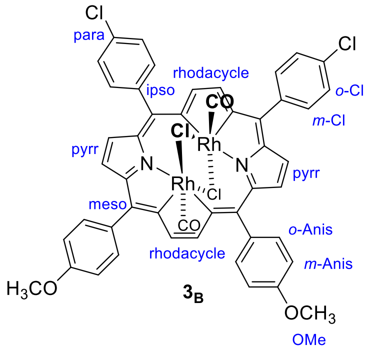

3_B

3_B: **^{1}H NMR ($CDCl_3$, 300 K, 600 MHz):** δ 10.01 (d, $^3J_{RhH}$ = 2.2 Hz, 2H, rhodacycle), 9.91 (d, $^3J_{RhH}$ = 2.2 Hz, 2H, rhodacycle), 8.84 (d, $^3J_{HH}$ = 4.8 Hz, 2H, pyrr), 8.75 (d, $^3J_{HH}$ = 4.8 Hz, 2H, pyrr), 8.18 (m, 8H, *o*-Anis/Cl), 7.82 (m, 4H, *m*-Cl), 7.38 (m, 4H, *m*-Anis), 4.11 (s, 6H, OMe); **^{13}C NMR ($CDCl_3$, 300 K, 150 MHz)**: δ 170.7 (d, $^1J_{RhCO}$ = 75 Hz, CO), 170.6 (d, $^1J_{RhCO}$ = 75 Hz, CO), 167.5 (d, $^1J_{RhC}$ = 29 Hz, α-rhodacycle), 166.4 (d, $^1J_{RhC}$ = 29 Hz, α-rhodacycle), 160.4 (para-Anis), 157.9 (α-pyrr), 157.8 (α-pyrr), 148.8 (d, $^2J_{RhC}$ = 3.3 Hz, β-rhodacycle), 147.7 (d, $^2J_{RhC}$ = 3.3 Hz, β-rhodacycle), 143.3 (meso), 140.5 (meso), 137.7 (para-Cl), 135.8 (β-pyrr), 135.2 (2x, *o*-Anis/Cl), 134.9 (β-pyrr), 134.8 (ipso), 131.7 (ipso), 128.5 (*m*-Cl), 113.8 (*m*-Anis), 55.8 (OMe); **UV-Vis (nm, logε)** 839 (3.6), 791 (3.8), 647 (4.0), 484 (4.8), 341 (4.4); **HRMS (ESI)** *m/z* = 1066.8839, calc. for $C_{48}H_{30}N_2O_4Cl_4Rh_2$, $[M+Na]^+$: 1066.8962.

Synthesis of 5,10,15,20-tetrakis(4-methoxyphenyl)-23-carbonyl-23-chloro-21-oxa-23-rhodaporphyrin, 4_A: 5,10,15,20-tetrakis(4-methoxyphenyl)-21-oxa-23-telluraporphyrin 5_A (10 mg, $1.2\cdot10^{-5}$ mol) and $[Rh(CO)_2Cl]_2$ (4,6 mg, $1.2\cdot10^{-5}$ mol) were dissolved in 15 ml of toluene. Nitrogen was bubbled through the mixture for 15 minutes and the solution was stirred for another 60 minutes. After evaporation of solvent, the product was purified by column chromatography on SiO_2 and 4_A was eluted with CH_2Cl_2 as an orange band (22% yield).

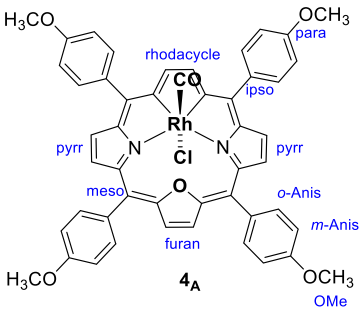

^1H NMR (CD_2Cl_2, 300 K, 500 MHz): δ 10.13 (d, $^3J_{RhH}$ = 2.1 Hz, 2H, rhodacycle), 9.01 (s, 2H, furan), 8.76 (d, $^3J_{HH}$ = 4.9 Hz, 2H, pyrr), 8.72 (d, $^3J_{HH}$ = 4.9 Hz, 2H, pyrr), 8.15 (m, 4H, *o*-Anis), 8.09 (m, 4H, *o*-Anis), 7.39 (m, 4H, *m*-Anis), 7.31 (m, 4H, *m*-Anis), 4.09 (s, 6H, OMe), 4.08 (s, 6H, OMe); **^{13}C NMR ($CDCl_3$, 300 K, 150 MHz)**: δ 174.1 (d, $^1J_{RhCO}$ = 72 Hz, CO), 172.4 (d, $^1J_{RhC}$ = 30 Hz, α-rhodacycle), 167.7 (α-pyrr), 160.1 (para), 159.8 (para), 155.7 (α-furan), 150.5 (α-pyrr), 148.5 (d, $^2J_{RhC}$ = 3.6 Hz, β-rhodacycle), 142.3 (meso), 141.4 (β-pyrr), 135.4 (*o*-Anis), 135.2 (*o*-Anis), 134.6 (ipso), 131.1 (ipso), 130.6 (β-pyrr), 128.2 (β-furan), 119.7 (meso), 113.7 (*m*-Anis), 112.7 (*m*-Anis), 112.6 (*m*-Anis), 55.8 (OMe); **UV-Vis (nm, logε)**: 735 (3.7), 664 (3.2), 578 (3.8), 539 (3.9), 510 (3.8), 453 (4.6), 352 (4.2); **HRMS (ESI)** *m/z* = 888.1454, calc. for $C_{49}H_{36}ClN_2O_6Rh$, $[M+H]^+$: 888.1423.

Synthesis of 5,10,15,20-tetrakis(4-methoxyphenyl)-23,23-dichloro-21-oksa-23-rhodaporphyrin, 4_A-H: 5,10,15,20-tetrakis(4-methoxyphenyl)-21-carbonyl-21-chloro-21-rhoda-23-telluraporphyrin 2_A or **2_A-H** (10 mg, $1.0\cdot10^{-5}$ mol) was dissolved in $CDCl_3$ (0.6 ml) in NMR tube, 1 µl of *tert*-butyl hydroperoxide (5.0–6.0 M in decane, $1.0\cdot10^{-5}$ mol) was added. After three days the product was purified on a TLC plate developed with CH_2Cl_2. **4_A-H** was the second brown band (5% yield). **4_A-H** can be also obtained from **4_A** by treating with HCl(g) but the process is very slow and not efficient.

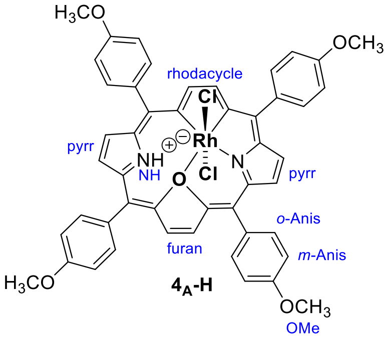

^1H NMR ($CDCl_3$, 300 K, 500 MHz): δ 10.64 (dd, $^3J_{RhH}$ = 1.5 Hz, $^3J_{HH}$ = 4.9 Hz, 1H, rhodacycle), 10.29 (dd, $^3J_{RhH}$ = 1.5 Hz, $^3J_{HH}$ = 4.9 Hz, 1H, rhodacycle), 9.26 (d, $^3J_{HH}$ = 4.7 Hz, 1H, furan), 9.07 (d, $^3J_{HH}$ = 4.7 Hz, 1H, furan), 8.68 (dd, $^4J_{HH}$ = 1.2 Hz, $^3J_{HH}$ = 4.6 Hz, 1H, pyrr), 8.65 (d, $^3J_{HH}$ = 4.9 Hz, 1H, pyrr), 8.62 (d, $^3J_{HH}$ = 4.9 Hz, 1H, pyrr), 8.58 (dd, $^4J_{HH}$ = 1.2 Hz, $^3J_{HH}$ = 4.6 Hz, 1H, pyrr), 8.28 (m, 4H, *o*-Anis), 8.22 (m, 2H, *o*-Anis), 8.12 (m, 2H, *o*-Anis), 7.40 (m, 6H, *m*-Anis), 7.28 (m, 2H, *m*-Anis), 4.11 (br, 12H, OMe), -0.44 (brs, 1H, NH); **UV-Vis (nm, logε)**: 759 (3.6), 437 (4.4); **HRMS (ESI)** *m/z* = 917.1081, calc. for $C_{48}H_{37}Cl_2N_2O_5Rh$, $[M+Na]^+$: 917.1027.

Synthesis of 10,15-bis(4-methoxyphenyl)-5,20-bis(4-methylphenyl)-21-oxa-23-telluraporphyrin, 5_B: $BF_3\cdot Et_2O$ (40 µl, 0.32 mmol), was added to 2,5-bis[(4-methylphenyl)hydroxymethyl]furan (0.28 g, 0.9 mmol) and 2,5-bis(2-pyrrolyl(4-methoxyphenyl)methyl)tellurophene (0.50 g, 0.9 mmol) in 500 ml of degassed CH_2Cl_2 and the reaction mixture was stirred for 45 min in the dark at ambient temperature. *p*-Chloranil (1.0 g, 4.1 mmol) was added and the resulting mixture was refluxed for another 30 min. The reaction mixture was filtered through deactivated Al_2O_3, Brockmann Grade III. The solvent was evaporated and the mixture was purified by column chromatography on SiO_2 and 5_B was eluted as a second orange band with CH_2Cl_2 (8.5% yield).

Synthesis of 5,10,15,20-tetrakis(4-methoxyphenyl)-21-oxa-23-telluraporphyrin, **5_A**, was performed from equimolar mixture of 2,5-bis[(4-methoxyphenyl)hydroxymethyl]furan and 2,5-bis(2-pyrrolyl(4-methoxyphenyl)methyl)tellurophene according to the same procedure as for **5_B**. The side product of the synthesis of **5_A**, was 5,10,15,20-tetrakis(4-methoxyphenyl)-21,23-ditelluraporphyrin, **1_A**. Identical *meso*-substitution of both products, **1_A** and **5_A**, rendered the chromatographic separation of **1_A** and **5_A** practically impossible. Thus, to fully characterize 21-oxa-23-telluraporphyrin, **5_B** with differentiated *meso*-substituents was obtained, allowing for purification on required scale and crystallization. Very close similarity of ^1H NMR characteristics of **5_A** and **5_B** is shown in Figure S31. Contamination of **5_A** with **1_A** in further synthesis of **4_A** did not interfere with the course of the reaction.

5_B: **^{1}H NMR (CDCl$_3$, 300 K, 500 MHz):** δ 10.69 (s, 2H, tell), 9.12 (s, 2H, furan), 8,56 (d, $^3J_{HH}$ = 4.4 Hz, 2H, pyrr), 8.45 (d, $^3J_{HH}$ = 4.4 Hz, 2H, pyrr), 8.19 (m, 4H, *o*-Anis), 8.07 (m, 4H, *o*-Tol), 7.54 (m, 4H, *m*-Tol), 7.38 (m, 4H, *m*-Anis), 4.09 (s, 6H, OMe), 2.71 (s, 6H, Me); **^{13}C NMR (CDCl$_3$, 300 K, 125 MHz)**: δ 164.6 (α-tell), 161.6 (α-pyrr), 159.5 (para-Anis), 154.7 (α-furan), 150.4 (α-pyrr), 143.7 (β-tell), 142.6 (meso), 140.2 (ipso-Tol), 138.0 (para-Tol), 136.4 (β-pyrr), 135.6 (*o*-Anis), 134.2 (*o*-Tol), 132.7 (ipso-Anis), 131.5 (β-pyrr), 129.1 (β-furan), 127.6 (*m*-Tol), 120.9 (meso), 113.6 (*m*-Anis), 55.6 (OMe), 21.6 (Me); **UV-Vis (nm, logε)**: 723 (3.9), 446 (5.1); **HRMS (ESI)** *m/z* = 819.1839 calc. for $C_{48}H_{36}N_2O_3{}^{130}Te$, [M+H]+: 819.1861.

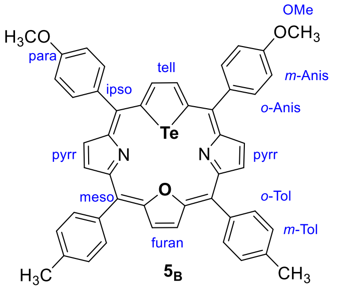

5_B

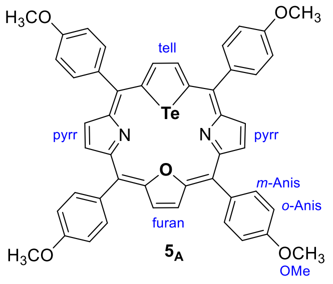

5_A

5_A: **^{1}H NMR (CDCl$_3$, 300 K, 500 MHz):** δ 10.69 (s, 2H, tell), 9.12 (s, 2H, furan), 8,57 (d, $^3J_{HH}$ = 4.4 Hz, 2H, pyrr), 8.47 (d, $^3J_{HH}$ = 4.4 Hz, 2H, pyrr), 8.20 (m, 4H, *o*-Anis), 8.11 (m, 4H, *o*-Anis), 7.39 (m, 4H, *m*-Anis), 7.28 (m, 4H, *m*-Anis), 4.10 (s, 6H, OMe), 4.09 (s, 6H, OMe).

Synthesis of reactive intermediate, rhodium(I) side-on complex with 5,10,15,20-tetrakis(4-methoxyphenyl)-21,23-ditelluraporphyrin, 8: 10 mg (1.0·10^{-5} mol) of **1_A** was dissolved in CDCl$_3$ (0.6 ml) in NMR tube, 4 mg (1.0·10^{-5} mol) of [Rh(CO)$_2$Cl]$_2$ was added and the tube was placed quickly in NMR spectrometer probe cooled to 280 K. **8** was formed immediately. Above 280 K **8** converted to **2_A**.

^{1}H NMR (CD$_2$Cl$_2$, <u>280 K</u>, 600 MHz): δ 8.48 (s, 2H, tell-2/3), 8.11 (m, 4H, *o*-Anis), 8.00 (d, $^3J_{HH}$ = 4.6 Hz, 2H, pyrr), 7.62 (m, 4H, *o*-Anis), 7.48 (d, $^3J_{HH}$ = 4.6 Hz, 2H, pyrr), 7.23 (m, 4H, *m*-Anis), 7.20 (m, 4H, *m*-Anis), 6.58 (br. s, 2H, tell-12/13), 4.00 (s, 6H, OMe), 3.96 (s, 6H, OMe); **^{1}H NMR (CD$_2$Cl$_2$, <u>180 K</u>, 600 MHz):** δ 8.59 (d, $^3J_{HH}$ = 6.0 Hz, 1H, tell-2/3), 8.48 (m, 2H, tell-2/3, *o*-Anis), 8.09 (m, 2H, *o*-Anis), 8.04 (m, 1H, pyrr), 7.98 (m, 1H, pyrr), 7.94 (m, 1H, *o*-Anis), 7.86 (m, 1H, *o*-Anis), 7.80 (m, 1H, *o*-Anis), 7.61 (m, 1H, pyrr), 7.52 (m, 1H, *o*-Anis), 7.47 (m, 1H, *o*-Anis), 7.40 (m, 1H, pyrr), 7.29 – 7.19 (m, 8H, *m*-Anis), 7.00 (br. s, 1H, tell-12/13), 5.87 (br .s, 1H, tell-12/13), 4.00 (s, 3H, OMe), 3.96 (s, 3H, OMe), 3.94 (bs, 6H, OMe) – signals are broad.

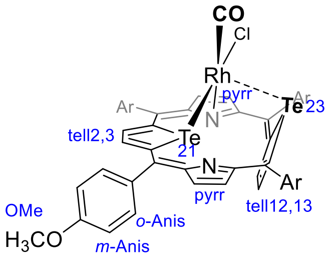

8

Synthesis of intermediate complex, 9: 10 mg (1.0·10^{-5} mol) of **1_A** was dissolved in CDCl$_3$ (0.6 ml) in NMR tube, 4 mg (1.0·10^{-5} mol) of [Rh(CO)$_2$Cl]$_2$ was added. After a few minutes at room temperature **9** could be observed in ^{1}H NMR spectrum, although contaminated with the final reaction product, **2_A**. When conducting the reaction in boiling dichloromethane for 30 minutes, **9** was formed along with **2_A**.

^{1}H NMR (CDCl$_3$, 300 K, 500 MHz): δ 8.56 (dd, $^3J_{HH}$ = 10.8 Hz, $^3J_{RhH}$ = 1.7 Hz, 1H, rhodatelluracycle-2), 8.02 (m, 2H, *o*-Anis), 7.89 (d, $^3J_{HH}$ = 10.8 Hz, 1H, rhodatelluracycle-3), 7.75 (d, $^3J_{HH}$ = 4.9 Hz, 1H, pyrr), 7.71–7.69 (m, 3H, pyrr, *o*-Anis), 7.65 (m, 2H, *o*-Anis), 7.31 (m, 2H, *o*-Anis), 7.20 (d, $^3J_{HH}$ = 4.6 Hz, 1H, pyrr), 7.15–7.11 (m, 4H, *m*-Anis), 7.09 (m, 2H, *m*-Anis), 6.94 (m, 2H, *m*-Anis), 6.89 (d, $^3J_{HH}$ = 4.7 Hz, 1H, pyrr), 6.84 (d, $^3J_{HH}$ = 7.9 Hz, 1H, tell-12/13), 6.72 (d, $^3J_{HH}$ = 7.9 Hz, 1H, tell-12/13), 3.97 (s, 3H, OMe), 3.96 (s, 3H, OMe), 3.92 (s, 3H, OMe), 3.85 (s, 3H, OMe).

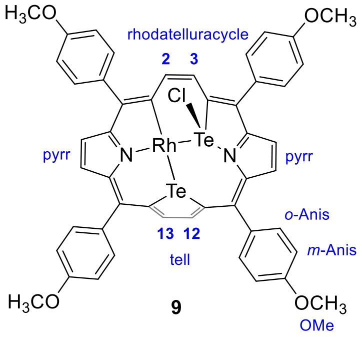

9

Synthesis of rhodium(I) side-on complex with 5,10,15,20-tetrakis(4-methoxyphenyl)-21-carbonyl-21-chloro-21-rhoda-23-telluraporphyrin, 10: Solution of **2_A** (5 mg, $5.0\cdot10^{-6}$ mol) and $[Rh(CO)_2Cl]_2$ (1.9 mg, $5.0\cdot10^{-6}$ mol) in $CDCl_3$ (0.6 ml) was shaking in NMR tube for five minutes. Formation of **10** was be observed in ^{1}H NMR spectrum. At room temperature **10** converted to a mixture of **2_A** and **2_A-H** in seven days. **10** converted to **3_A** after 10 min in boiling toluene.

^{1}H NMR ($CDCl_3$, 300 K, 500 MHz): δ 10.45 (d, $^3J_{HH}$ = 6.1 Hz, 1H, rhodacycle), 9.76 (d, $^3J_{HH}$ = 6.1 Hz, 1H, rhodacycle), 9.39 (d, $^3J_{HH}$ = 5.5 Hz, 1H, tell), 9.20 (d, $^3J_{HH}$ = 5.5 Hz, 1H, tell), 8.64 (d, $^3J_{HH}$ = 5.1 Hz, 1H, pyrr), 8.52 (m, 2H, *o*-Anis), 8.49 (d, $^3J_{HH}$ = 5.1 Hz, 1H, pyrr), 8.23 (d, $^3J_{HH}$ = 4.5 Hz, 1H, pyrr), 8.05 (m, 2H, *o*-Anis), 8.02 (d, $^3J_{HH}$ = 4.5 Hz, 1H, pyrr), 7.97 (m, 2H, *o*-Anis), 7.39–7.20 (m, 10 H, *o*/*m*-Anis), 4.10 (s, 3H OMe), 4.06 (s, 3H OMe), 4.03 (s, 3H OMe), 3.99 (s, 3H OMe); **UV-Vis (nm)**: 1040, 658, 598, 488, 438.

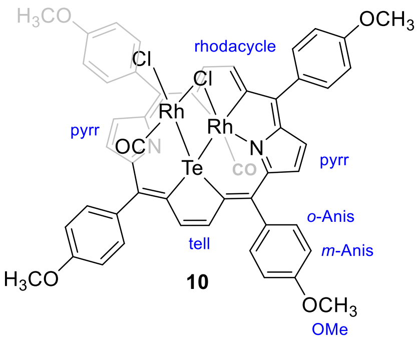

10

Figures

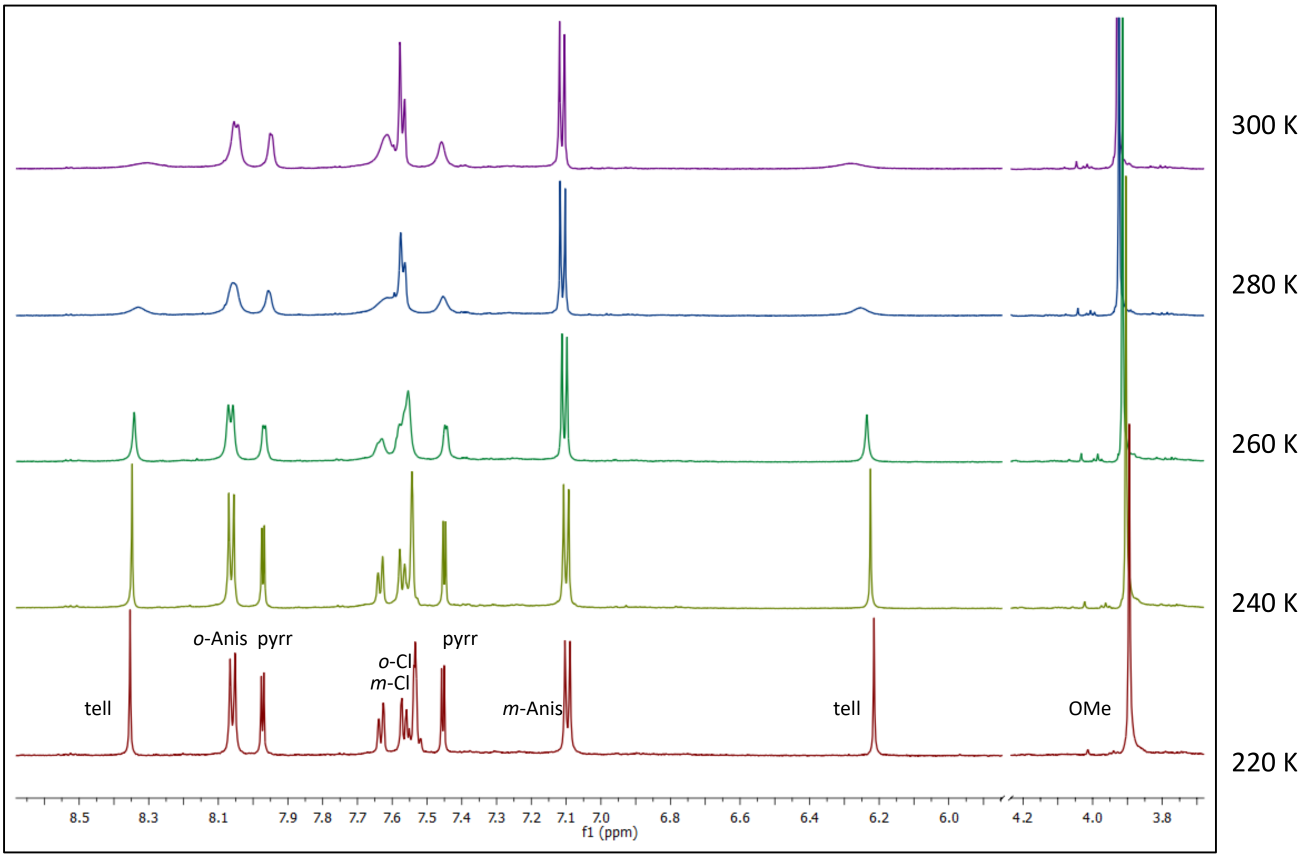

Figure S1. Variable temperature ^{1}H NMR spectra of **1_B**; 600 MHz, CD_2Cl_2, 220–300 K.

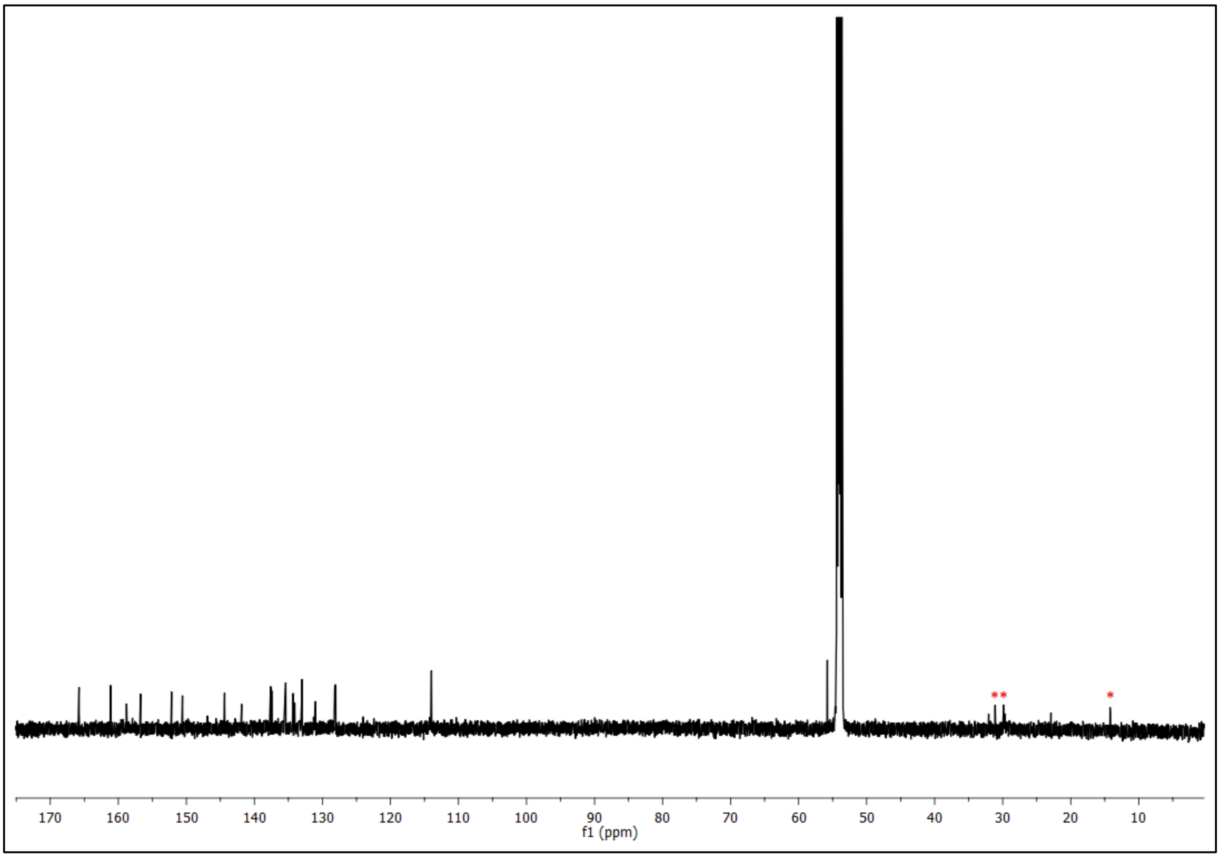

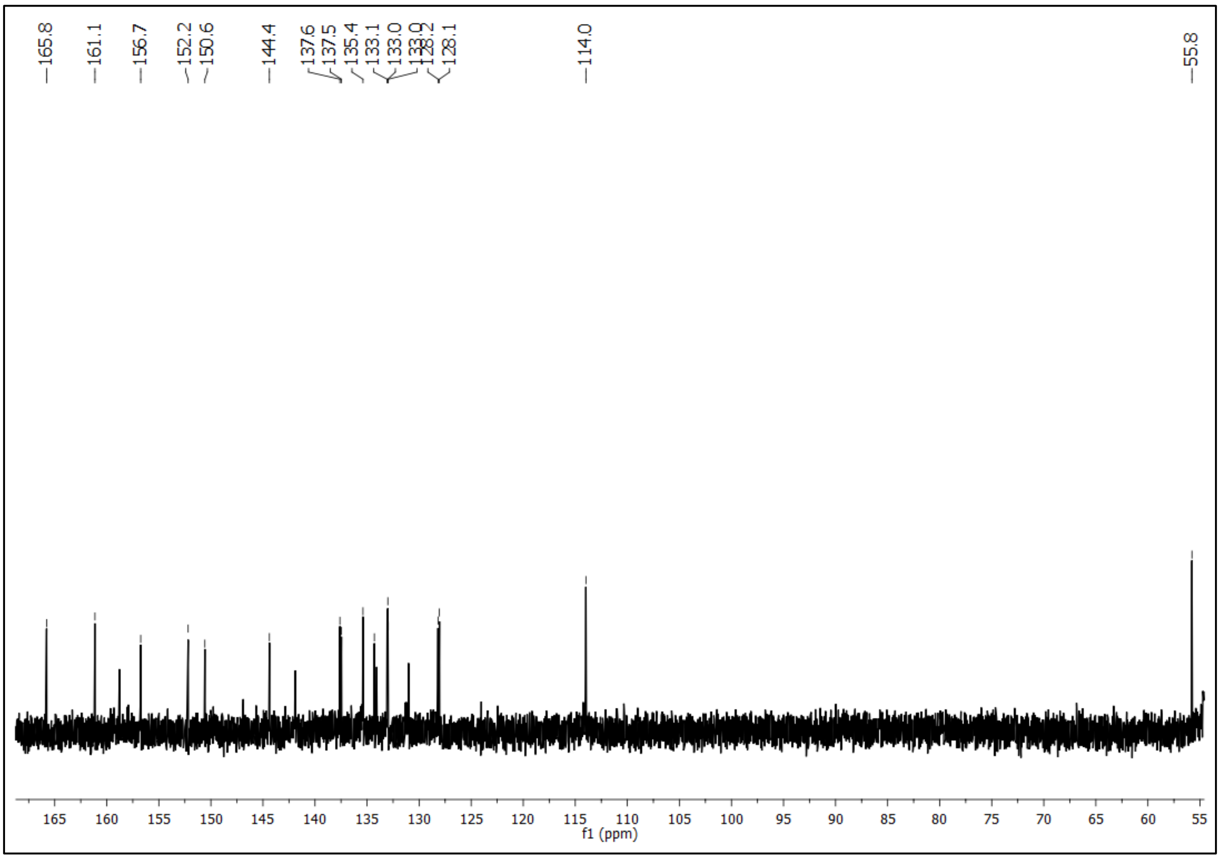

Figure S2. ^{13}C NMR spectrum of $\mathbf{1_B}$; 150 MHz, CD_2Cl_2, 220 K (top: the whole spectral range, bottom: the most informative region; .* = impurities).

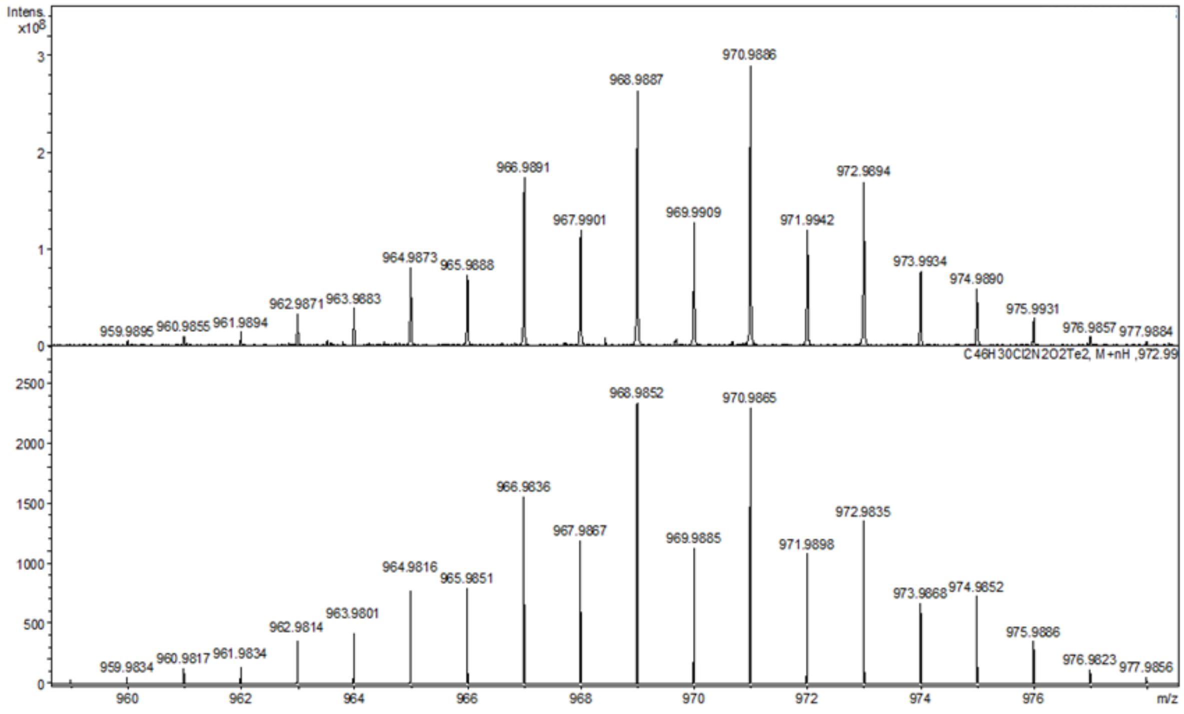

Figure S3. HRMS ESI (+MS) spectra of $\mathbf{1_B}$: measured (top) and simulated (bottom) calc. for $C_{46}H_{30}Cl_2N_2O_2Te_2$, $[M+H]^+$.

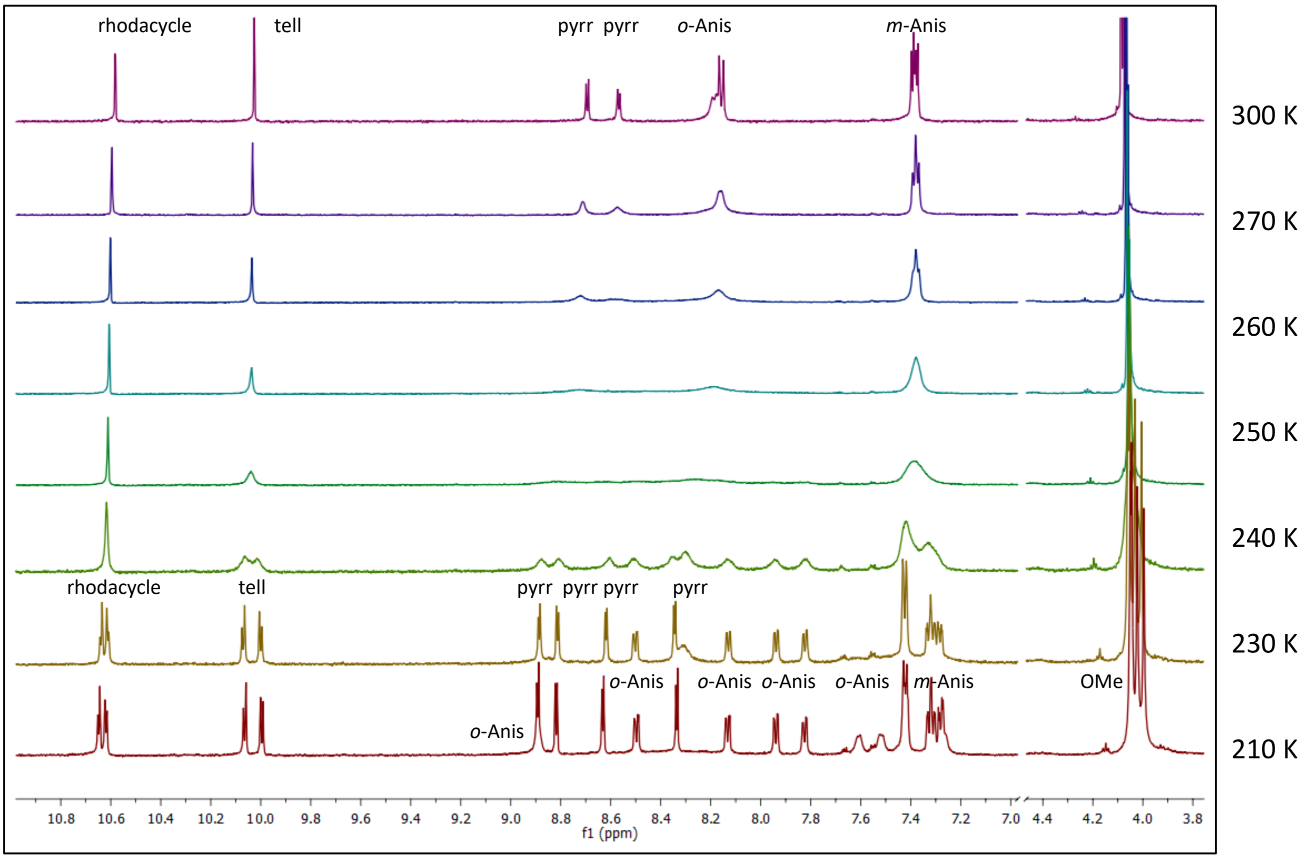

Figure S4. Variable temperature ^{1}H NMR spectra of $\mathbf{2_A}$; 600 MHz, CD_2Cl_2, 190–300 K.

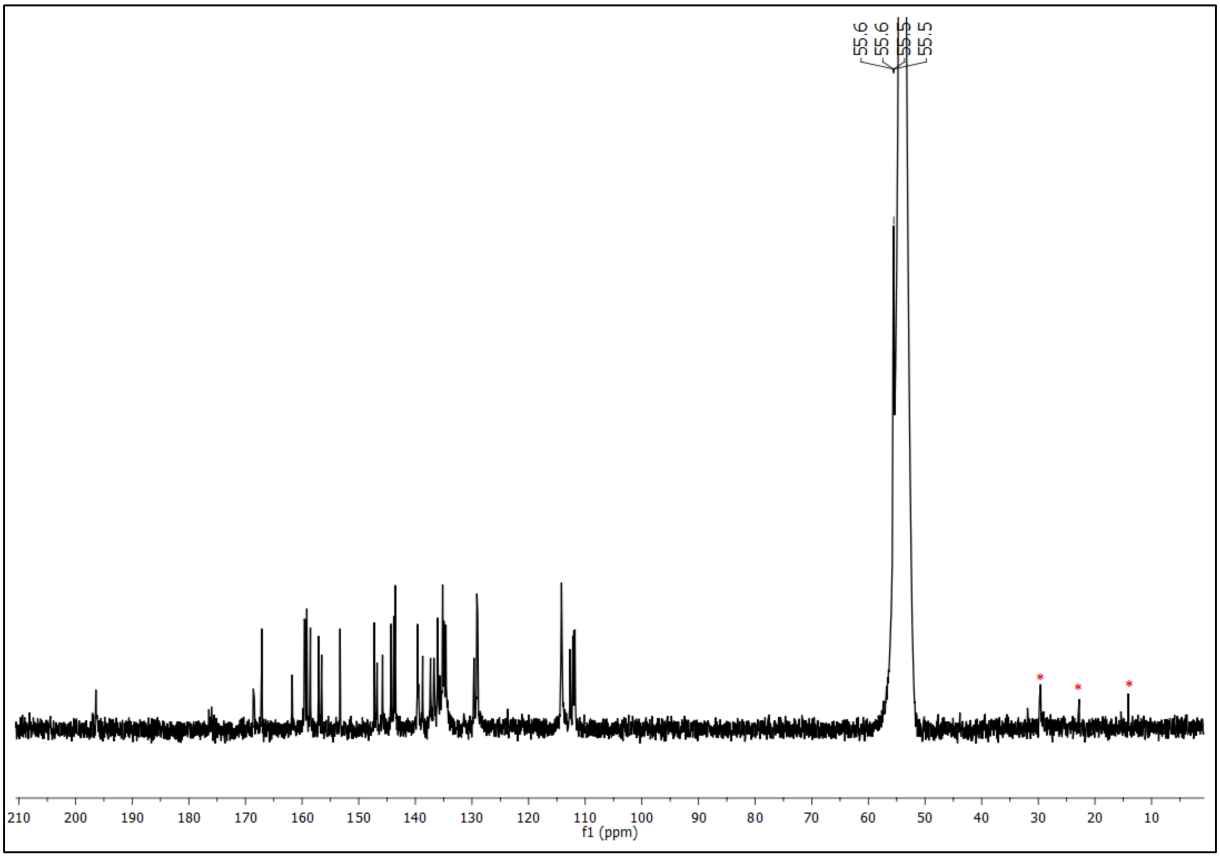

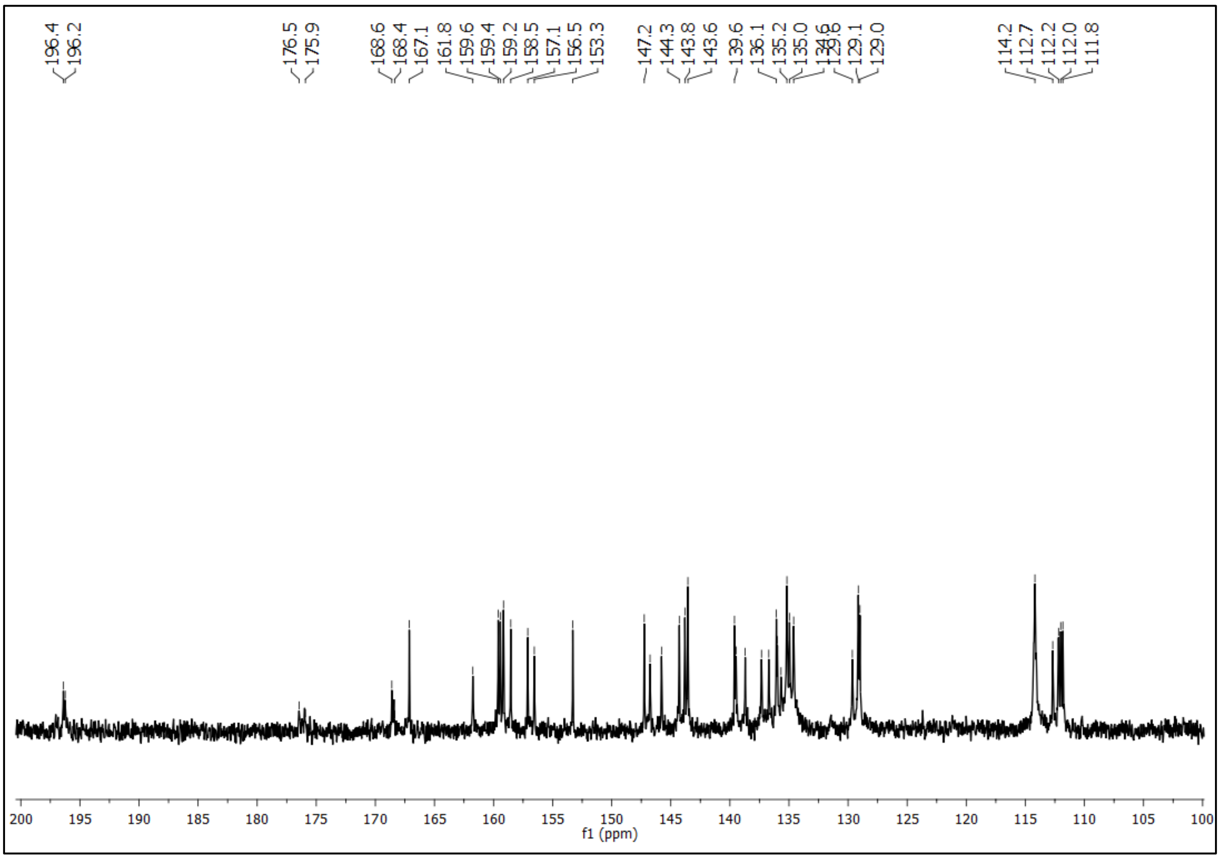

Figure S5. ^{13}C NMR spectrum of $\mathbf{2_A}$; 150 MHz, CD_2Cl_2, 190 K (top: the whole spectral range, bottom: the most informative region; .* = impurities).

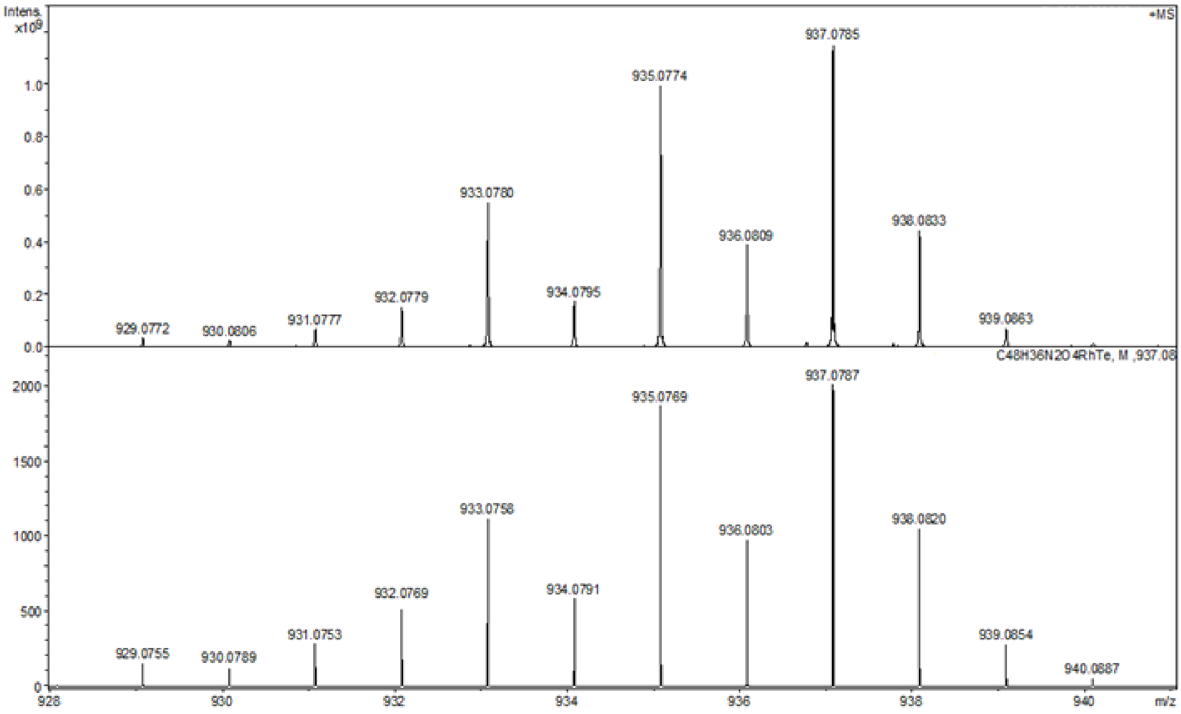

Figure S6. HRMS ESI (+MS) spectra of $\mathbf{2_A}$: measured (top) and simulated (bottom) calc. for $C_{48}H_{36}N_2O_4RhTe$, $[M\text{-}Cl\text{-}CO]^+$.

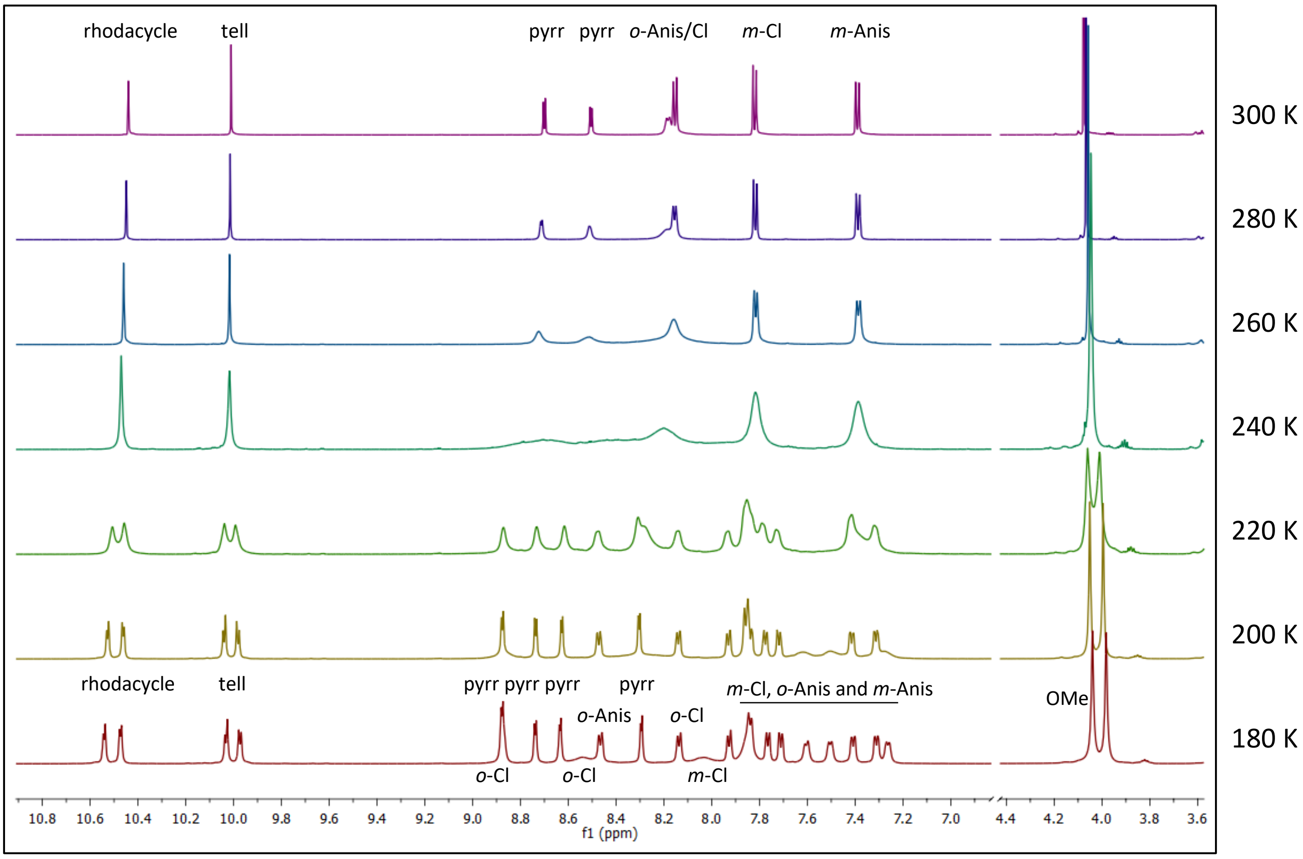

Figure S7. Variable temperature ^{1}H NMR spectra of $\mathbf{2_B}$; 600 MHz, CD_2Cl_2, 180–300 K.

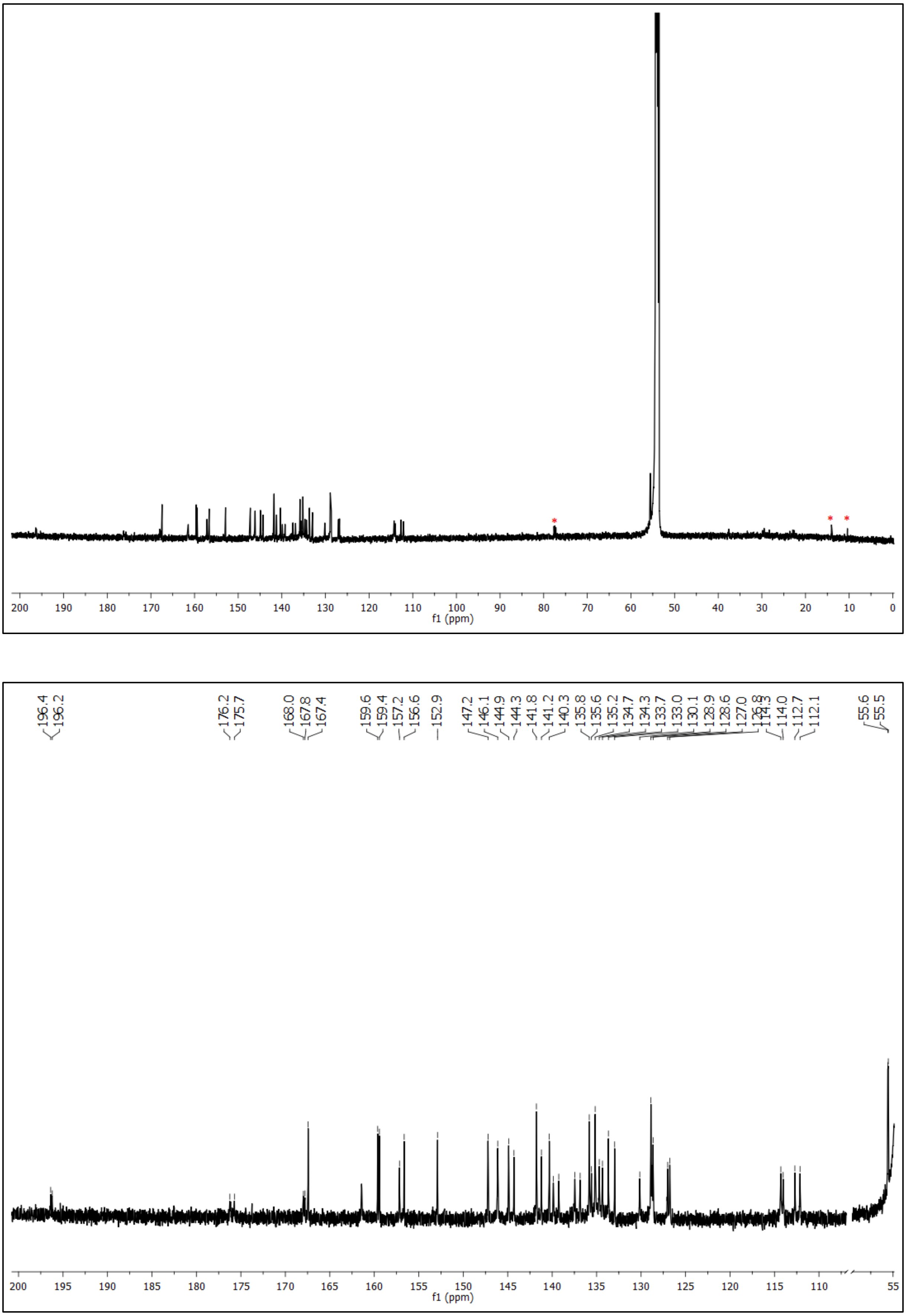

Figure S8. ^{13}C NMR spectrum of $\mathbf{2_B}$; 150 MHz, CD_2Cl_2, 180 K (top: the whole spectral range, bottom: the most informative region; .* = impurities).

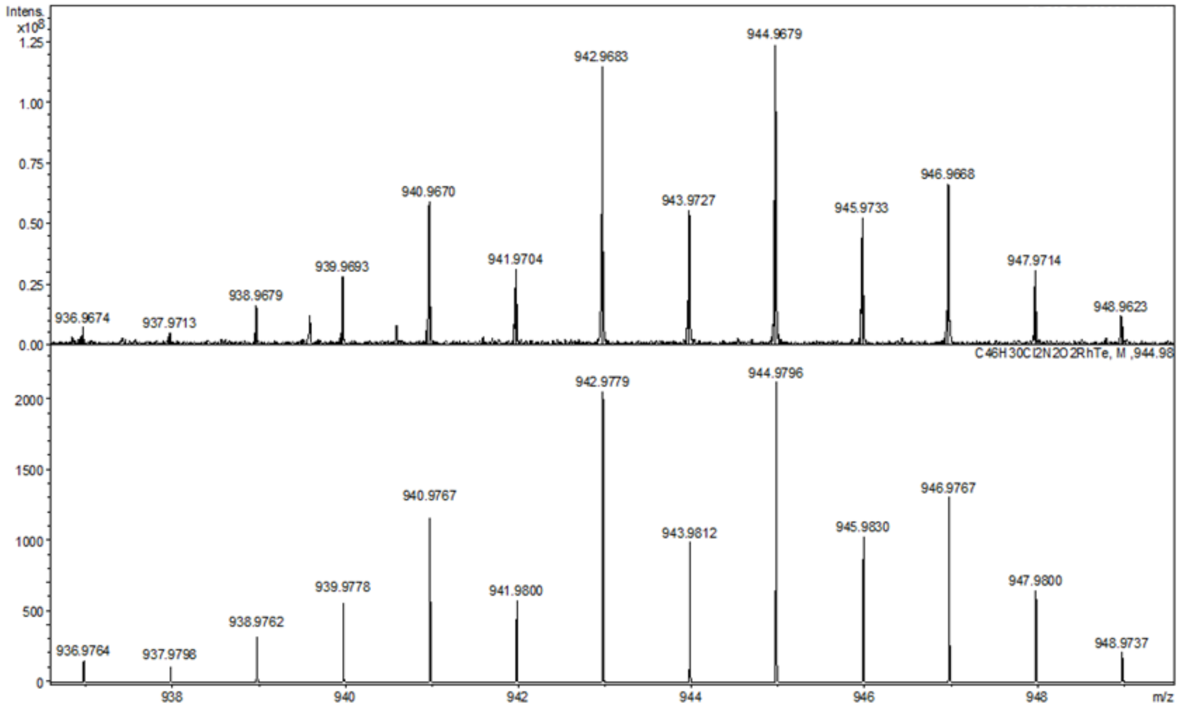

Figure S9. HRMS ESI (+MS) spectra of $\mathbf{2_B}$: measured (top) and simulated (bottom) calc. for $C_{46}H_{30}N_2O_2Cl_2RhTe$, $[M\text{-}Cl\text{-}CO]^+$.

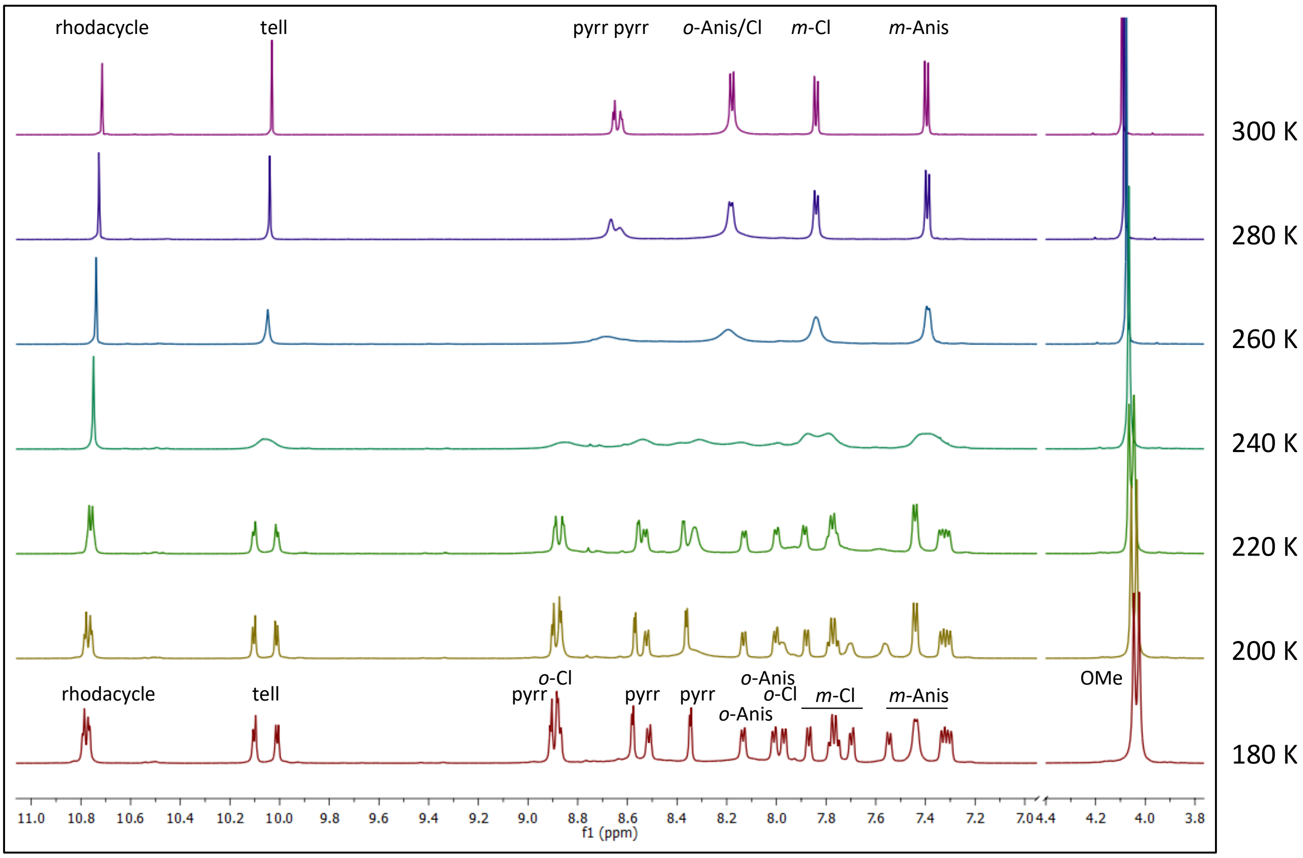

Figure S10. Variable temperature ^1H NMR spectra of $\mathbf{2_{B'}}$; 600 MHz, CD_2Cl_2, 180–300 K.

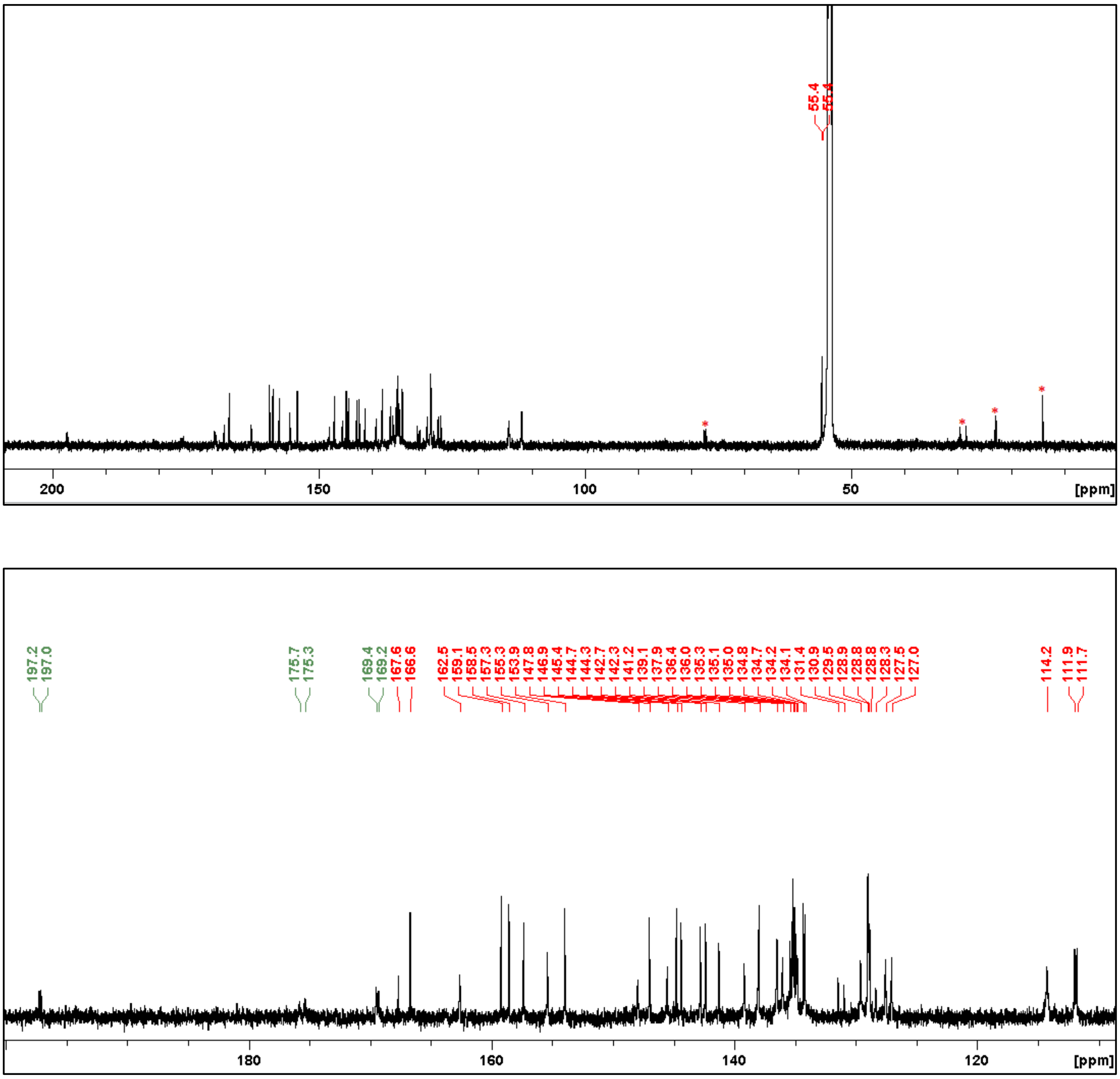

Figure S11. ^{13}C NMR spectrum of **2**$_{B'}$; 150 MHz, CD_2Cl_2, 180 K (top: the whole spectral range, bottom: the most informative region; .* = impurities).

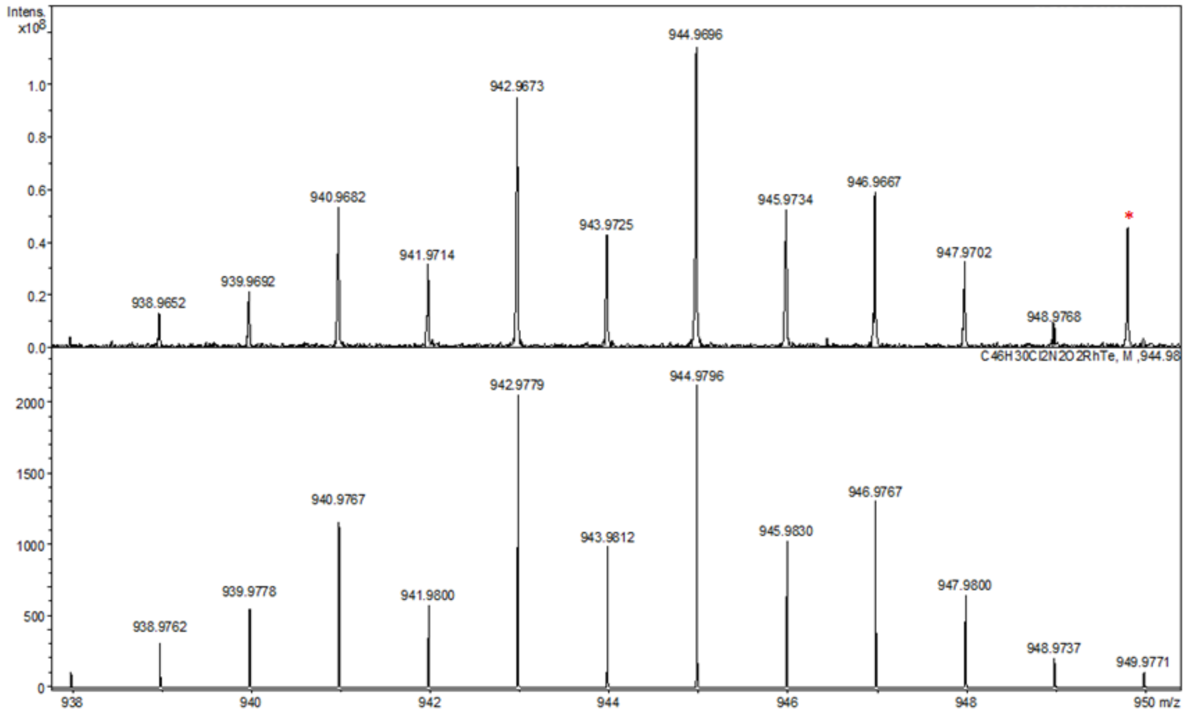

Figure S12. HRMS ESI (+MS) spectra of **2**$_{B'}$: measured (top) and simulated (bottom) calc. for $C_{46}H_{30}N_2O_2Cl_2RhTe$, [M-Cl-CO]

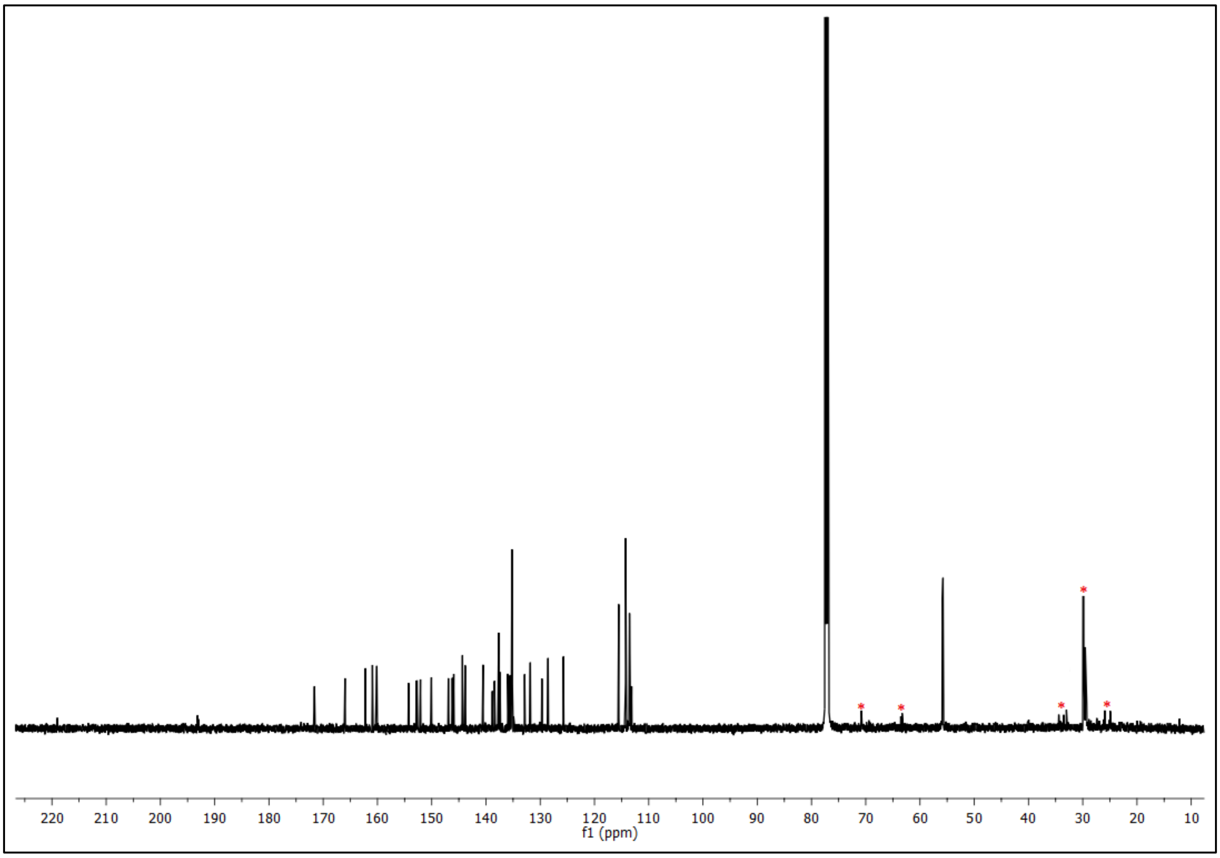

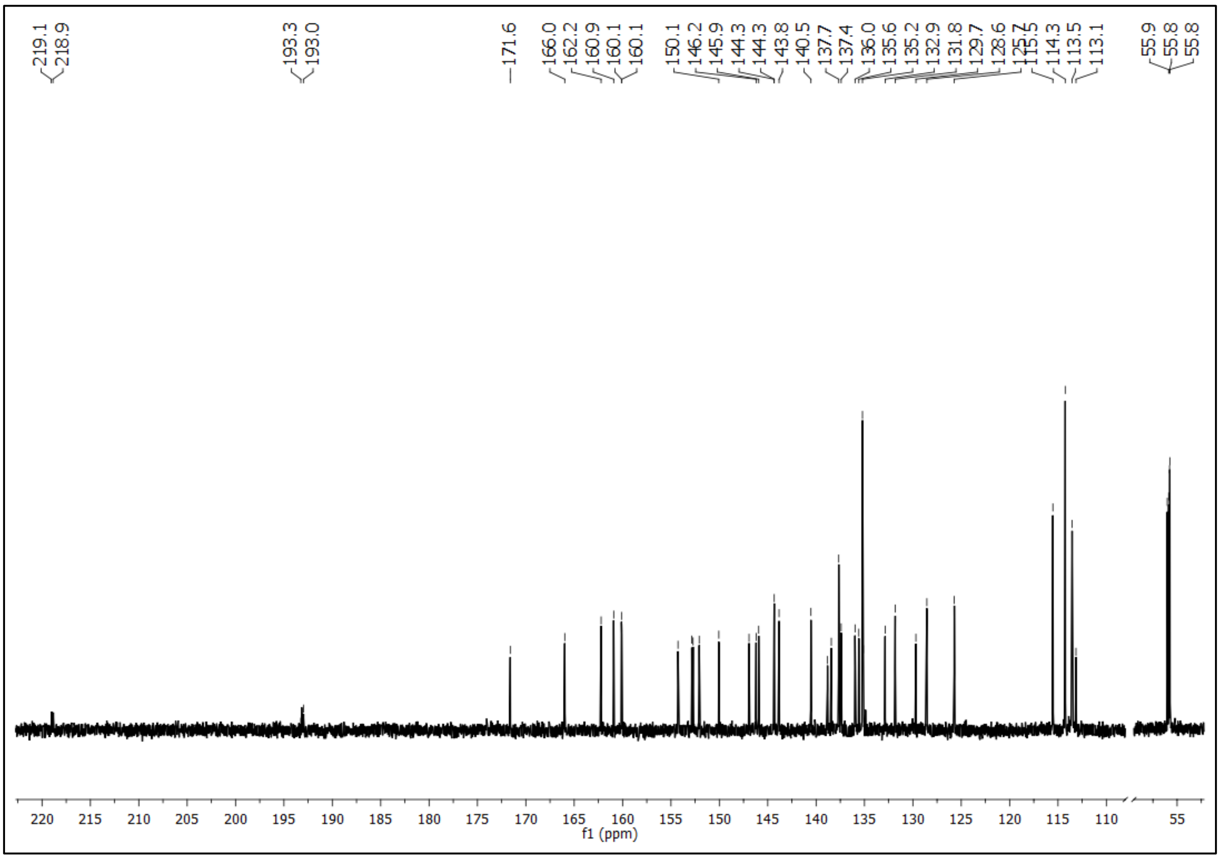

Figure S13. ^{13}C NMR spectrum of **2_A-H**; 150 MHz, $CDCl_3$, 300 K (top: the whole spectral range, bottom: the most informative region; .* = impurities).

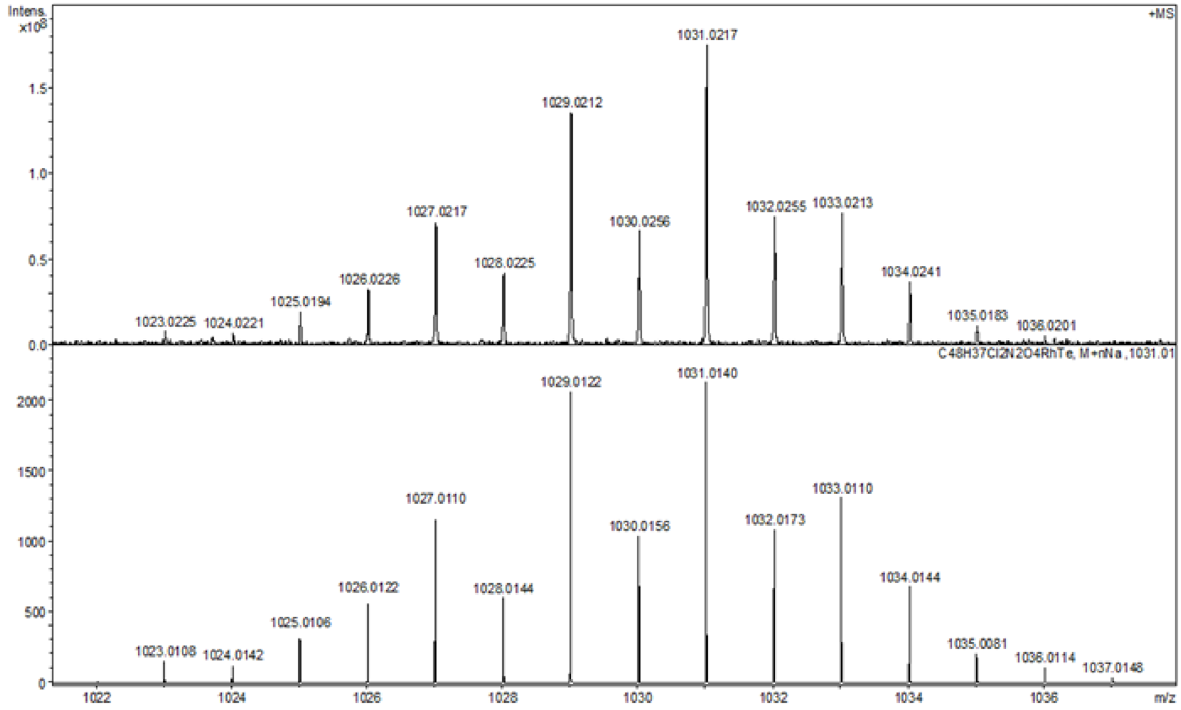

Figure S14. HRMS ESI (+MS) spectra of **2_A-H**: measured (top) and simulated (bottom) calc. for $C_{48}H_{37}Cl_2N_2O_4RhTe$, $[M+Na]^+$.

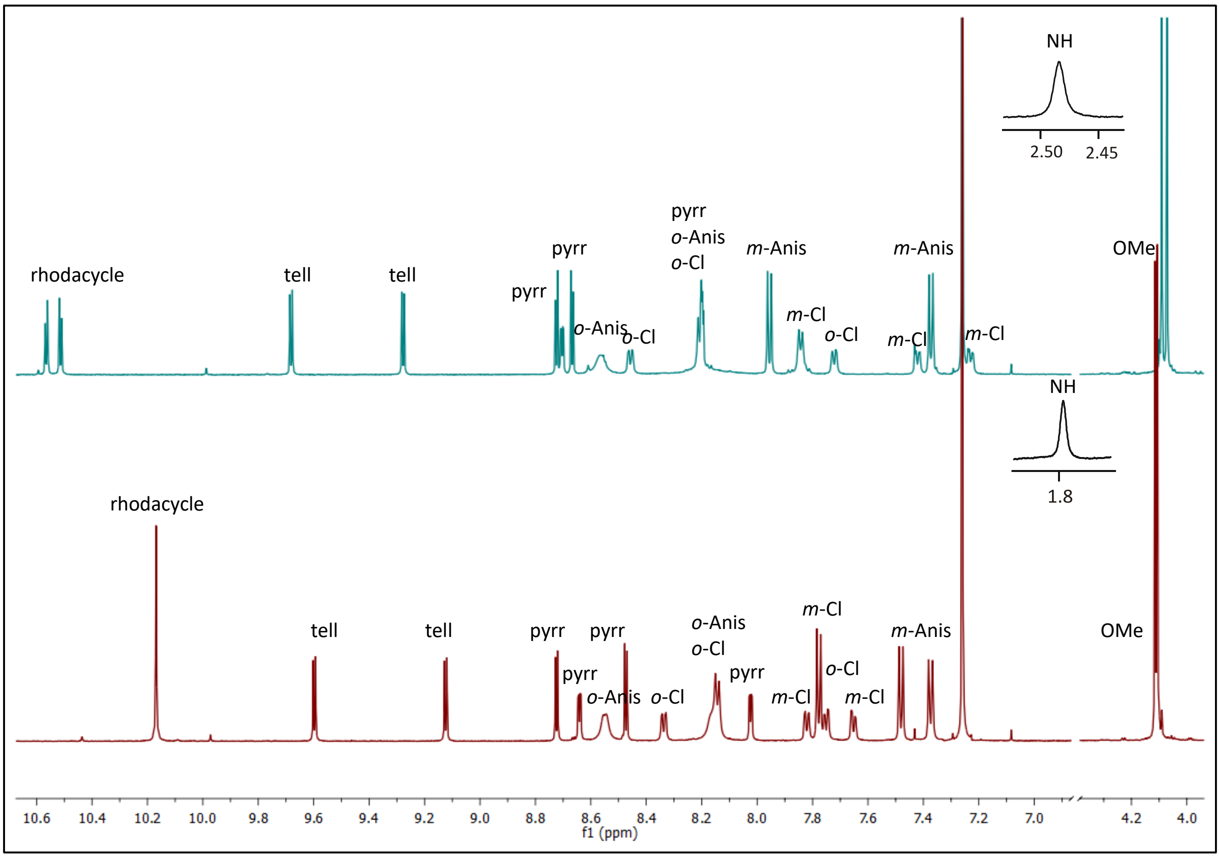

Figure S15. ^1H NMR spectra of **2_B-H** (bottom) and **$2_{B'}$-H** (top); 500 MHz, $CDCl_3$, 300 K.

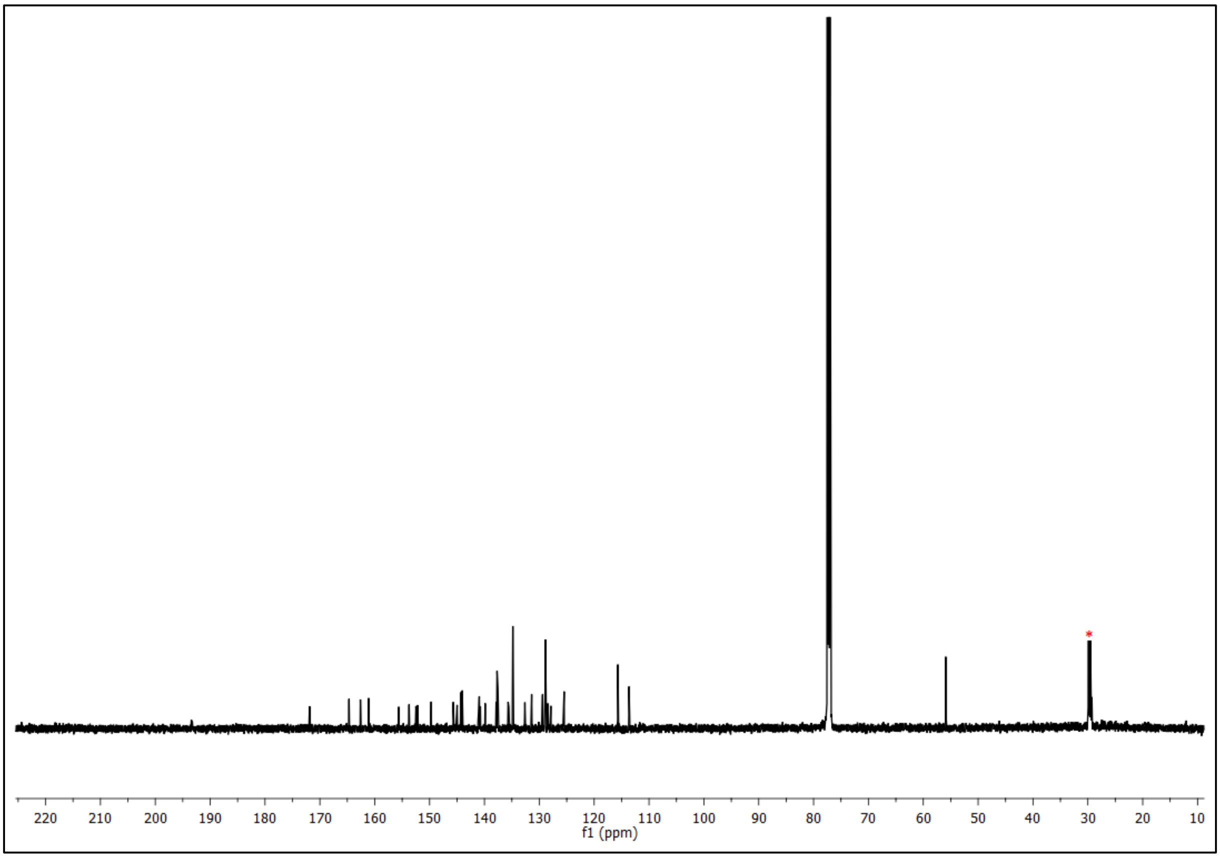

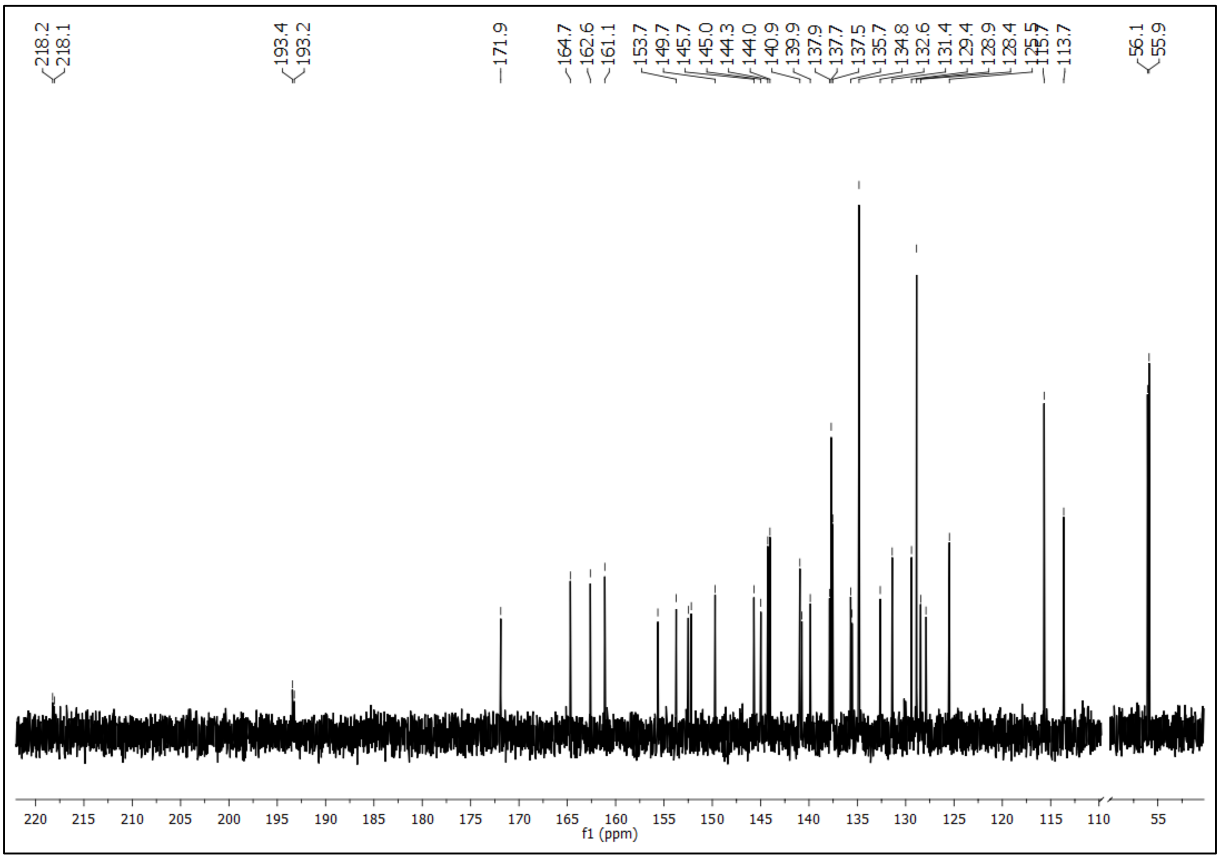

Figure S16. ^{13}C NMR spectrum of **2_B-H**; 150 MHz, $CDCl_3$, 300 K (top: the whole spectral range, bottom: the most informative region; .* = impurities).

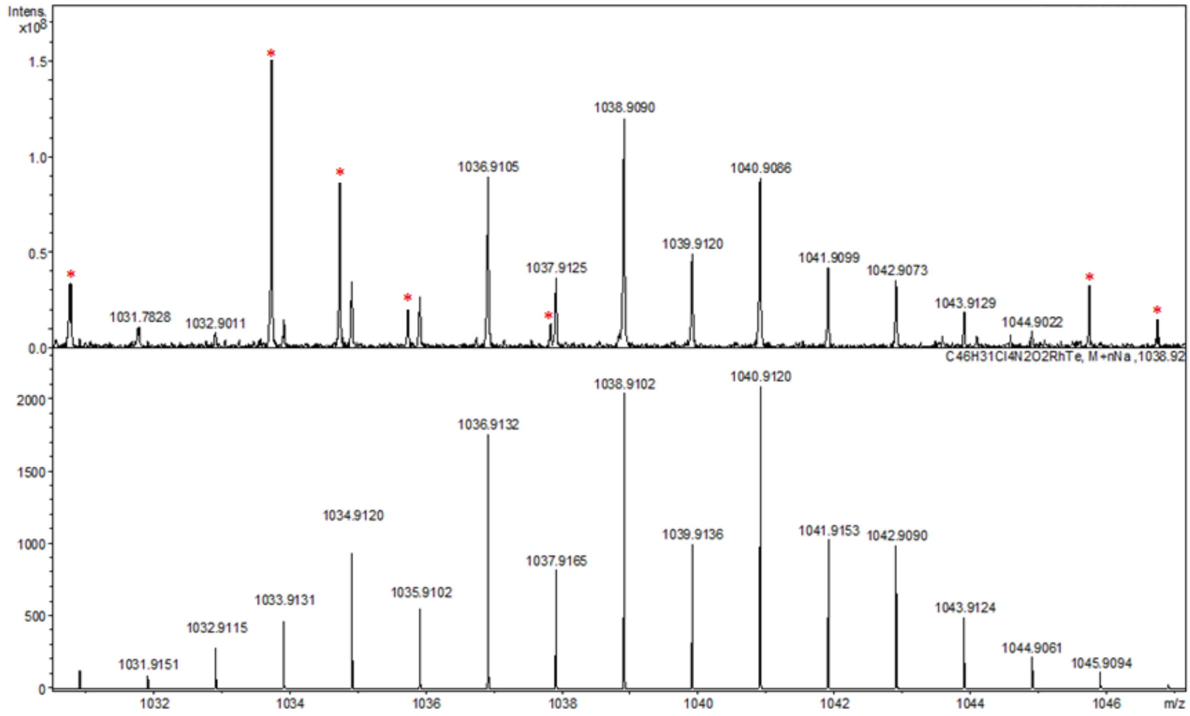

Figure S17. HRMS ESI (+MS) spectra of **2_B-H**: measured (top) and simulated (bottom) calc. for $C_{46}H_{31}Cl_4N_2O_2RhTe$, $[M+Na]^+$.

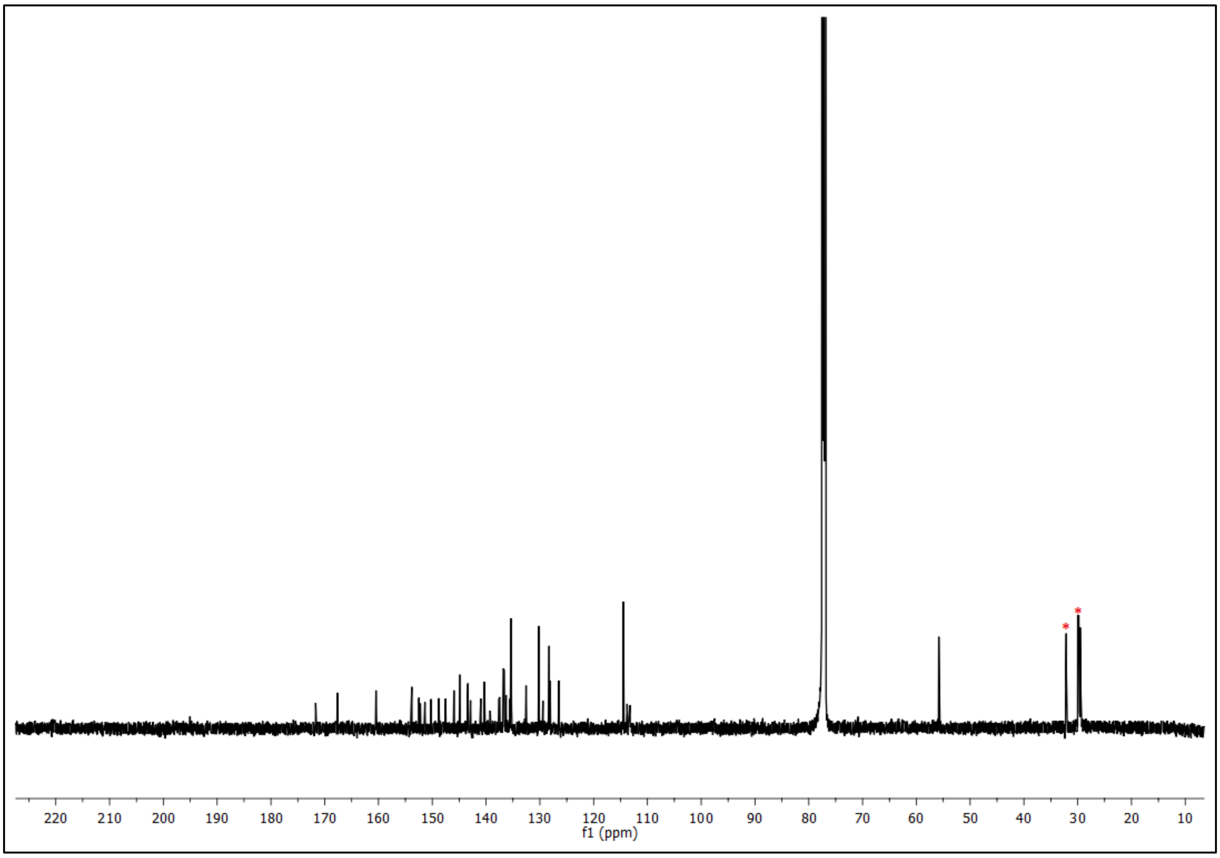

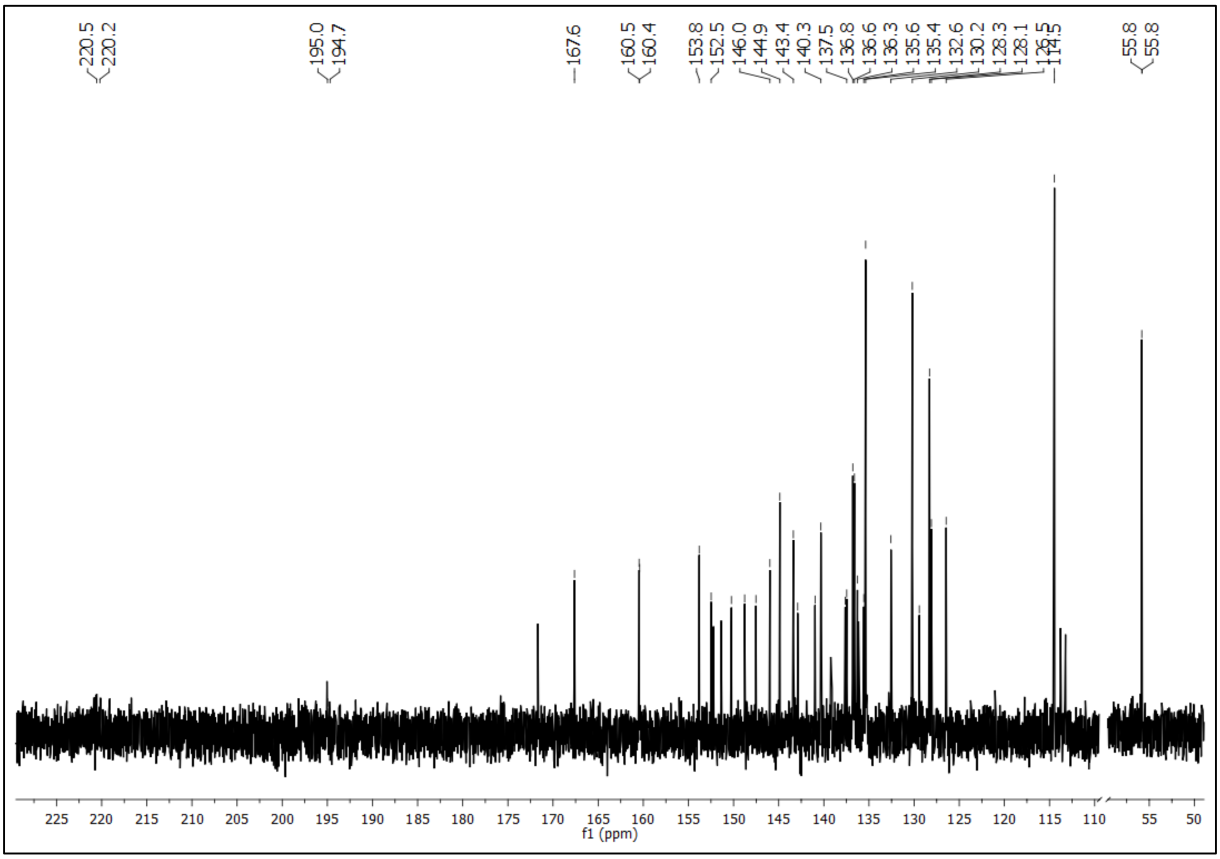

Figure S18. ^{13}C NMR spectrum of $\mathbf{2_{B'}}$**-H**; 150 MHz, $CDCl_3$, 300 K (top: the whole spectral range, bottom: the most informative region; .* = impurities).

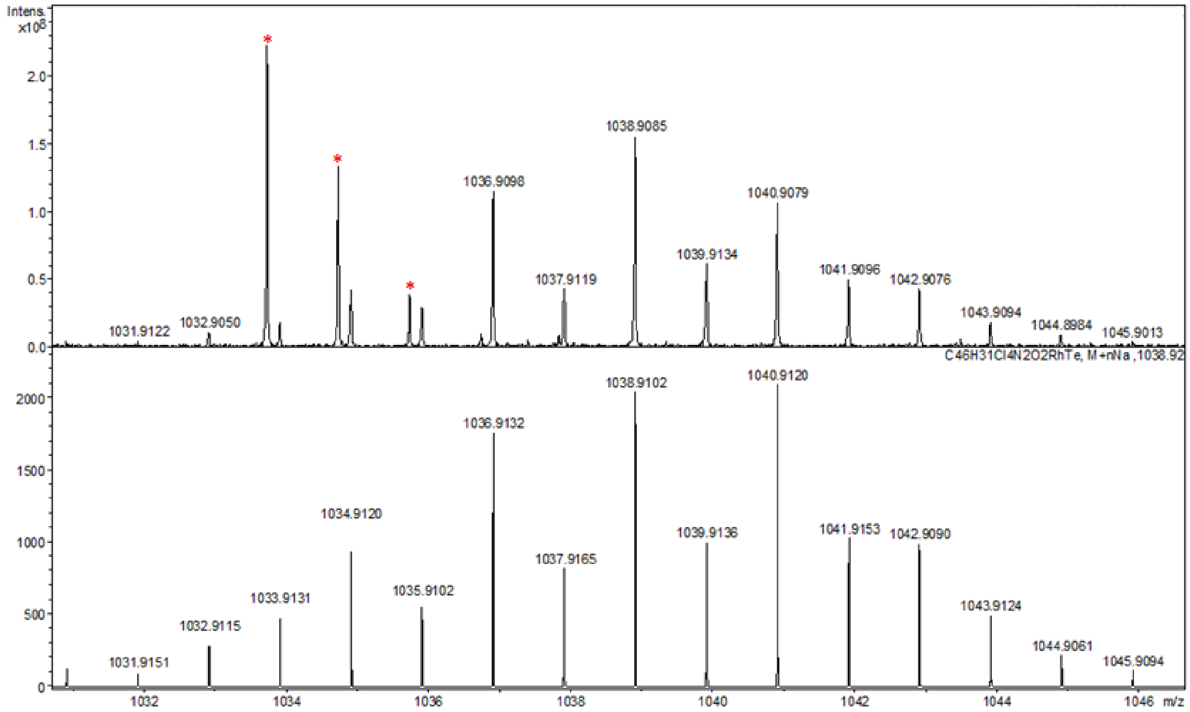

Figure S19. HRMS ESI (+MS) spectra of $\mathbf{2_{B'}}$**-H**: measured (top) and simulated (bottom) calc. for $C_{46}H_{31}Cl_4N_2O_2RhTe$, $[M+Na]^+$.

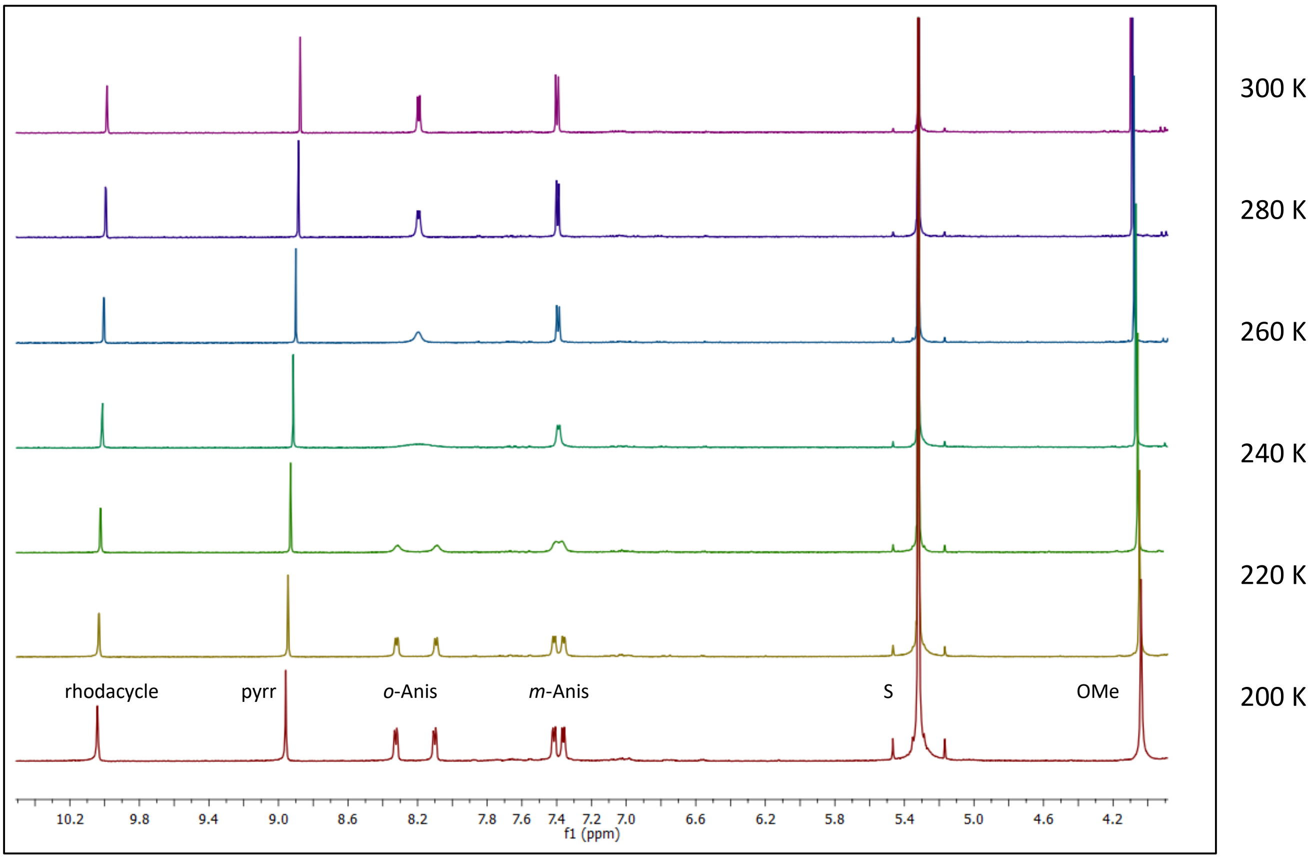

Figure S20. Variable temperature ^1H NMR spectra of $\mathbf{3_A}$; 600 MHz, CD_2Cl_2, 180–300 K.

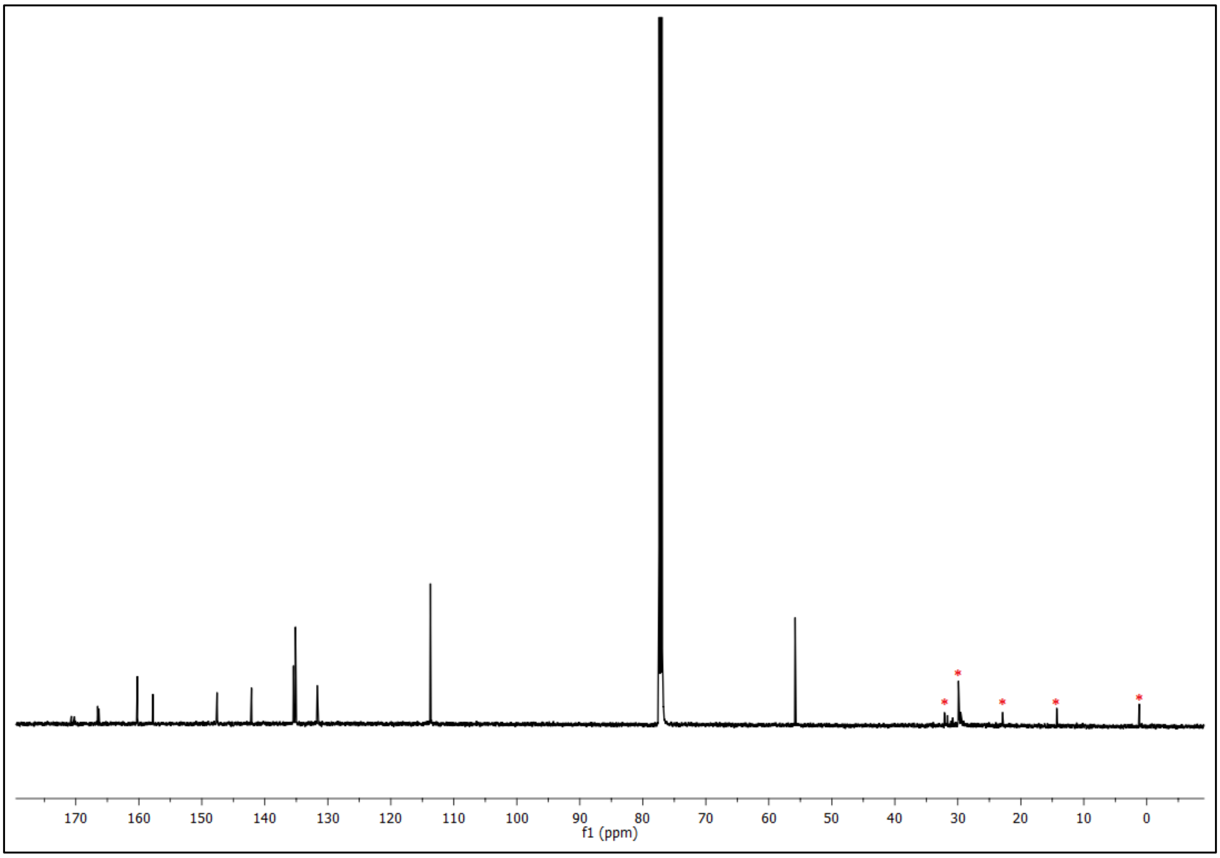

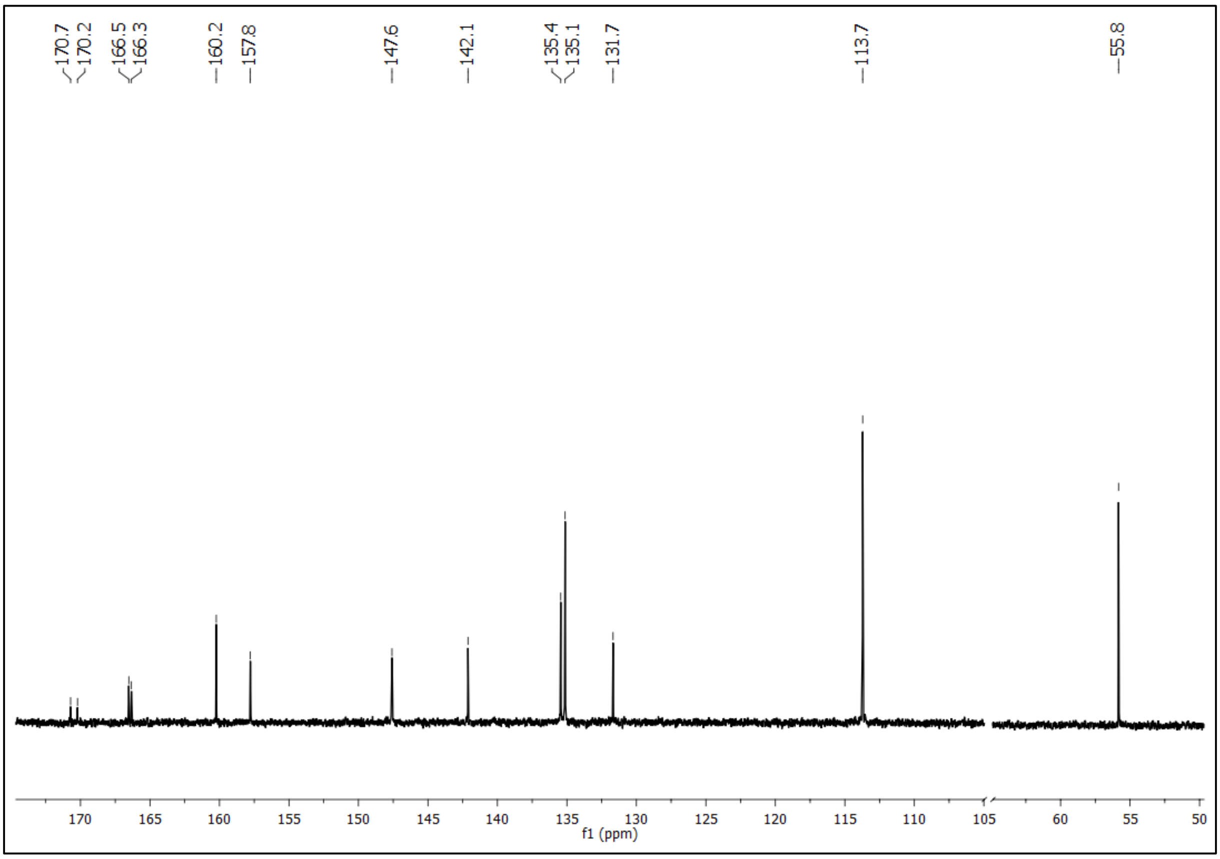

Figure S21. ^{13}C NMR spectrum of **3$_A$;** 150 MHz, $CDCl_3$, 300 K (top: the whole spectral range, bottom: the most informative region; .* = impurities).

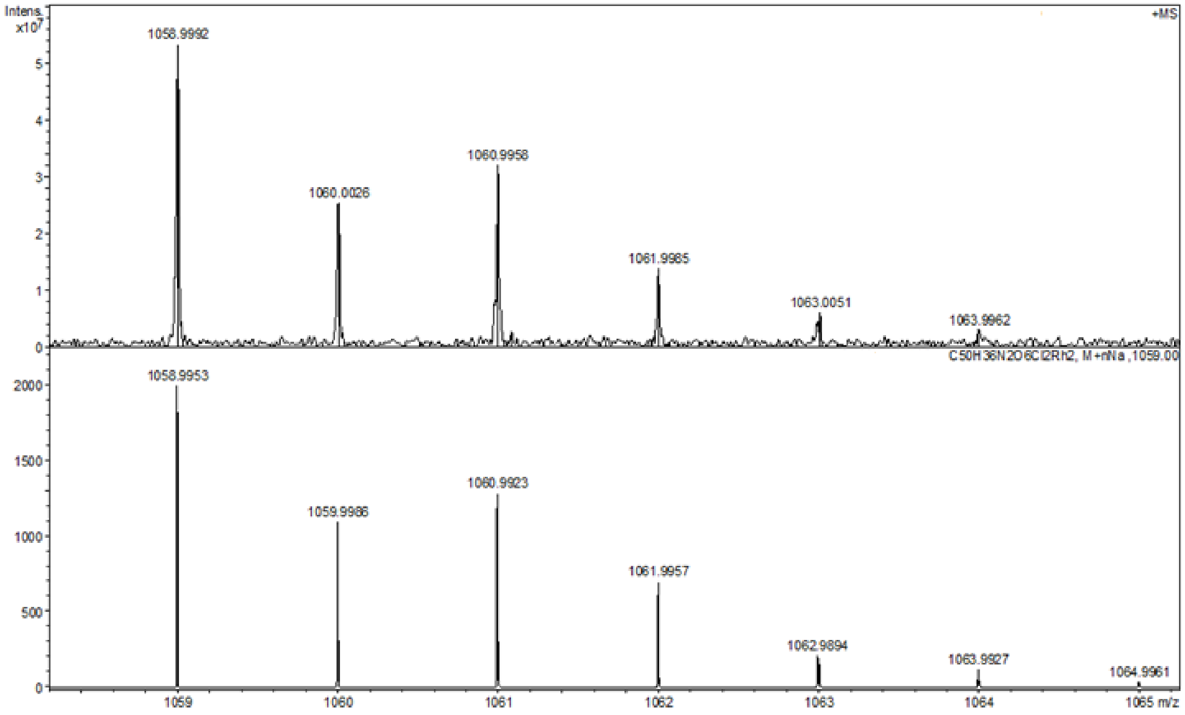

Figure S22. HRMS ESI (+MS) spectra of $\mathbf{3_A}$: measured (top) and simulated (bottom) calc. for $C_{50}H_{36}N_2O_6Cl_2Rh_2$, $[M+Na]^+$.

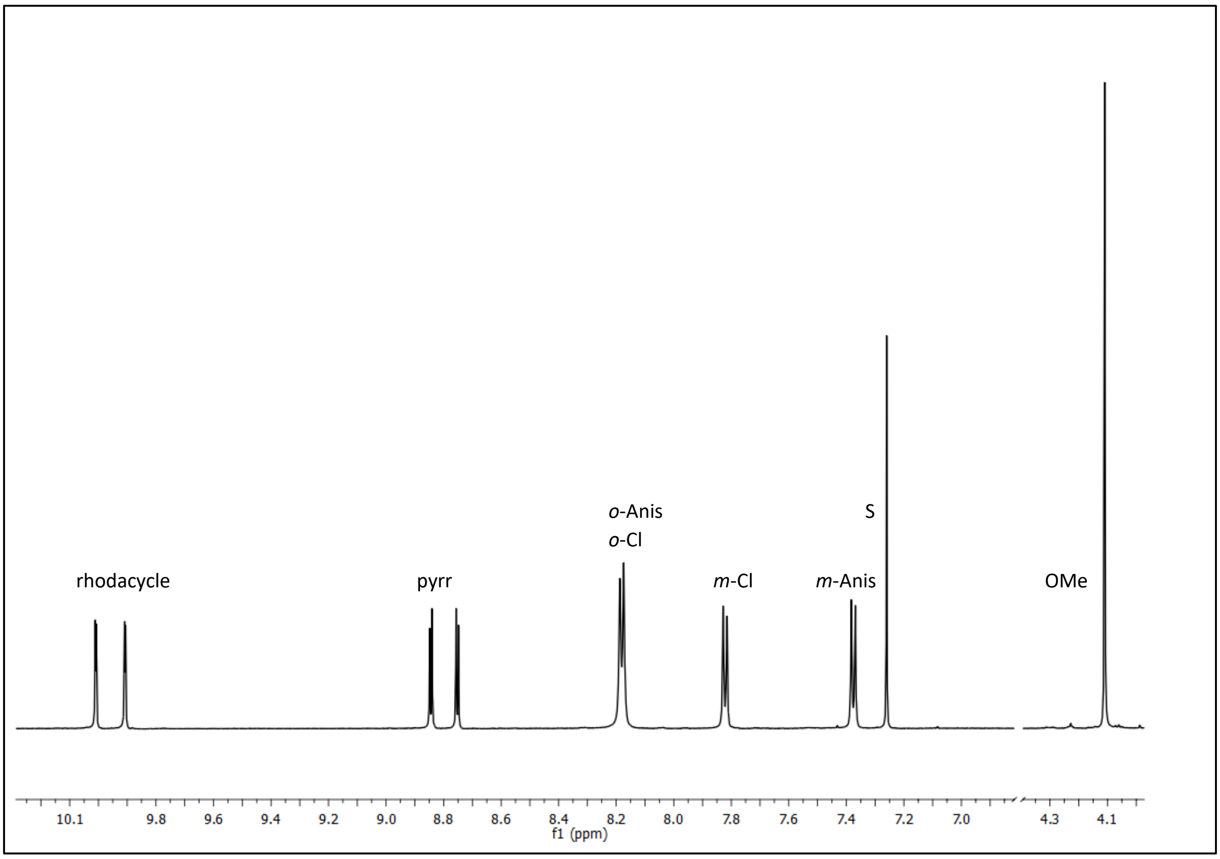

Figure S23. ^1H NMR spectrum of $\mathbf{3_B}$; 600 MHz, $CDCl_3$, 300 K.

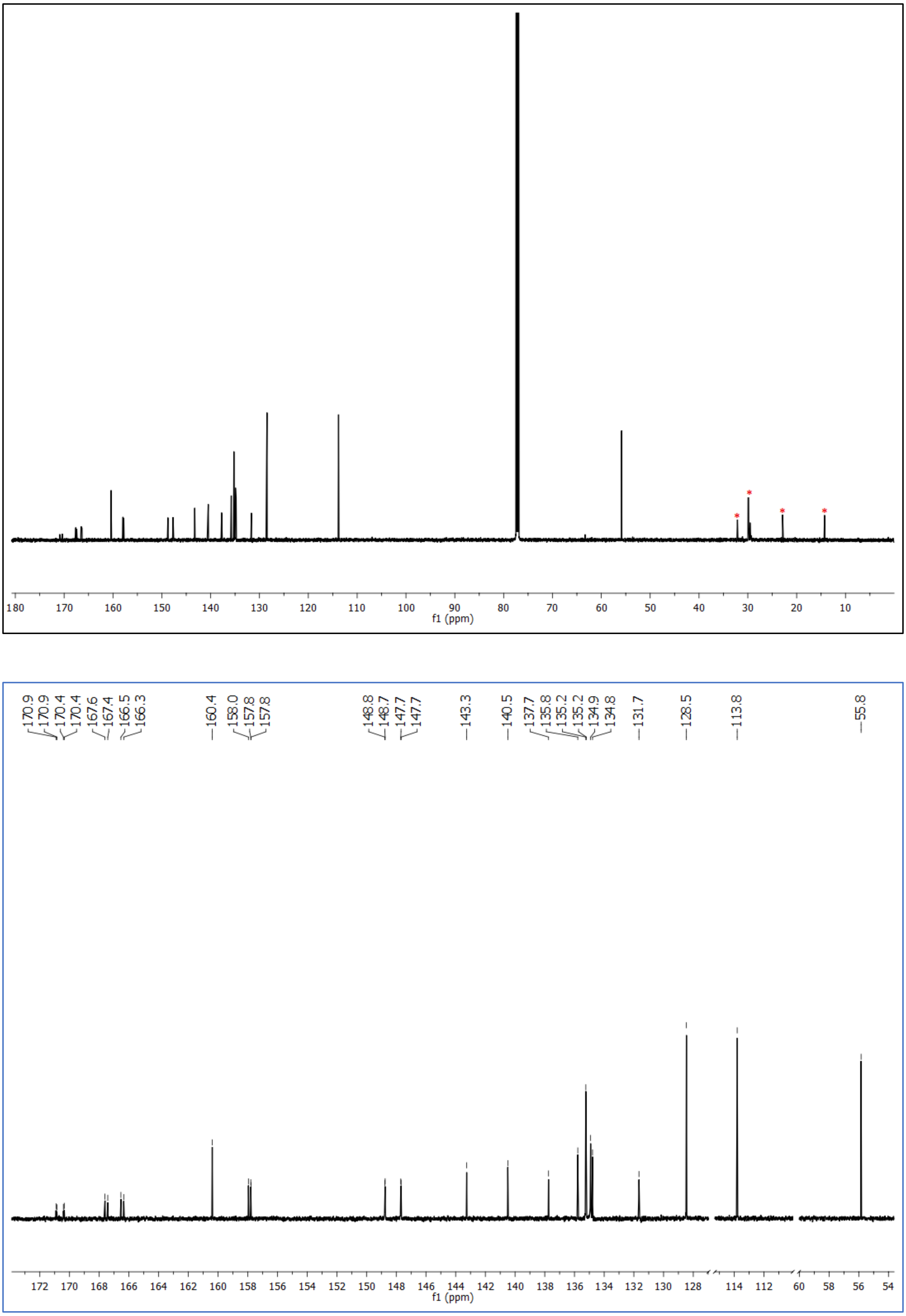

Figure S24. ^{13}C NMR spectrum of $\mathbf{3_B}$; 150 MHz, $CDCl_3$, 300 K (top: the whole spectral range, bottom: the most informative region; .* = impurities).

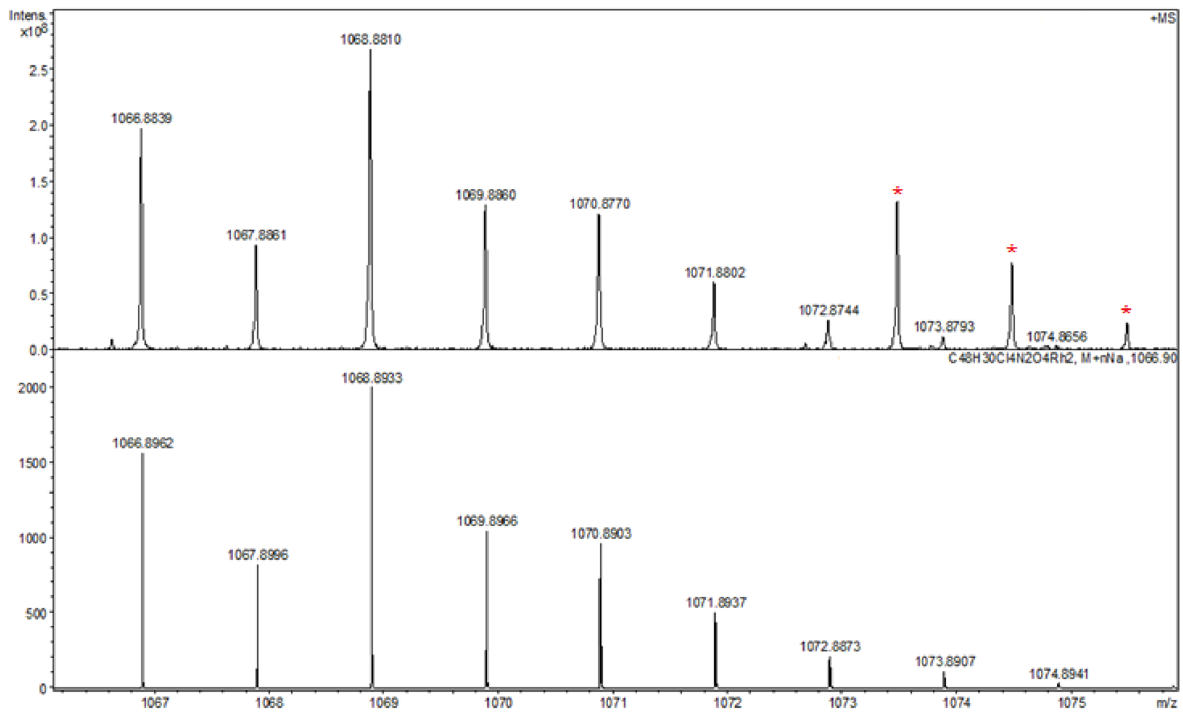

Figure S25. HRMS ESI (+MS) spectra of $\mathbf{3_B}$: measured (top) and simulated (bottom) calc. for $C_{48}H_{30}N_2O_4Cl_4Rh_2$, $[M+Na]^+$.

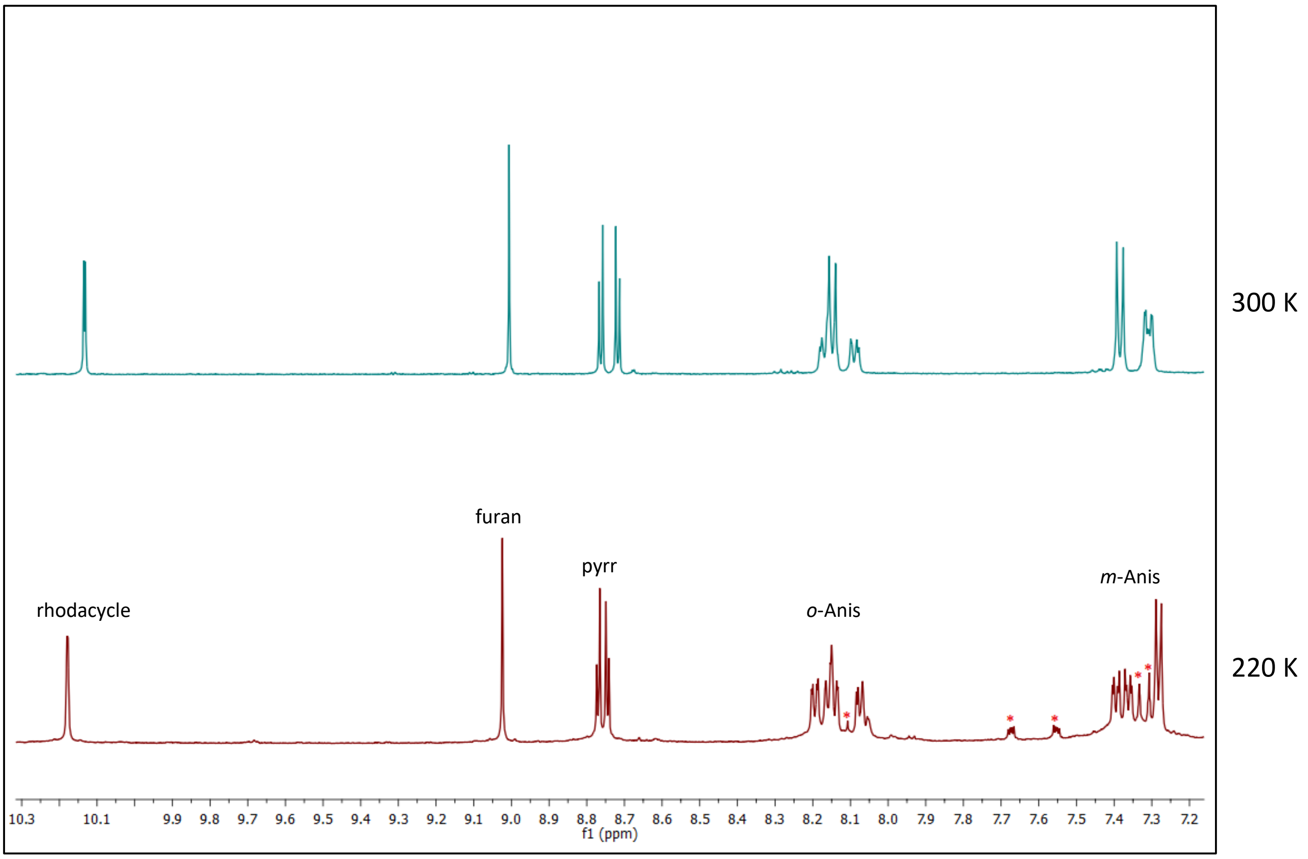

Figure S26. Variable temperature ^1H NMR spectra of $\mathbf{4_A}$; 600 MHz, CD_2Cl_2, 220 K and 300 K.

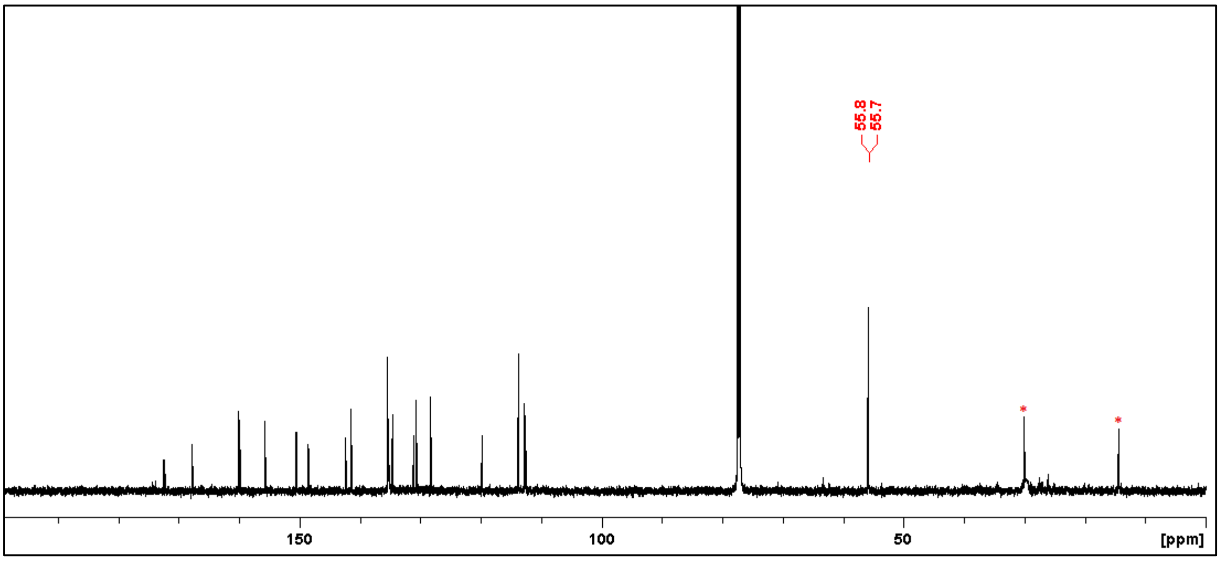

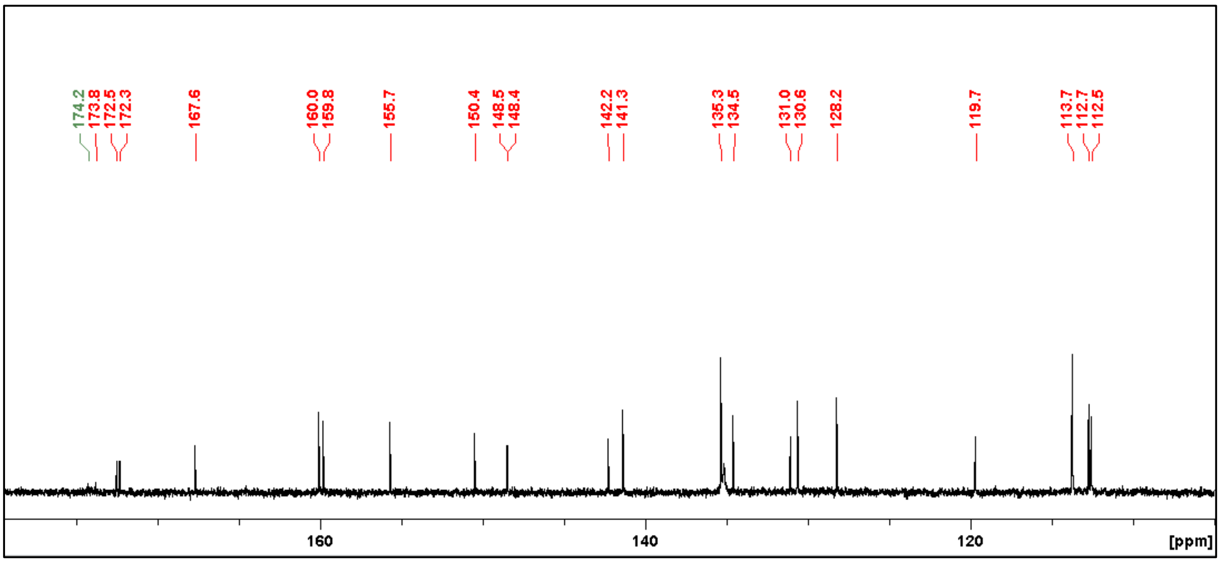

Figure S27. ^{13}C NMR spectrum of $\mathbf{4_A}$; 150 MHz, $CDCl_3$, 300 K (top: the whole spectral range, bottom: the most informative region; .* = impurities).

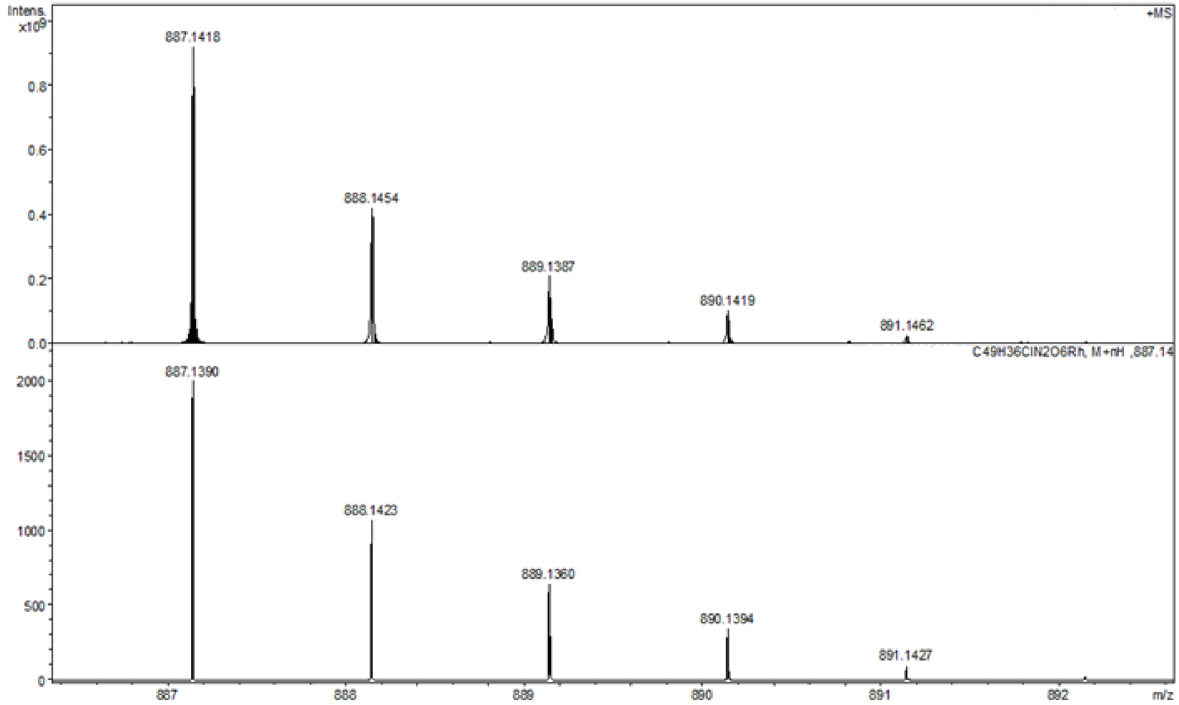

Figure S28. HRMS ESI (+MS) spectra of $\mathbf{4_A}$: measured (top) and simulated (bottom) calc. for $C_{49}H_{36}ClN_2O_6Rh$, $[M+H]^+$.

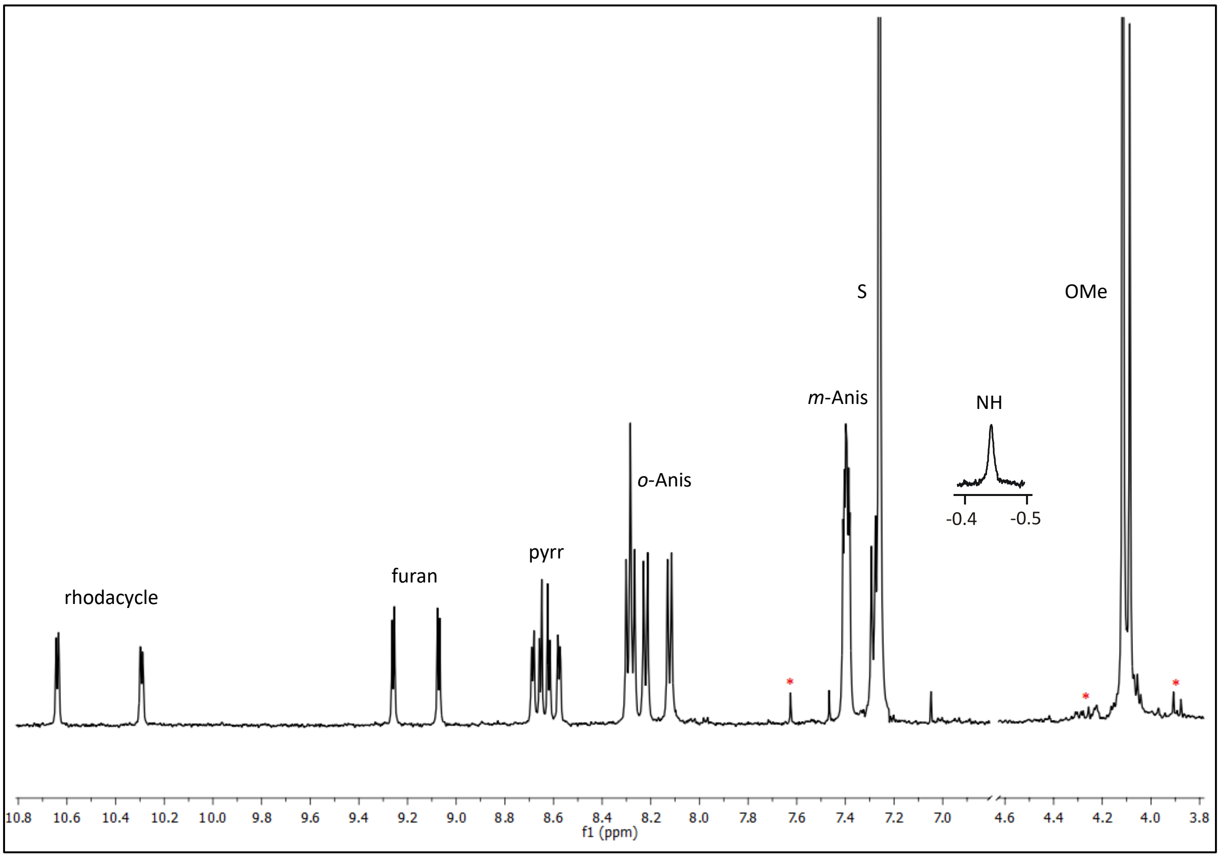

Figure S29. ^{1}H NMR spectrum of $\mathbf{4_A}$**-H**; 500 MHz, $CDCl_3$, 300 K (* = impurities).

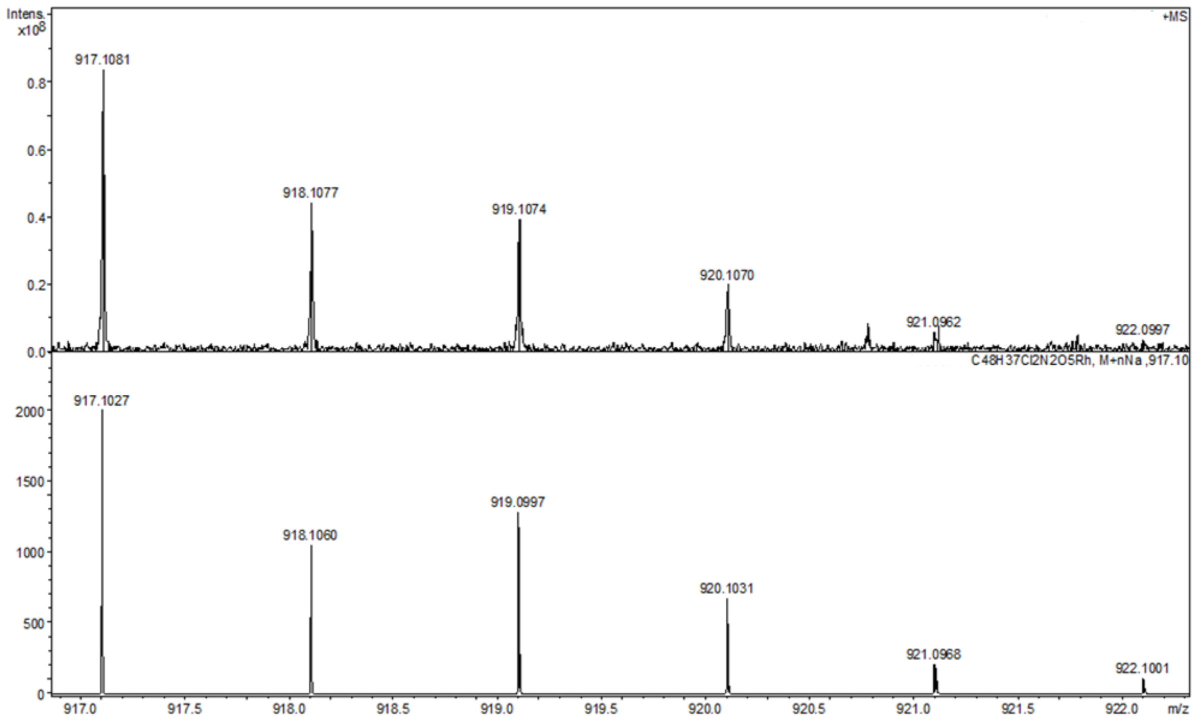

Figure S30. HRMS ESI (+MS) spectra of **4_A-H**: measured (top) and simulated (bottom) calc. for $C_{48}H_{37}Cl_2N_2O_5Rh$, $[M+Na]^+$.

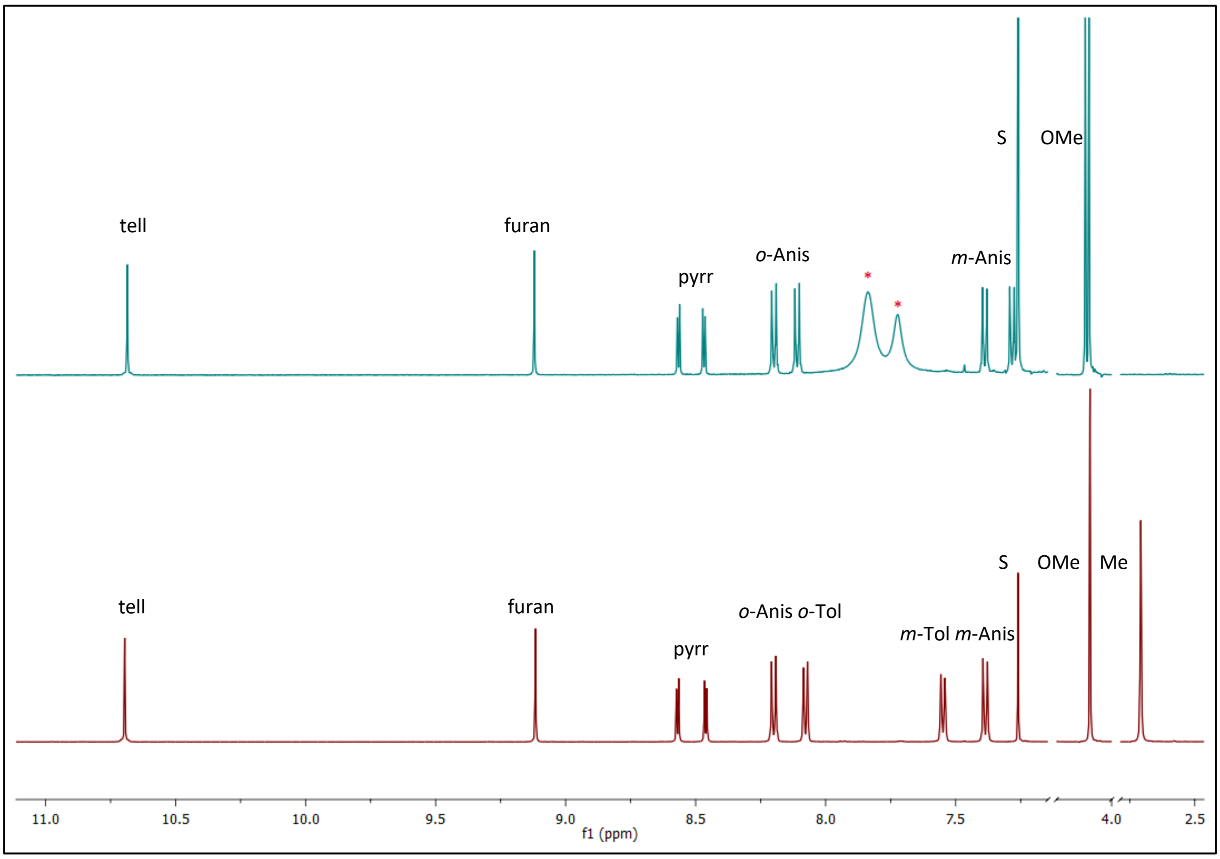

Figure S31. ^{1}H NMR spectra of **5_A** (top) and **5_B** (bottom); 500 MHz, $CDCl_3$, 300 K (* = signals of **1_A**).

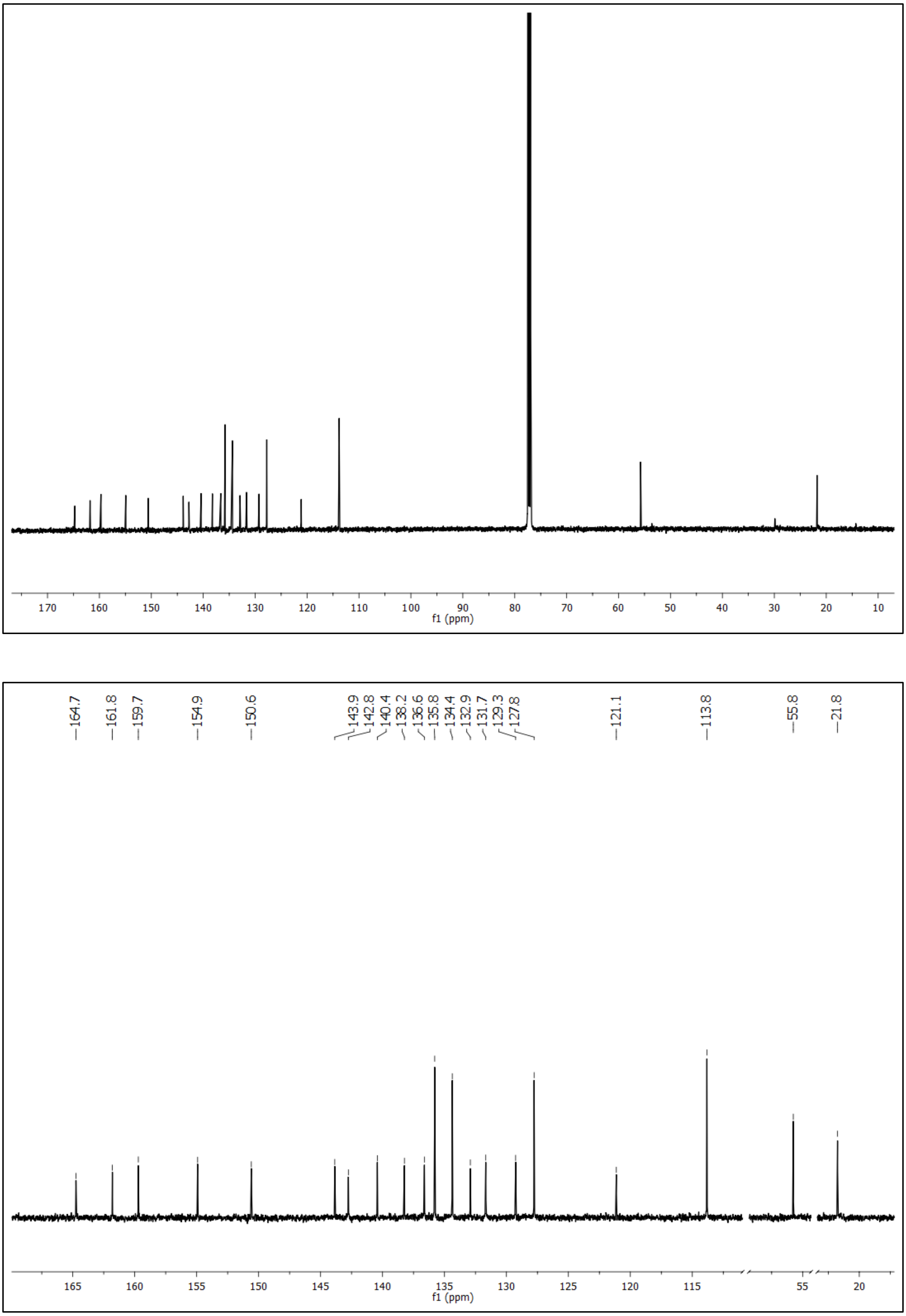

Figure S32. ^{13}C NMR spectrum of **5$_B$**; 125 MHz, $CDCl_3$, 300 K (top: the whole spectral range, bottom: the most informative region).

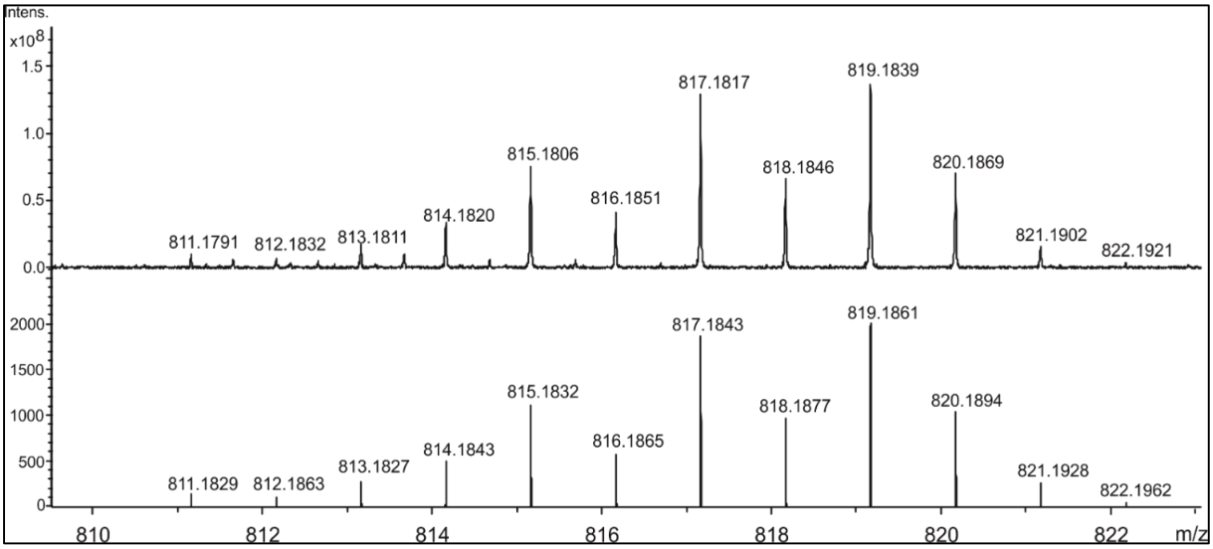

Figure S33. HRMS ESI (+MS) spectra of $\mathbf{5_B}$: measured (top) and simulated (bottom) calc. for $C_{48}H_{36}N_2O_3Te$, $[M+H]^+$.

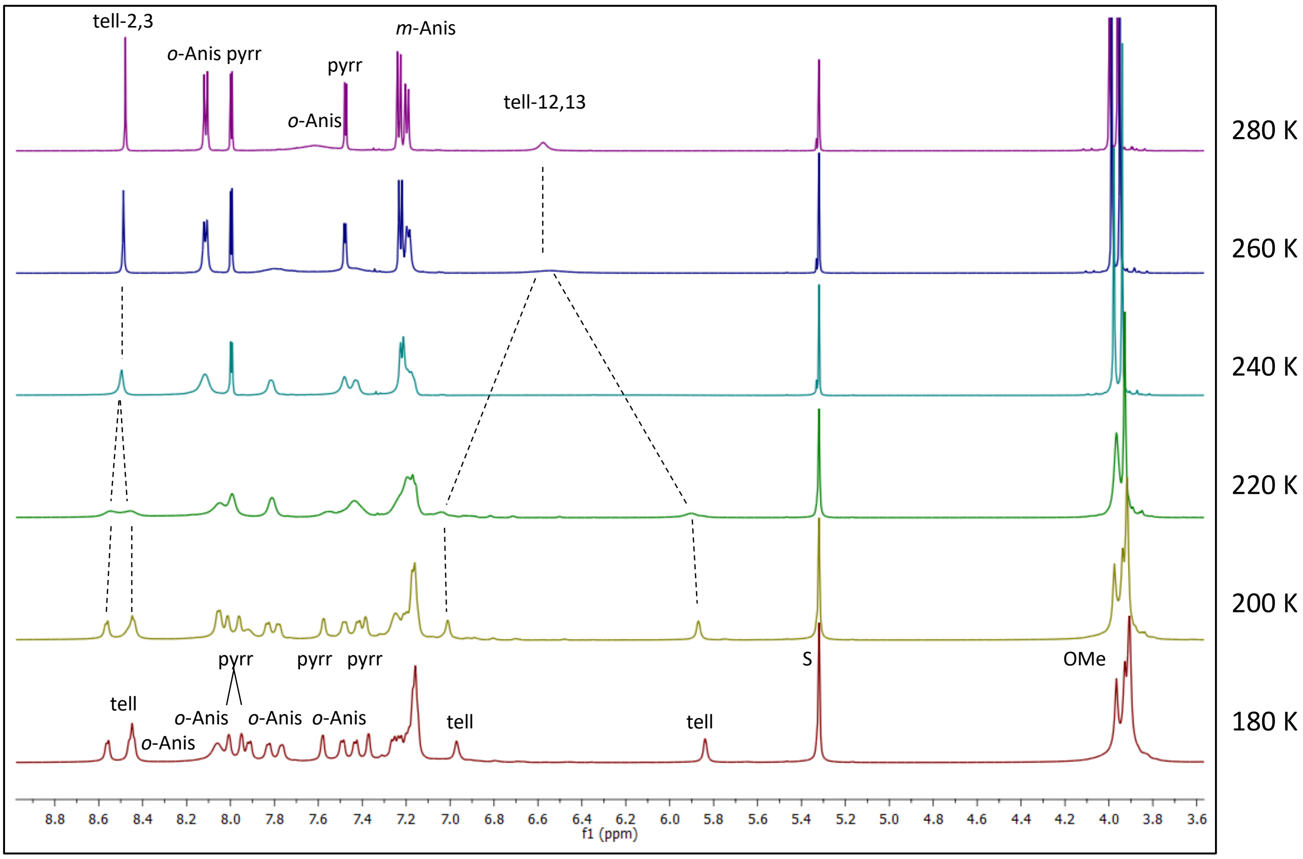

Figure S34. Variable temperature ^{1}H NMR spectra of **8**; 600 MHz, CD_2Cl_2, 180–280 K.

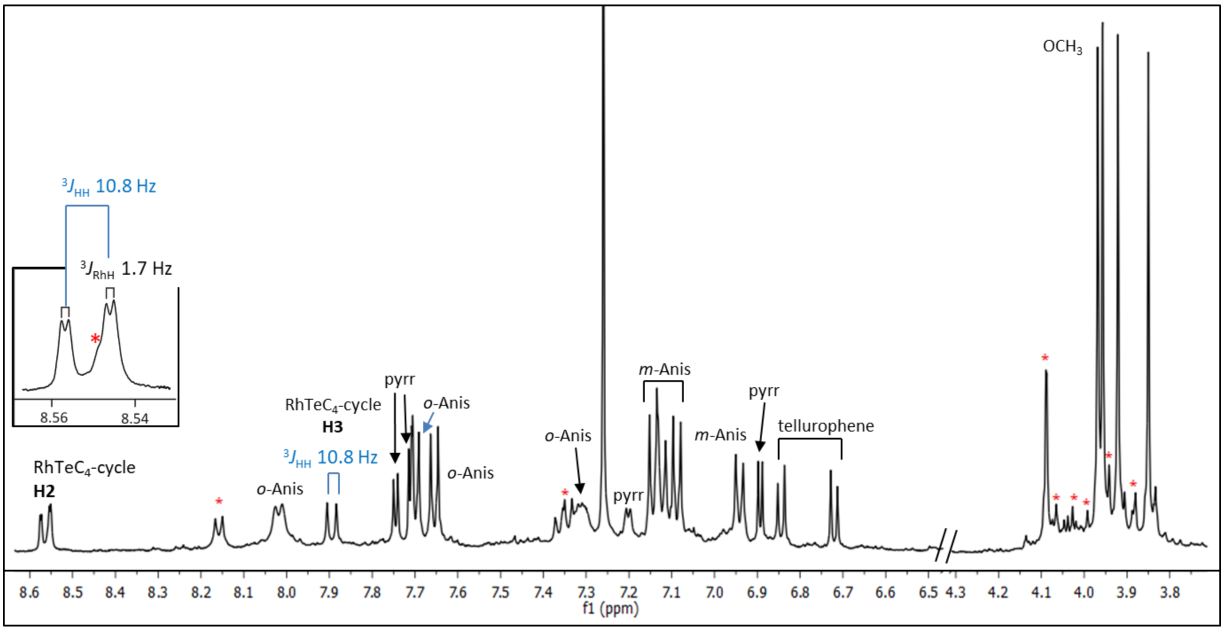

Figure S35. ^{1}H NMR spectra of **9**; 500 MHz, CDCl$_3$, 300 K (* = signals of 2_A and unidentified products).

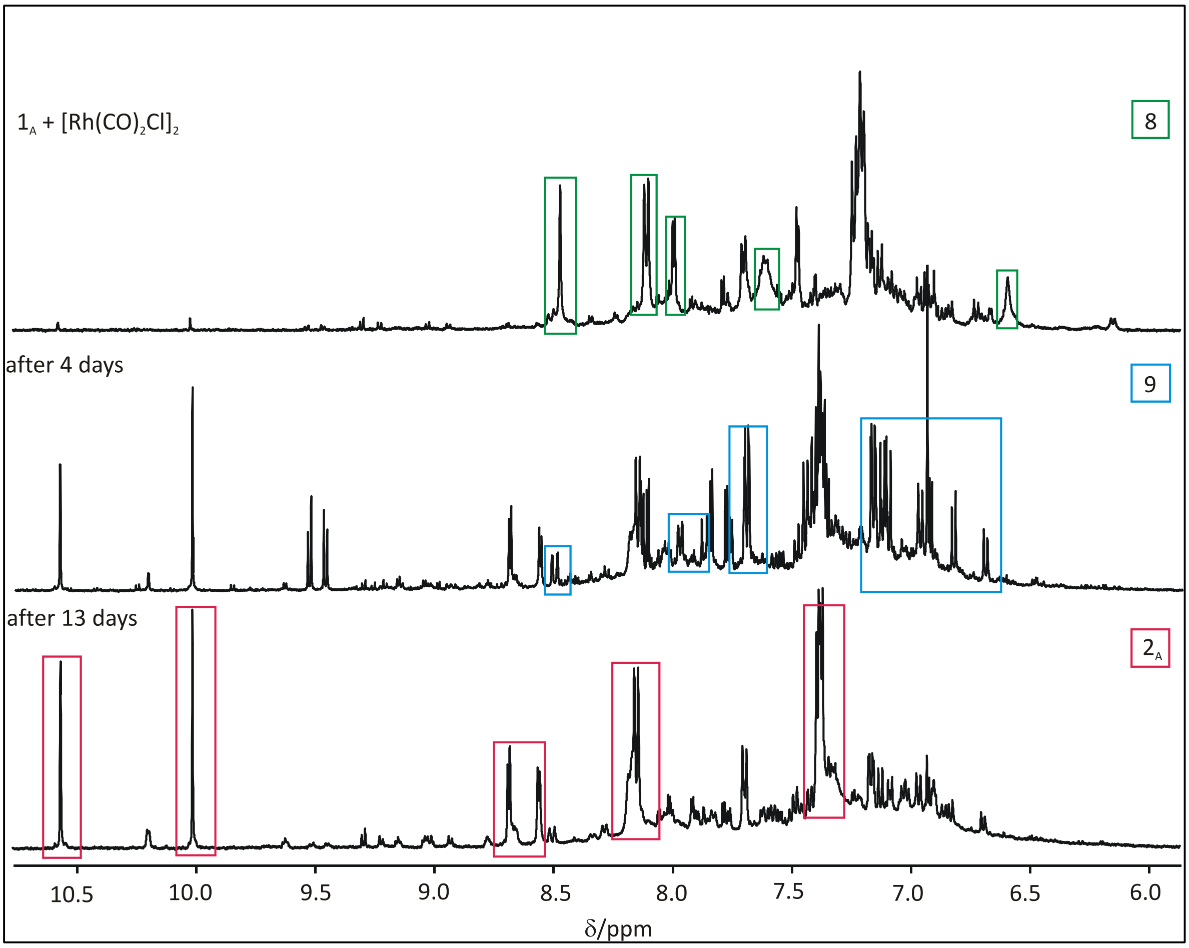

Figure S36. ^{1}H NMR monitored transformation **8** → **9** → **2$_A$**; the most informative spectrum window is chosen; 500 MHz, CD_2Cl_2, 300 K.

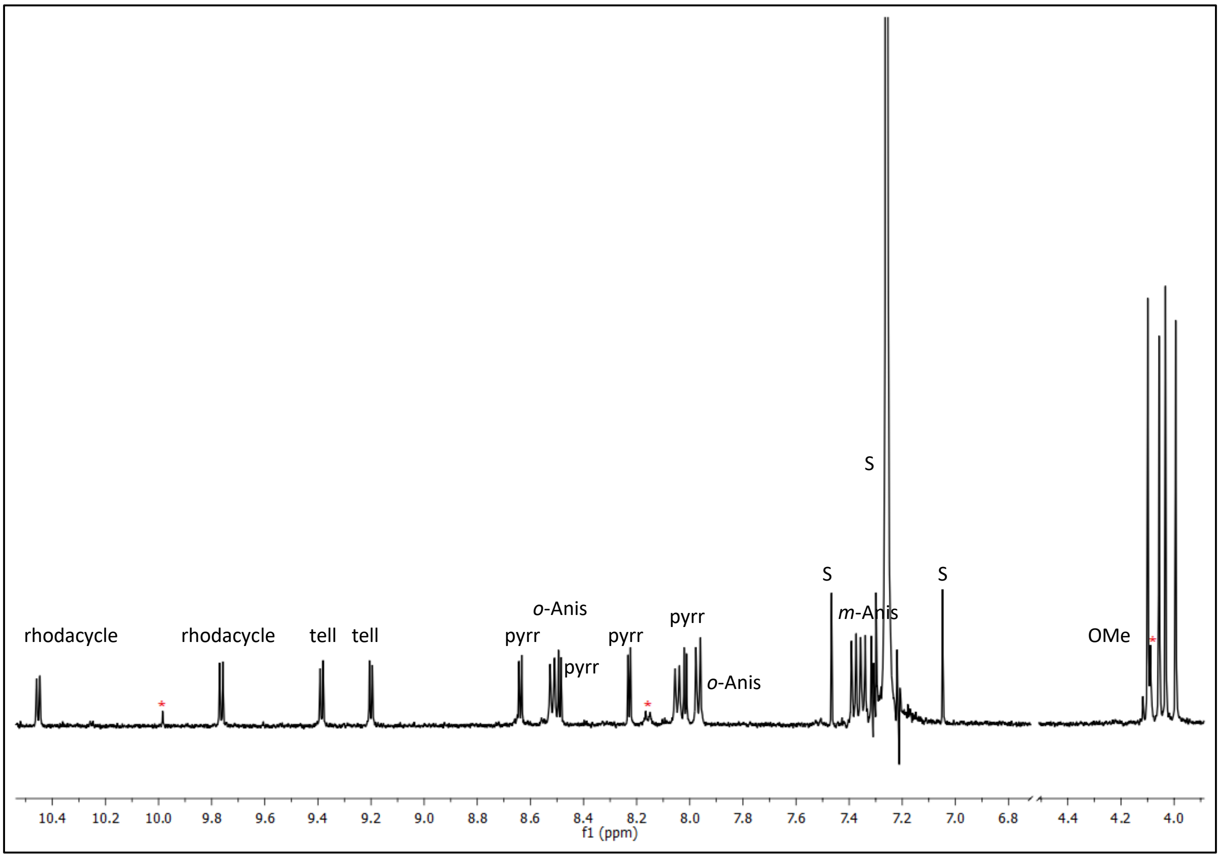

Figure S37. ^{1}H NMR spectrum of **10**; 500 MHz, $CDCl_3$, 300 K (* = signals of $\mathbf{2_A}$).

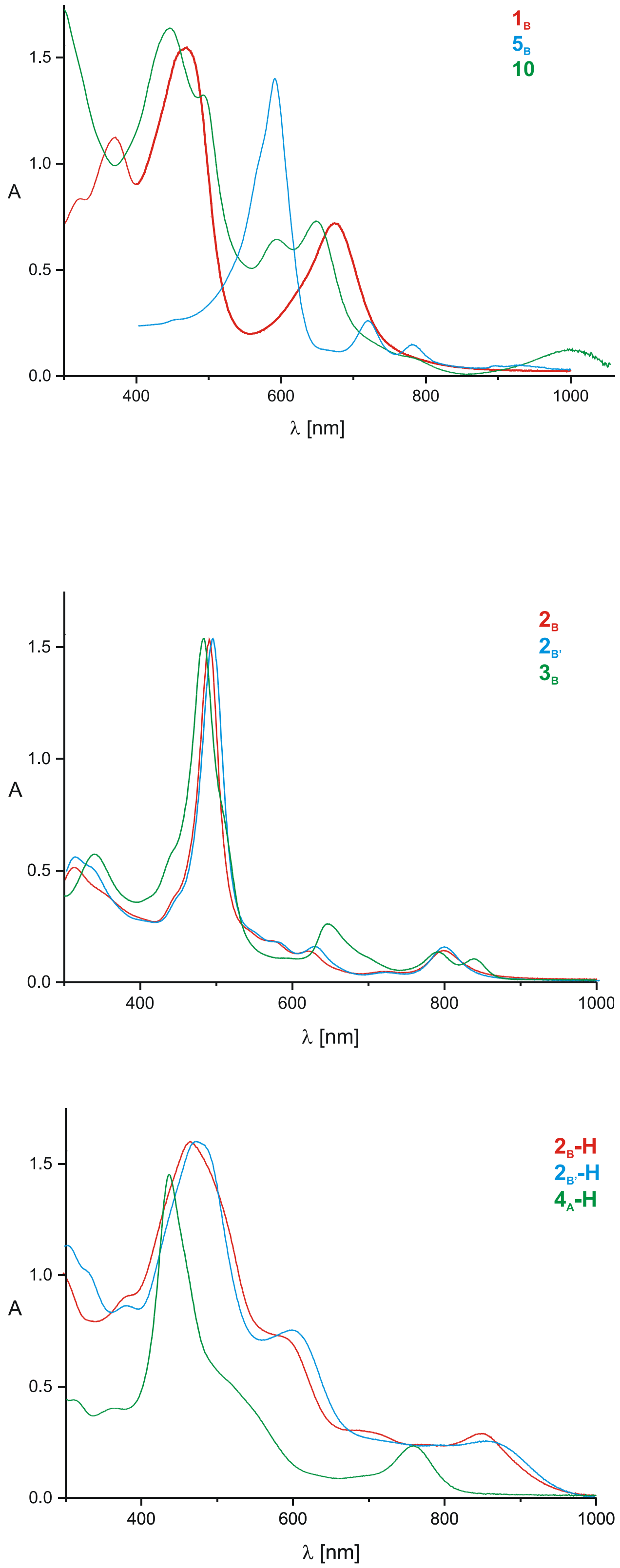

Figure S38. Absorption spectra (CH_2Cl_2) of **1_B, 5_B, 10; 2_B, $2_{B'}$, 3_B; 2_B-H, $2_{B'}$-H, 4_A-H**.

X-ray crystallography

Table S1. Crystal data for **2_A**, **2_A-H**, **3_A**, **4_A**, **5_B**, **1_B**.

	**2_A**	**2_A-H**	**3_A**	**4_A**	**5_B**	**1_B**
crystal dimensions	0.09x0.08x0.01 mm^3	0.32x0.21x0.18	0.12x0.10x0.05	0.09x0.06x0.02	0.15x0.13x0.07	0.38x0.30x0.02
crystal system	monoclinic	monoclinic	orthorhombic	triclinic	triclinic	monoclinic
space group	$P2_1$	$P2_1/c$	Pbca	P-1	P-1	$P2_1/c$
unit cell dimensions:<br>a[Å]<br>b[Å]<br>c[Å]<br>α[]<br>β[]<br>γ[]<br>Z	 15.700(4)<br>8.380(3)<br>18.693(11)<br>90<br>106.70(4)<br>90<br>2	 10.263(4)<br>14.508(2)<br>31.585(8)<br>90<br>93.74(3)<br>90<br>4	 14.7331(7)<br>13.7912(9)<br>20.0213(12)<br>90<br>90<br>90<br>4	 8.4778(2)<br>15.0208(4)<br>15.2897(3)<br>94.496(2)<br>99.237(2)<br>96.560(2)<br>2	 9.637(2)<br>14.986(3)<br>15.090(3)<br>81.24<br>88.09(3)<br>78.92(3)<br>2	 15.303(3)<br>16.643(4)<br>16.564(3)<br>90<br>93.66(2)<br>90<br>4
unit cell volume	2355.6(18) $Å^3$	4693(2)	4068.1(4)	1899.90(8)	2113.7(8)	4210.0(15)
ρ_{calcd}	1.408	1.493	1.694	1.551	1.283	1.528
$2\vartheta_{max}$	2 x 28.826	2x36.954	2x 25.497	2x73.674	2x28.795	2x28.852
radiation, wavelength	Mo Kα, 0.71073 Å	Mo Kα, 0.71073 Å	Mo Kα, 0.71073 Å	Cu Kα, 1.54184 Å	Mo Kα, 0.71073 Å	Mo Kα, 0.71073 Å
scan mode	Ω-scan	Ω-scan	Ω-scan	Ω-scan	Ω-scan	Ω-scan
temperature of measurement	100(2)	100(2)	105(2)	100(2)	80.3(7)	100(2)
no. of measured and independent reflections	8914, 1489	13054, 10598	3776, 2105	7340, 6829	9282, 7762	9519, 4883
no. of reflections included in refinement	8914	13054	3776	7340	9282	9519
absorption corrections, μ, min/max transmission	none,<br>μ = 1.071	multi-scan,<br>μ= 1.195; 0.797-1.000	analytical absorption correction performed, μ= 1.000; 0.916-0.963	analytical absorption correction performed, μ= 4.752; 0.708-0.904	empirical, μ=0.745; 0.896-0.950	analytical absorption correction performed, μ= 1.551; 0.587-0.969
method of structure solution and program	SHELXS-2018/3 (Sheldrick, 2018)	SHELXS-2018/3 (Sheldrick, 2018)	SHELXS-2018/3 (Sheldrick, 2018)	SHELXS-2018/3 (Sheldrick, 2018)	SHELXS-2014/7 (Sheldrick, 2014)	SHELXS-2018/3 (Sheldrick, 2018)
method of refinement and program	SHELXL-2018/3 (Sheldrick, 2018)	SHELXL-2018/3 (Sheldrick, 2018)	SHELXL-2018/3 (Sheldrick, 2018)	SHELXL-2018/3 (Sheldrick, 2018)	SHELXL-2014/7 (Sheldrick, 2014)	SHELXL-2018/3 (Sheldrick, 2018)
no. of parameters	486	998	280	572	508	585
treatment of H atoms	constrained	constrained	constrained	constrained	constrained	constrained
R, wR for reflections with I > 2 σ(I)	0,1528, 0,3346	0.1273, 0.2925	0,0778; 0,1286	0,0485; 0,1082	0.0433, 0.0952	0.1477, 0.3132
residual electron density	3.193, -1.192	2.664, -4.257	1.009; -0.963	0.790; -0.680	2.321; -0.505	3.124, -2.039
**CCDC number**	2128521	2128522	2128523	2128524	2128525	2128526

The X-ray diffraction data were collected on the four-circle diffractometer (with Ruby detector: **1$_B$**, **2$_A$**, **3$_A$**, **5$_B$**, with Sapphire2 detector: **2$_A$-H**, with HyPix-Arc 150 detector: **4$_A$**) using MoKα radiation (λ = 0.71073 Å) for **2$_A$**, **2$_A$-H**, **3$_A$**, **5$_B$**, **1$_B$** or CuKα radiation (λ = 1.54175 Å) for **4$_A$**. The data were collected at 80.3(7) K (**5$_B$**), 100(2) K (**2$_A$**, **2$_A$-H**, **4$_A$**, **1$_B$**) or 105(2) K (**3$_A$**) by using an Oxford Cryosystem device. Data reduction and analysis were carried out with the CrysAlis programs.[4] The space groups were determined, based on systematic absences and intensity statistics. The structures were solved by direct methods by using the SHELXS program and refined by using all F2 data as implemented within the SHELXL program.[5]

For **2$_A$**, **2$_A$-H**, **5$_B$**, **1$_B$** SQUEEZE procedure was applied for disordered solvent molecules. Non-H atoms were refined with anisotropic displacements, however because of a low quality of diffraction data (**2$_A$**) or disorder (**1$_B$**, **2$_A$-H**, **3$_A$**, **4$_A$**, **5$_B$**) SIMU and/or ISOR restrains were applied for non-H atoms. In **4$_A$**, EXYZ and EADP commands were applied for disordered N22 and O23a and O23 and N22a (N22, O23 – major components of the disorder with occupancy factor equal to 0.87). In **2$_A$-H** the compound reveals at least two positioned disorder. Occupancy factors were refined and for the major component of the disorder is equal to 0.88. Except the Cl1 atom, all atoms of both components of disorder are separated. SADI command was applied for pyrrole ring and methoxy groups (O49-C52/O49a-C52a and O42-C45/O42a-C45a), and FLAT command for pyrrole rings of minor component of the disorder. Moreover, for the Rh21 atom and for its minor counterpart Rh41 (and also for N24 and N24a) EADP command was applied.

EADP command was applied for Rh21 and Rh41 (with occupancy factors: 0.88 and 0.12, respectively) and for N24 and N24a

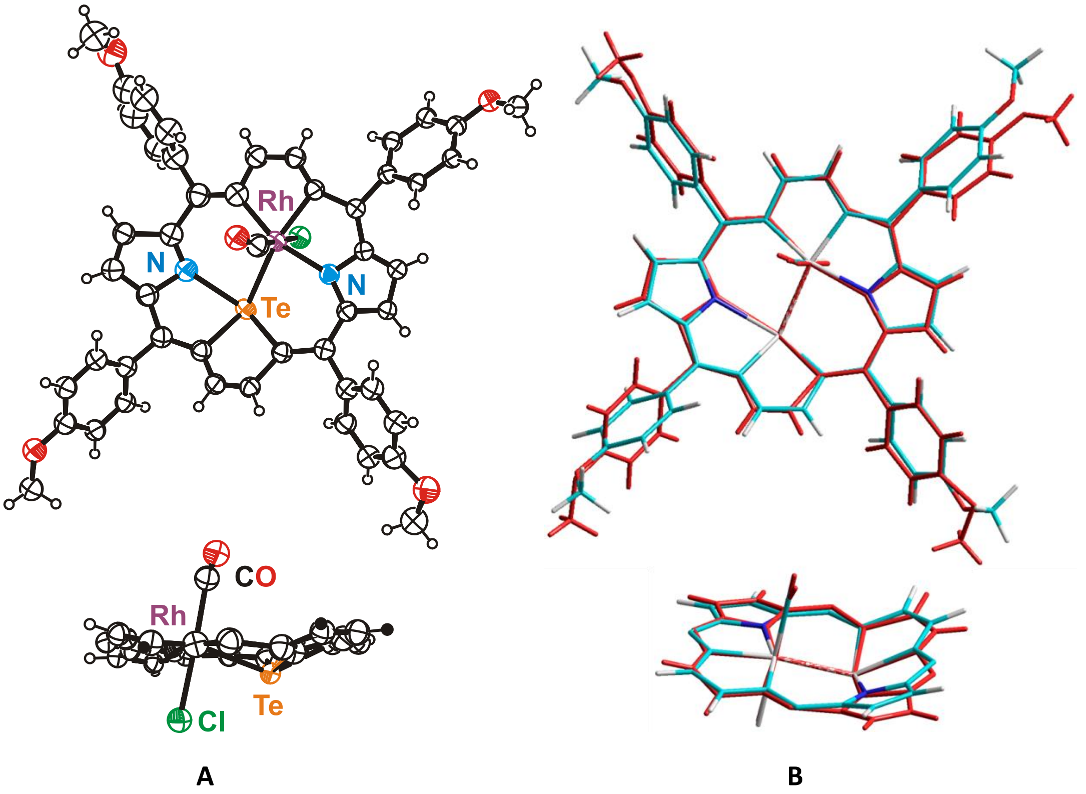

Figure S39. A: X-ray molecular structure of **2$_A$**. Displacement ellipsoids represent 50% probability. In the side view (bottom), the aryl rings are omitted for clarity. **B**: Comparison of the X-ray (red) and DFT (blue) structures.

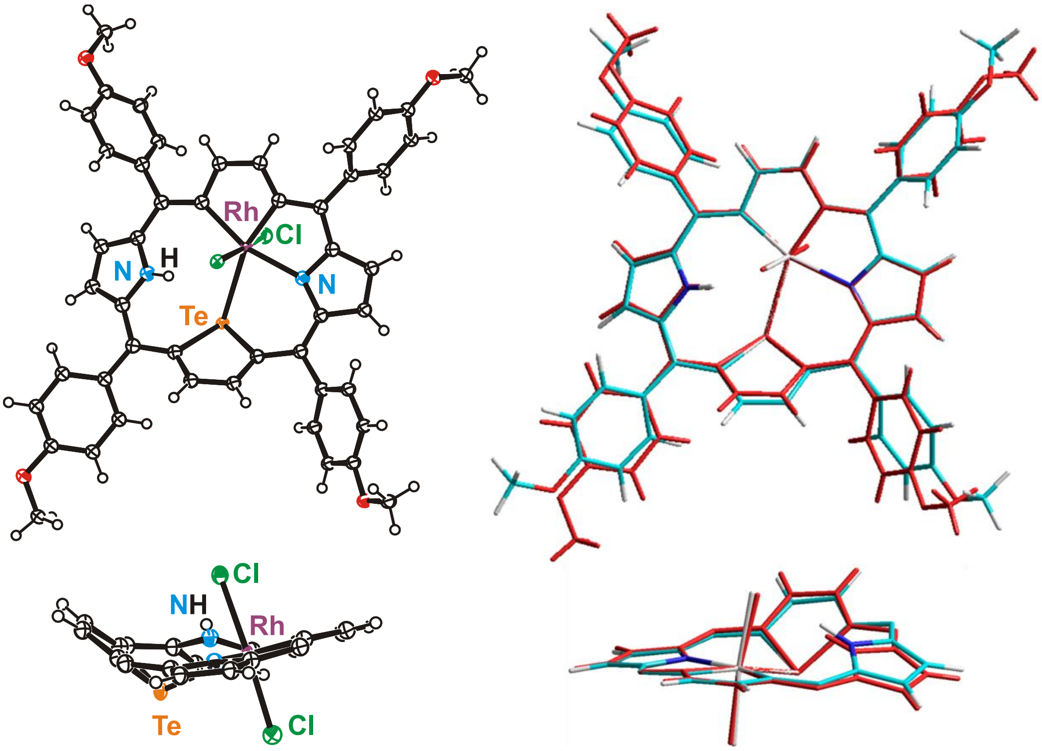

A B

Figure S40. A: X-ray molecular structure of **2_A-H**. Displacement ellipsoids represent 50% probability. In the side view (bottom), the aryl rings are omitted for clarity. **B**: Comparison of the X-ray (red) and DFT (blue) structures.

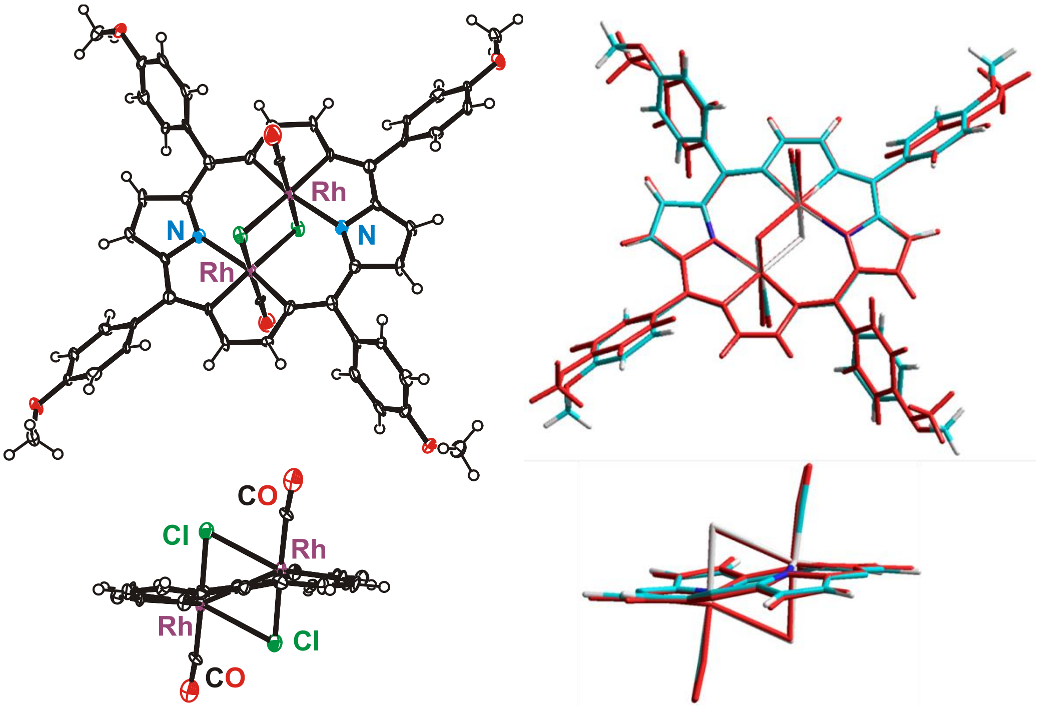

A B

Figure S41. **A**: X-ray molecular structure of **3_A**. Displacement ellipsoids represent 50% probability. In the side view (bottom), the aryl rings are omitted for clarity. **B**: Comparison of the X-ray (red) and DFT (blue) structures.

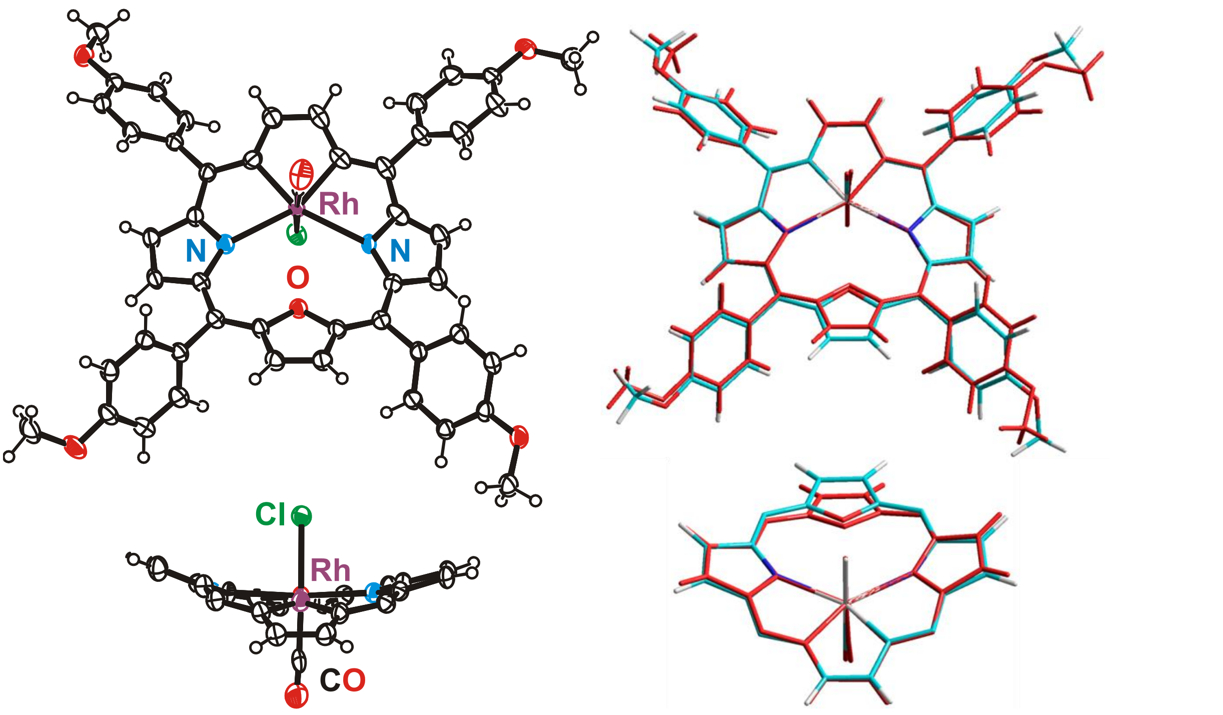

A **B**

Figure S42. **A:** X-ray molecular structure of $\mathbf{4_A}$. Displacement ellipsoids represent 50% probability. In the side view (bottom), the aryl rings are omitted for clarity. **B**: Comparison of the X-ray (red) and DFT (blue-white) structures.

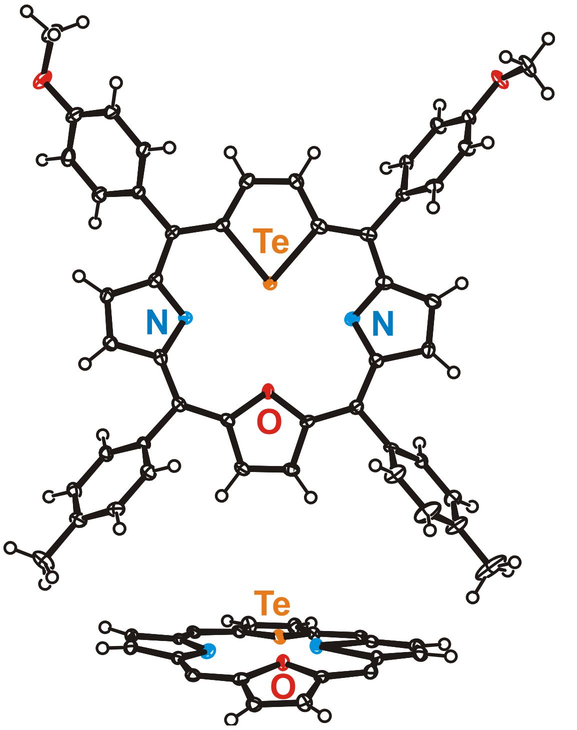

Figure S43. A: X-ray molecular structure of **5**. Displacement ellipsoids represent 50% probability. In the side view (bottom), the aryl rings are omitted for clarity.

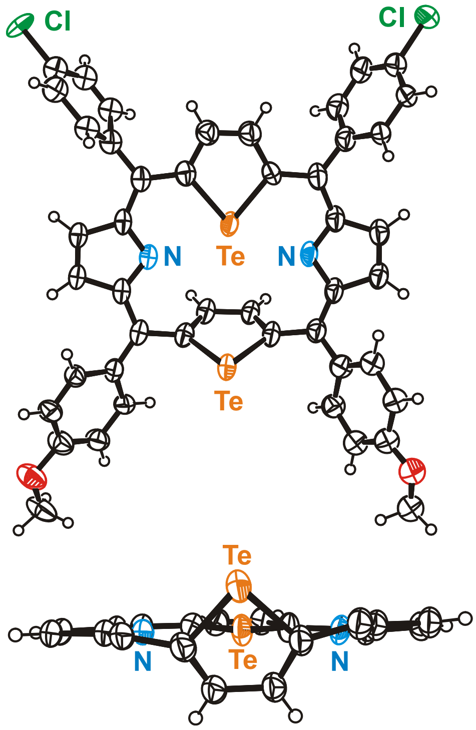

Figure S44. X-ray molecular structure of $\mathbf{1_B}$. Displacement ellipsoids represent 50% probability. In the side view (bottom), the aryl rings are omitted for clarity.

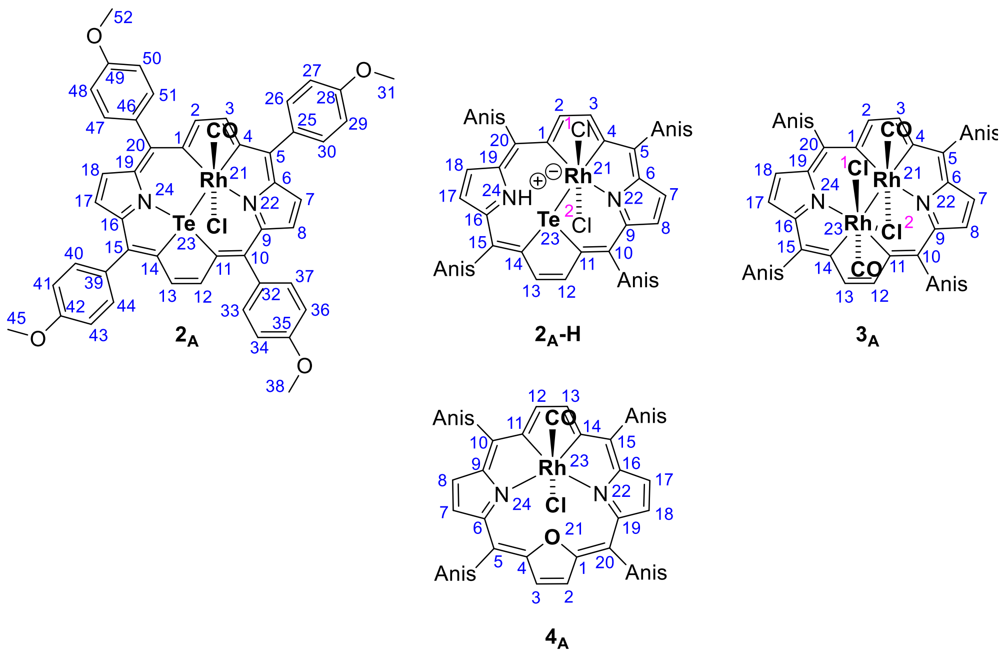

Scheme S1. Numbering scheme for X-ray data of **2_A, 2_A-H, 3_A** and **4_A.**

Table S2. Selected geometric parameters for X-ray structures and DFT-optimized structures of **2_A, 2_A-H, 3_A** and **4_A**.

	**2_A**		**2_A-H**		**3_A**		**4_A**		**8**
	**X-ray**	**DFT**	**X-ray**	**DFT**	**X-ray**	**DFT**	**X-ray**	**DFT**	**DFT**
C_{meso}·C_{meso}·C_{meso}(acute) angle (°)	80<br>82	81<br>81	82<br>84	83<br>84	79	79	87<br>88	87<br>87	88<br>87
(Rh·C1·C2·N·Te) plane – – tellurophene plane (°)[a]<br>Rh·N22·Te·Cl·C(O) plane – – tellurophene plane (°)[a]	34<br>–	38<br>–	59<br>–	51<br>–	–<br>–	–<br>–	–<br>–	–<br>–	–<br>87 (Te21)<br>17 (Te23)
$C_{4\text{-}meso}$–rhodacycle plane (°)[a]<br>$C_{4\text{-}meso}$–Rh·N22·Te·Cl·C(O) plane[a]	7<br>–	7<br>–	12<br>–	11<br>–	12<br>–	10<br>–	10<br>–	6<br>–	–<br>74
$C_{4\text{-}meso}$–tellurophene plane (°)[a]<br>$C_{4\text{-}meso}$-–furan plane (°)[a]	23<br>–	25<br>–	42<br>–	35<br>–	–<br>–	–<br>–	–<br>21	–<br>14	14 (Te21)<br>70 (Te23)<br>–
$C_{4\text{-}meso}$–pyrrole (N24) plane (°)[a]	13	19	24	26	13	11	23	12	3
$C_{4\text{-}meso}$–pyrrole (N22) plane (°)[a]	7	9	7	7	13	11	14	12	17

a – interplanar angles

Table S3. Selected bond lenghts and distances (Å) for X-ray structures and DFT-optimized structures **2_A, 2_A-H, 3_A** and **4_A**.

	**2_A**		**2_A-H**		**3_A**		**4_A**	
**distance**	**X-ray**	**DFT**	**X-ray**	**DFT**	**X-ray**	**DFT**	**X-ray**	**DFT**
Rh⋯X	2.633(5)<br>(X = Te)	2.657	2.7763(14)<br>(X = Te)	2.789	2.867(1)<br>(X = Rh)	2.908	3.22<br>(X = O)	3.293
Rh–N	2.110(4)	2.132	2.169(11)	2.166	2.131(7)	2.128	2.376(3)<br>(N22)<br>2.357(3)<br>(N24)	2.386<br><br>2.388
Rh–C1/Rh–C11	2.085(6)	2.068	1.947(13)	2.042	2.046(9)	2.043	2.019(4)	1.990
Rh–C4/Rh–C14	2.146(5)	1.999	2.076(13)	1.945	1.978(8)	1.971	2.012(4)	1.990
Te–N	2.375(5)	2.436	-	-	-	-	-	-
Rh–L_{axial}	2.056(7)<br>(CO)<br>2.358(19)<br>(Cl)	1.852<br>2.403	2.359(3) (Cl1)<br>2.345(3) (Cl2)	2.378<br>2.380	1.830(9) (CO)<br>2.712(2) (Cl1)<br>2.349(2) (Cl2)	1.852<br><br>2.749<br><br>2.411	1.916(5)<br>(CO)<br>2.339(1)<br>(Cl)	1.860<br><br>2.358
Rh⋯N (the distant nitrogen)	3.24	3.365	3.34	3.317	3.43	3.474	-	-
Te⋯N (the distant nitrogen)	3.25	3.344	3.28	3.296	-	-	-	-
bond lengths in rhodacycle:<br>C1–C2/C11–C12 ($C_{\alpha}C_{\beta}$);<br>C2–C3/C12–C13 ($C_{\beta}C_{\beta}$);<br>C3–C4/C13–C14 ($C_{\alpha}C_{\beta}$)	too poor crystal quality	1.425<br>1.389<br>1.400	1.385(18)<br>1.382(18)<br>1.431(18)	1.429<br>1.381<br>1.406	1.389(11)<br>1.395(11)<br>1.372(12)	1.419<br>1.395<br>1.398	1.391(6)<br>1.372(6)<br>1.404(6)	1.411<br>1.382<br>1.411
bond lengths in heterocycle:<br><br>C11–C12 ($C_{\alpha}C_{\beta}$);<br>C12–C13 ($C_{\beta}C_{\beta}$);<br>C13–C14 ($C_{\alpha}C_{\beta}$)	too poor crystal quality	(tellurophene)<br><br>1.408<br>1.397<br>1.403	(tellurophene)<br><br>1.402(18)<br>1.410(18)<br>1.383(18)	1.401<br>1.394<br>1.406	-	-	furan<br><br>1.399(5)<br>1.372(5)<br>1.399(5)	1.408<br>1.378<br>1.408

Density Functional Theory Calculations

Density functional theory (DFT) calculations were performed using the Gaussian 16 program.[6] DFT geometry optimizations were carried out in the unconstrained C_1 symmetry in vacuo, using the X-ray structures or molecular mechanics models as starting geometries. The existence of a local energy minimum was verified by a normal mode frequency calculation. DFT calculations were performed using the hybrid functional b3pw91 and a combined basis set consisting of SDD pseudopotential for Rh and Te atoms and 6-31G(d,p) for remaining atoms. The combination B3LYP/LANL2DZ-6-31G(d,p) used in our previous work for analogous platinum(II) and platinum(IV) complexes did not work properly in case of rhodaporphyrins, i.e., did not model the geometry of Rh_2Cl_2 central unit of 21,23-dirhodaporphyrin **3_A** in accord with the crystallographically obtained structure.

All relative energies include the zero-point correction. Transition states were refined using TS optimization (the Berny algorithm). One imaginary frequency was found as a confirmation of a saddle-point achievement. The imaginary frequency of **$[2_A]^‡$** and **$[3_A]^‡$** is associated with a vibration representing a swinging motion of the macrocyclic skeleton.

Calculations for 2

In the main text, the swich between two enantiomers of **2**, i.e., **2a** and **2b,** is described. To make the picture of possible dynamic behavior of **2** complete, we took under consideration form **2**-1, diastereomeric to **2**. The isomer **2**-1 was constructed from **2** by the tellurophene ring inversion. Energetic barrier of such an inversion in 21-iodo-21-methyl-21-platina-23-telluraporphyrin, i.e., a platinum(IV) analog of **2,** was high enough (12 kcal/mol) to observe separate diastereomers at low temperature ^{1}H NMR spectrum.[7] Assuming, that inversion barriers are of the same order and taking into account that the low temperature ^{1}H NMR spectrum of **2** presents only one set of signals, we presume, that **2**-1 which is 6.3 kcal/mole (DFT) less thermodynamically stable than **2** is not present in solution in detectable concentration.

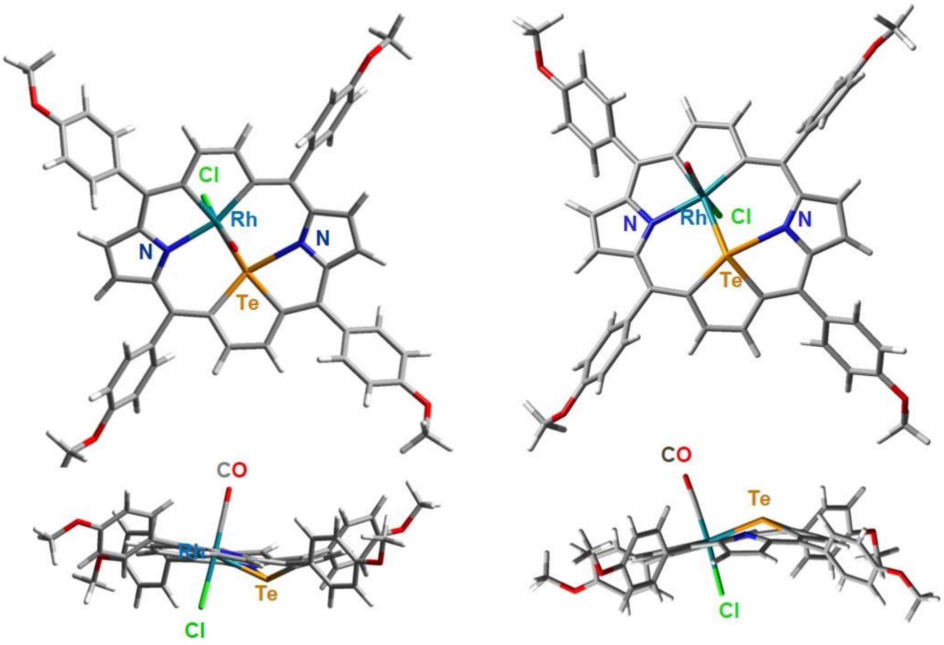

2
0 kcal/mol

2-1
6.3 kcal/mol

Figure S45. DFT-optimized structure of **2** and its isomer **2**-1.

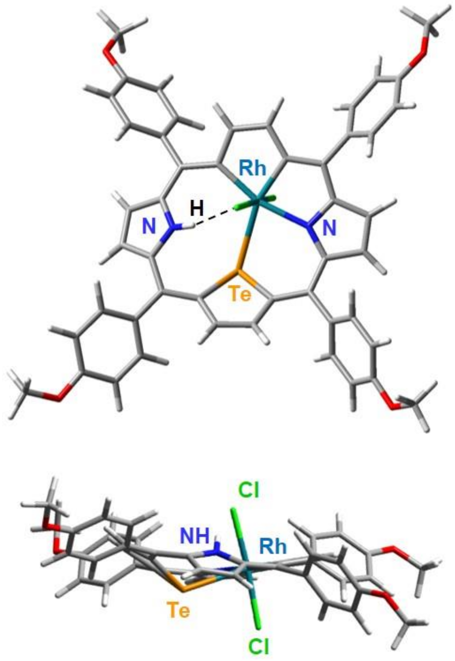

Figure S46. DFT-optimized structure of $\mathbf{2_A}$**-H**.

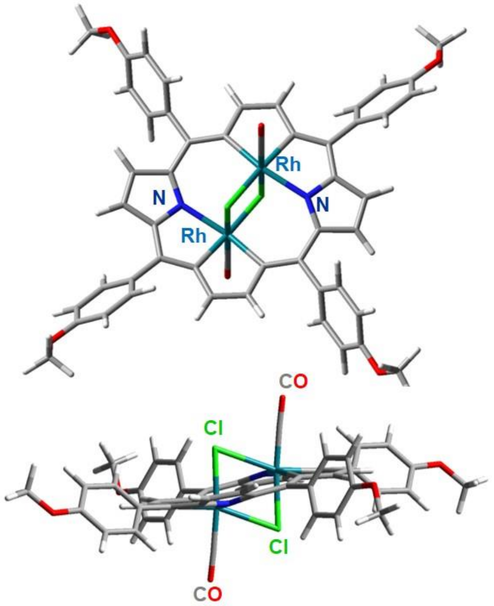

Figure S47. DFT-optimized structure of $\mathbf{3_A}$.

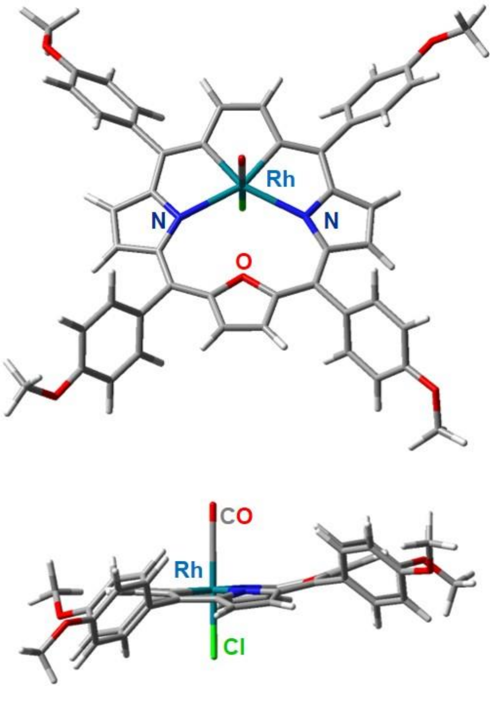

Figure S48. DFT-optimized structure of $\mathbf{4_A}$.

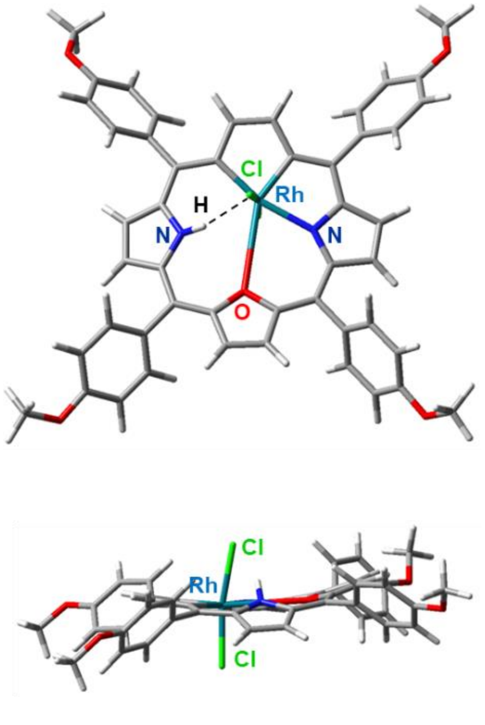

Figure S49. DFT-optimized structure of $\mathbf{4_A}$**-H**.

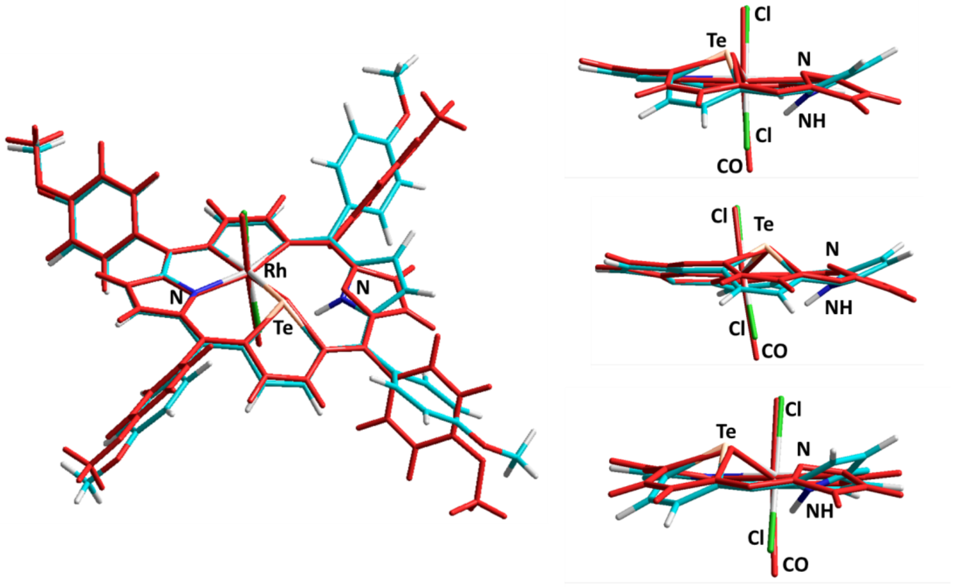

Figure S50. Comparison of **2** (red) and **2-H** (blue-white) geometries (DFT structures) in several projections; in side view aryl rings omitted for clarity.

Calculations for 8

The structure of the intermediate product **8** was proposed on the basis of ^{1}H NMR analysis (C_1 symmetry, low aromaticity, dynamic behavior) and by analogy to literature examples, where coordination of Rh^{I}Cl(CO) unit to a bidentate site of a porphyrin can be regarded as typical.[8-10]
Striking similarity of ^{1}H NMR characteristics of **8** and similar side-on palladium(II) complex with 21,23-ditelluraporphyrin (with two Cl ligands), supports this hypothesis.[11]
There are two possibile arrangements of CO and Cl ligands at rhodium in such a complex, thus, two versions (**8** and **8-1**, Figure S51) were considered. We have chosen the form **8** which is lower by 7.5 kcal/mol in energy than **8-1**.
The realiability of structure **8** was confirmed by agreement of calculated and experimental chemical shifts (Figure S55).

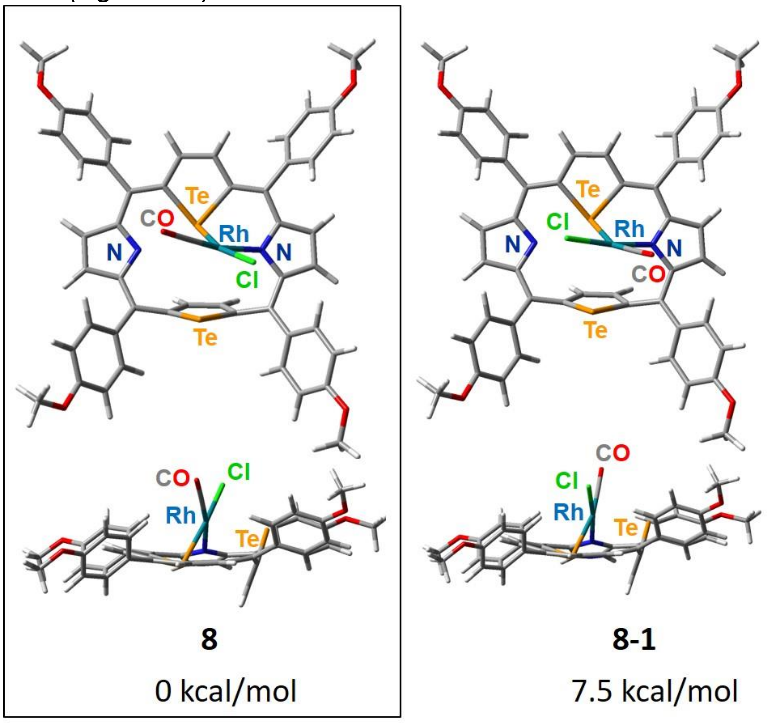

Figure S51. DFT-optimized structures of isomers of **8**.

Calculations for intermediates [9] and 9

The calculated structure [9] is proposed on a basis of the following assumptions: (1) it is a product obtained directly from **8** by the rhodium(I) insertion to Te–C bond of one tellurophene ring, thus a six-membered $RhTeC_4$ ring is formed; (2) the insertion proceeds via an oxidative addition, thus formation of a rhodium(III) species with octahedal coordination geometry is claimed; (3) in the literature, analogous metal insertion into Te–C bond was documented for benzo[*b*]tellurophene.[12]

Two possible isomers, with different CO and Cl positions, were taken under consideration (**[9]** and **[9-1]**), showing significantly different energies (Figure S52). The lower in energy isomer, **[9]**, was chosen for further discussion.

Spectroscopic observations:

A reactive compound, which is a good candidate for the sought species containing the six-membered $RhTeC_4$ ring, was observed in the ^{1}H NMR studies (spectra in Figures 35 and 36). The observed compound is numbered **9** and shows the following spectroscopic characteristics: (1) the number of β-H signals indicates C_1 symmetry; (2) ^{1}H NMR spectral range indicates very low macrocyclic, thus indicating one ring (tellurophene or $RhTeC_4$) significant tilt blocking the π-electron conjugation; (3) characteristic ABX pattern with large AB coupling constant (J = 10.8 Hz), consistent with a very large six-membered ring presence; where X = ^{103}Rh, coupled to one of protons with small J = 1.7 Hz. Coupling constants ($^3J_{HH}$) in the range 9.7–11.1 Hz were observed for CH=CH unit of $MTeC_4$ ring (M = Ru, Os, Fe)[12] and the value of 12 Hz was reported for similar $PtSC_4$ ring.[13]

Hypothesis: The proton chemical shifts measured for the detected compound **9** (values δ_{exp}, in black, Figure S52) and the calculated values for **[9]** (values δ_{calc}, in red), are in a similar δ range, however the $\delta_{exp}/\delta_{calc}$ accordance of the diagnostic tellurophene and rhodatelluracycle β-hydrogen chemical shifts is not acceptable. We may put forward the hypothesis, that the compound **9** detected in solution is a product of transformation of **[9]**, preserving the following structural features: the six-membered ring, the strong tellurophene tilt and low macrocyclic aromaticity. A good candidate for **9** is presented in Figure 52. The hypothesis is supported by accordance of the calculated (values δ_{calc}, in blue) and experimental (values δ_{exp}, in black) proton chemical shifts, and the fact, that the formation of **9** in the **1**➞**2** transformation, was not always reproducible. This suggests, that another, faster path may occur (direct **[9]** ➞ **2** reaction). The molecule **9** contains rhodium(I) center in a typical square planar coordination environment. The transformation of **[9]** to **9**, required a reductive elimination step, which was accomplished in an atypical fashion (*intramolecularly*): two formally negative ligands ((Te21 and chloride) formed the new covalent bond Te–Cl and did not leave the molecule, but formed one neutral Te21(Cl)-donor in **9**. The lowering of the rhodium(I) coordination number to four was accomplished by CO dissociation (besides elimination of Cl).

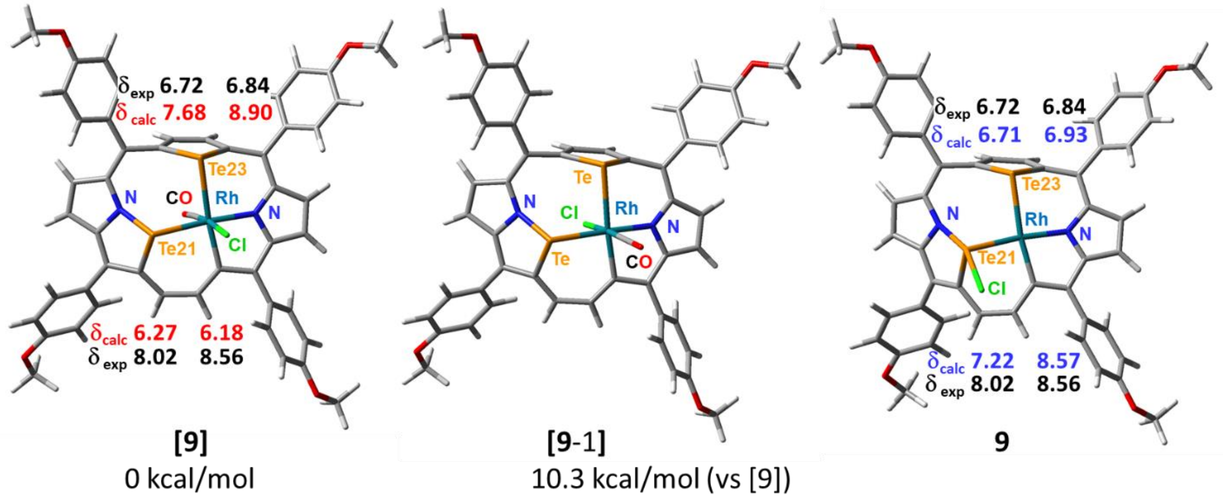

[9] 0 kcal/mol | **[9**-1**]** 10.3 kcal/mol (vs [9]) | **9**

Figure S52. DFT-optimized structure of **[9]**, **[9**-1**]** and **9** and diagnostic δ values.

Calculations for 10

The proposed structure of a side-on complex of **2** (rhodium(III) compound) with Rh^{I}Cl(CO) unit attached to two among three available donors on one face of 21-rhoda-23-telluraporphyrin (Cl, N24 and Te). Thus, three possible pairs of donors were considered to bind the Rh(I) center: (1) Te and Cl; (2) N and Cl; and (3) Te and N. The third possibility gave a structure **10**-2 (Figure 53) strained angles around rhodium(I) (∠NRhTe = 61°) and with the highest energy. The second choice of donors (2) allows for construction of four isomers differing by positions of CO and Cl ligands on Rh(I) and by tellurophene ring tilt (tellurium up or tellurium down) and structure **10**-1, the isomer with the lowest energy, is shown in Figure S53 (for other isomers, which are not shown, the energy equals to 5.0–5.5, kcal/mol in comparision with **10**). In the isomer with the lowest energy (**10**) the Rh(I) center is attached to the macrocycle through Te and Cl donors, and this isomer is chosen for the discussion in the main text. If Cl and CO ligands on Rh(I) center in **10** are swapped, higher energy isomer is obtained (4.9 kcal/mol, not shown). The choice between **10** and **10**-1 not much differing in energy, was based on the assumption, that the next reaction step of the **2** to **3** transformation will by rhodium insertion into Te-C bond.

The realiability of structures **10** and **10**-1 was confirmed by agreement of calculated and experimental chemical shifts (Figure S55).

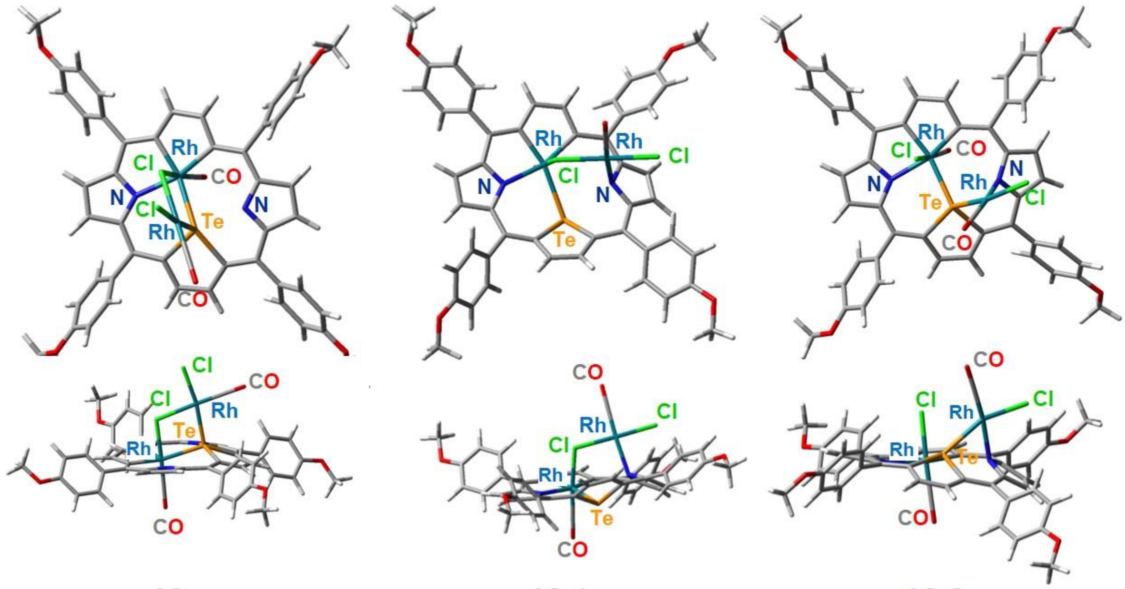

10
0 kcal/mol

10-1
1.3 kcal/mol

10-2
10.5 kcal/mol

Figure S53. DFT-optimized structure of **10** and its chosen isomers.

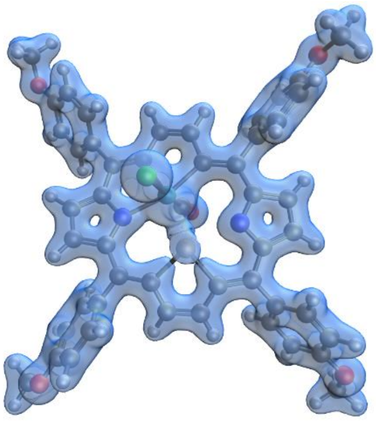

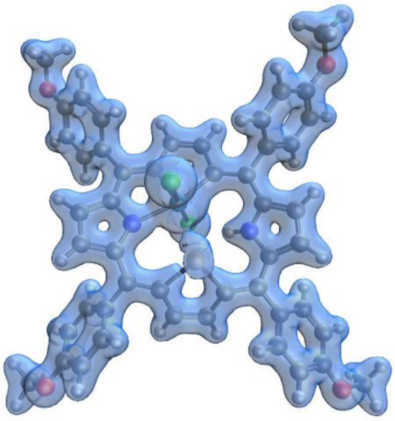

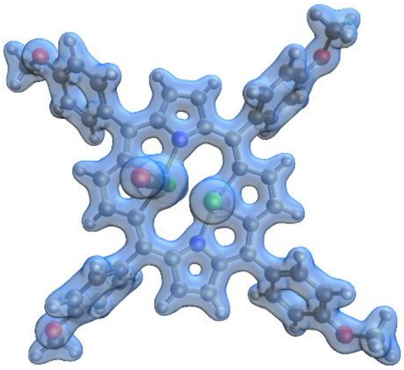

2_A 2_A-H 3_A

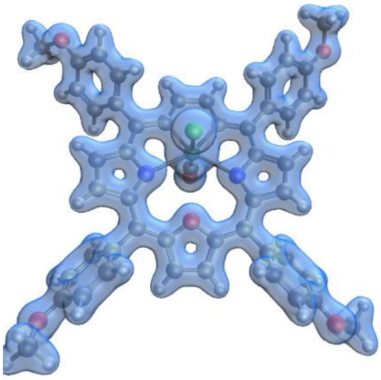

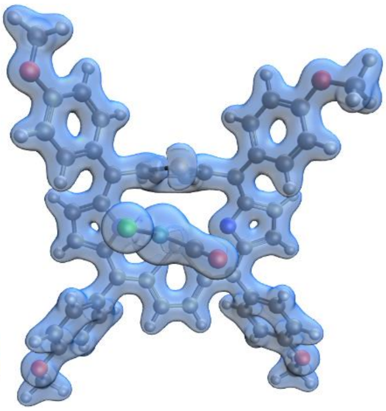

4_A 8

Figure S54. Total electron density $\mathbf{2_A}$, $\mathbf{2_A}$**-H**, $\mathbf{3_A}$, $\mathbf{4_A}$ and **8** (percentage 80%).

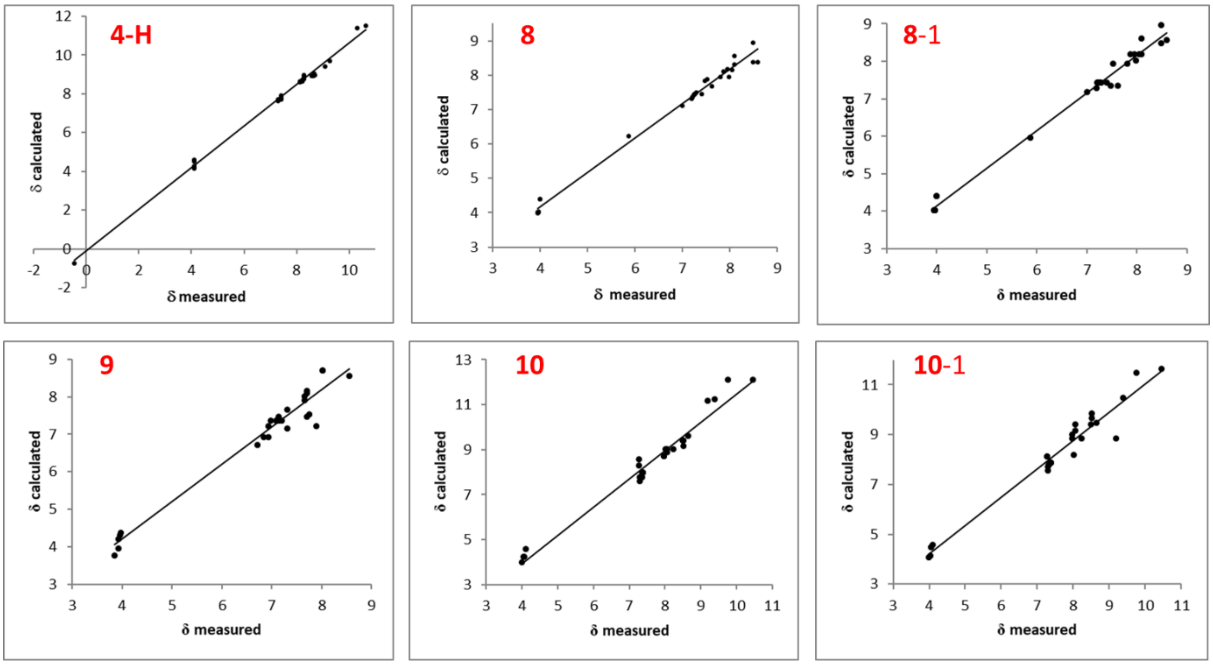

Figure S55. Calculated vs experimental ^{1}H NMR shifts for $\mathbf{4_A}$**-H, 8**, **8**-1**, 9**, **10** and **10**-1.

Table S4. Cartesian coordinates for **2**$_A$, **2**$_A$**-H**, **3**$_A$, **4**$_A$, **4**$_A$**-H**, **8**, **8**-1, **[9]**, **9**, **10** and **10**-1 (B3PW91/SDD for Rh and Te, and 6-31G(d,p) for the rest of atoms).

Cartesian coordinates for **2**$_A$:

Center Number	Atomic Number	Coordinates (Angstroms) X	Y	Z
1	8	3.319206	13.816719	5.322946
2	8	8.564039	4.132417	12.913878
3	7	2.356292	8.145226	10.462558
4	6	5.118768	4.448344	11.634369
5	1	4.360802	3.860303	11.125015
6	6	-0.316867	5.174030	12.290606
7	6	3.968308	9.131556	9.188123
8	1	4.415055	9.751676	8.423358
9	6	2.331798	5.897128	11.970003
10	6	2.894099	12.849092	6.176514
11	6	3.550819	7.594025	10.774014
12	6	4.594292	8.225416	9.993294
13	1	5.646638	7.976590	10.017834
14	6	2.839798	12.981301	7.567252
15	1	3.141958	13.901369	8.054918
16	6	1.511236	9.583466	8.637294
17	6	2.536436	9.023573	9.455290
18	6	2.387941	11.914287	8.344033
19	1	2.346663	12.024749	9.424226
20	6	1.971128	10.710466	7.766983
21	6	2.030933	4.697362	12.632117
22	1	2.825776	4.038485	12.971776
23	6	3.573624	6.436443	11.590833
24	6	4.862861	5.792968	11.927561
25	6	7.341729	4.607725	12.564801
26	6	0.693182	4.318155	12.770628
27	1	0.450257	3.334119	13.164437
28	6	6.340787	3.853426	11.944155
29	1	6.503034	2.812636	11.687223
30	6	3.734291	15.050404	5.872327
31	1	4.028751	15.674173	5.026900
32	1	4.593737	14.927207	6.544248
33	1	2.920944	15.545868	6.418333
34	6	2.038083	10.596615	6.367656
35	1	1.739878	9.664665	5.895049
36	6	7.104199	5.957160	12.859891
37	1	7.890200	6.528024	13.344174
38	6	2.493401	11.644546	5.582117
39	1	2.553987	11.554212	4.502010
40	6	5.886673	6.536987	12.541839
41	1	5.710154	7.580692	12.786419
42	6	8.854128	2.776640	12.639911
43	1	8.815301	2.562931	11.563875
44	1	8.167084	2.100803	13.165485
45	1	9.869279	2.606012	13.001291
46	8	-3.414848	0.457049	15.345451
47	8	-8.218706	8.251637	5.475390
48	7	-2.332866	6.268027	10.245585
49	6	-5.259528	8.963797	7.559887
50	1	-4.764585	9.793184	8.056683
51	6	0.187261	9.104003	8.542891
52	6	-4.023494	5.101999	11.238927
53	1	-4.533303	4.473535	11.955108
54	6	-2.302889	8.095744	8.271196
55	6	-3.026715	1.455715	14.513147
56	6	-3.500291	6.512172	9.577185
57	6	-4.576182	5.787920	10.199072
58	1	-5.614831	5.825180	9.901507
59	6	-3.174683	1.436828	13.122656
60	1	-3.614636	0.584009	12.617899
61	6	-1.671093	4.803812	12.161632
62	6	-2.600036	5.398795	11.262924
63	6	-2.742039	2.526810	12.368273
64	1	-2.849874	2.497594	11.287683
65	6	-2.153028	3.647581	12.966161
66	6	-1.978404	9.177381	7.443570
67	1	-2.692021	9.624238	6.753710
68	6	-3.512518	7.428460	8.495315
69	6	-4.742388	7.671542	7.710607
70	6	-7.086472	8.134928	6.215446
71	6	-0.705501	9.707309	7.610908
72	1	-0.413136	10.592344	7.050191
73	6	-6.418572	9.202791	6.823399
74	1	-6.794626	10.216548	6.742847
75	6	-4.025882	-0.683819	14.777345
76	1	-4.263151	-1.346076	15.611213
77	1	-4.952181	-0.426005	14.247873
78	1	-3.350048	-1.204205	14.086354
79	6	-2.001627	3.638947	14.365214
80	1	-1.550328	4.498171	14.853162
81	6	-6.584168	6.834275	6.360554
82	1	-7.113058	6.017935	5.878670
83	6	-2.435912	2.568573	15.129054
84	1	-2.334579	2.569716	16.209756
85	6	-5.433022	6.610073	7.098510
86	1	-5.045660	5.599208	7.192794
87	6	-8.765921	9.542320	5.296721
88	1	-9.059111	9.994629	6.252976
89	1	-8.064732	10.212520	4.782746
90	1	-9.654032	9.410747	4.676771
91	45	-0.823314	7.582988	9.513200
92	17	-1.768729	9.271954	10.937255
93	8	0.461264	5.615593	7.644906
94	6	-0.033938	6.359999	8.368652
95	52	0.577631	7.035420	11.703481

Cartesian coordinates for **2**$_A$**-H**:

Center Number	Atomic Number	Coordinates (Angstroms) X	Y	Z
1	8	3.384028	13.826416	5.446416
2	8	8.376688	3.706929	13.347243
3	7	2.620742	7.368208	9.613276
4	6	5.981408	5.250336	11.012853

5	1	5.903897	5.555858	9.973531
6	6	-0.311300	5.076748	12.337327
7	6	4.036493	9.087522	9.460439
8	1	4.423041	10.068071	9.221966
9	6	2.351113	5.579756	11.801149
10	6	2.926585	12.763739	6.156642
11	6	3.686373	7.069409	10.424532
12	6	4.611851	8.143434	10.287705
13	1	5.534067	8.234186	10.844579
14	6	1.836736	12.800825	7.032953
15	1	1.287438	13.719708	7.204883
16	6	1.732406	9.218688	8.289115
17	6	2.759896	8.596869	9.046532
18	6	1.454573	11.643869	7.708802
19	1	0.619863	11.680370	8.403479
20	6	2.139637	10.433088	7.537063
21	6	1.984511	4.320919	12.294149
22	1	2.718263	3.520130	12.345357
23	6	3.624101	6.001586	11.356259
24	6	4.865965	5.397073	11.853127
25	6	7.272154	4.263687	12.797240
26	6	0.641017	4.069226	12.569902
27	1	0.310048	3.069096	12.834608
28	6	7.172286	4.693326	11.468825
29	1	8.004444	4.585841	10.782328
30	6	2.710707	15.060830	5.589032
31	1	3.228703	15.764678	4.935796
32	1	2.754531	15.430009	6.621845
33	1	1.660377	14.987982	5.278597
34	6	3.238486	10.420447	6.657605
35	1	3.779623	9.492089	6.496435
36	6	6.168404	4.403510	13.653364
37	1	6.269595	4.079501	14.684287
38	6	3.624629	11.561706	5.973086
39	1	4.461642	11.548974	5.281944
40	6	4.990903	4.958749	13.188015
41	1	4.154141	5.085947	13.868210
42	6	9.524582	3.552013	12.534627
43	1	9.891667	4.518189	12.166004
44	1	9.329026	2.890403	11.681314
45	1	10.285258	3.099069	13.171750
46	8	-3.610468	0.533974	15.408433
47	8	-8.094314	8.496292	5.656942
48	7	-2.290173	6.450660	10.486239
49	6	-5.014403	9.142570	7.581397
50	1	-4.416654	9.969417	7.954134
51	6	0.388422	8.772272	8.300647
52	6	-4.059575	5.451922	11.558510
53	1	-4.594196	4.885643	12.307937
54	6	-2.139797	8.042613	8.343290
55	6	-3.195577	1.539343	14.599164
56	6	-3.455910	6.772001	9.853046
57	6	-4.581457	6.176276	10.535764
58	1	-5.621193	6.312303	10.272887
59	6	-3.495760	1.640888	13.236601
60	1	-4.086979	0.882642	12.735644
61	6	-1.707887	4.871533	12.315420
62	6	-2.608456	5.604614	11.506564
63	6	-3.016869	2.724961	12.503376
64	1	-3.236354	2.784784	11.441409
65	6	-2.234078	3.724220	13.096625
66	6	-1.783955	8.876833	7.268721
67	1	-2.495263	9.206608	6.514953
68	6	-3.416592	7.562378	8.686741
69	6	-4.633590	7.831531	7.893410
70	6	-6.958568	8.352556	6.387009
71	6	-0.457678	9.260382	7.256928
72	1	-0.069098	9.891156	6.460064
73	6	-6.163245	9.410494	6.838999
74	1	-6.431390	10.440384	6.631486
75	6	-4.414842	-0.485111	14.847987
76	1	-4.638989	-1.174707	15.663003
77	1	-5.354444	-0.084225	14.446797
78	1	-3.886357	-1.027203	14.053352
79	6	-1.929576	3.592714	14.464901
80	1	-1.324870	4.355004	14.948021
81	6	-6.591486	7.034090	6.690365
82	1	-7.215509	6.225195	6.323471
83	6	-2.406214	2.526336	15.207941
84	1	-2.186891	2.434076	16.266942
85	6	-5.449359	6.781697	7.432832
86	1	-5.163623	5.755174	7.644774
87	6	-8.504680	9.804924	5.316816
88	1	-8.718672	10.407912	6.208844
89	1	-7.751284	10.320734	4.707398
90	1	-9.420054	9.692650	4.733778
91	45	-0.689137	7.568561	9.549805
92	52	0.662719	6.873594	11.887615
93	17	0.128922	5.666565	8.380852
94	17	-1.290299	9.482893	10.830198
95	1	1.901776	6.701993	9.305503

Cartesian coordinates for $\mathbf{3_A}$:

Center Number	Atomic Number	Coordinates (Angstroms) X	Y	Z
1	45	1.261416	0.372074	-0.620502
2	17	1.128352	0.323779	1.786241
3	8	-1.028117	-8.850061	0.268124
4	8	9.567808	-0.777692	0.115174
5	7	1.794167	-1.671416	-0.355708
6	8	1.674963	0.687781	-3.573462
7	6	6.070204	0.162809	0.814652
8	1	5.488838	0.769914	1.502536
9	6	1.191361	2.401848	-0.396652
10	6	2.380912	-3.801177	0.205105
11	1	2.268243	-4.835891	0.495585
12	6	3.201786	0.623315	-0.379539
13	6	-0.777053	-7.519032	0.172325
14	6	3.159043	-1.753476	-0.249076
15	6	3.541988	-3.100124	0.078273
16	1	4.551973	-3.445542	0.249160
17	6	-0.256263	-6.886041	-0.960647
18	1	-0.023300	-7.447368	-1.858612
19	6	-0.090297	-3.257304	0.183450
20	6	1.272893	-2.882402	-0.025545
21	6	-0.035056	-5.509064	-0.940362
22	1	0.365428	-5.023323	-1.825967
23	6	-0.331093	-4.733602	0.186155
24	6	3.569253	1.972166	-0.346064
25	1	4.607935	2.286519	-0.266938
26	6	3.934136	-0.567926	-0.283019

27	6	5.406262	-0.602963	-0.150958
28	6	8.214858	-0.673579	0.084573
29	6	2.520883	2.892855	-0.334491
30	1	2.731221	3.951146	-0.196577
31	6	7.458234	0.134511	0.939209
32	1	7.932739	0.732641	1.709048
33	6	-0.741991	-9.662533	-0.851981
34	1	-1.010633	-10.680000	-0.563865
35	1	0.323790	-9.632576	-1.113389
36	1	-1.334443	-9.369022	-1.728327
37	6	-0.855594	-5.389836	1.313575
38	1	-1.080823	-4.812394	2.206004
39	6	7.565029	-1.449559	-0.885372
40	1	8.167684	-2.067048	-1.543994
41	6	1.493703	0.552536	-2.448744
42	6	-1.072366	-6.758869	1.312754
43	1	-1.465696	-7.267791	2.187212
44	6	6.184610	-1.413801	-0.997238
45	1	5.693792	-2.008077	-1.763059
46	6	10.272313	-0.013448	1.072794
47	1	9.989946	-0.287197	2.097638
48	1	10.110582	1.063001	0.931036
49	1	11.328913	-0.239070	0.921212
50	8	1.028102	8.850064	-0.268139
51	8	-9.567815	0.777686	-0.115134
52	7	-1.794169	1.671416	0.355657
53	6	-6.070218	-0.162819	-0.814640
54	1	-5.488859	-0.769930	-1.502525
55	6	-1.191362	-2.401845	0.396608
56	6	-2.380917	3.801173	-0.205163
57	1	-2.268250	4.835885	-0.495653
58	6	-3.201788	-0.623314	0.379522
59	6	0.777043	7.519034	-0.172348
60	6	-3.159046	1.753476	0.249042
61	6	-3.541992	3.100122	-0.078311
62	1	-4.551979	3.445540	-0.249190
63	6	0.256275	6.886032	0.960628
64	1	0.023327	7.447350	1.858602
65	6	0.090294	3.257305	-0.183500
66	6	-1.272896	2.882402	0.025493
67	6	0.035070	5.509054	0.940333
68	1	-0.365397	5.023305	1.825941
69	6	0.331088	4.733603	-0.186196
70	6	-3.569254	-1.972166	0.346056
71	1	-4.607938	-2.286520	0.266946
72	6	-3.934139	0.567927	0.283003
73	6	-5.406266	0.602963	0.150956
74	6	-8.214865	0.673574	-0.084547
75	6	-2.520885	-2.892856	0.334471
76	1	-2.731223	-3.951148	0.196571
77	6	-7.458249	-0.134523	-0.939183
78	1	-7.932762	-0.732660	-1.709012
79	6	0.741995	9.662526	0.851978
80	1	1.010630	10.679996	0.563867
81	1	-0.323781	9.632565	1.113405
82	1	1.334463	9.369008	1.728311
83	6	0.855567	5.389849	-1.313619
84	1	1.080779	4.812417	-2.206059
85	6	-7.565027	1.449563	0.885385
86	1	-8.167675	2.067058	1.544008
87	6	1.072337	6.758883	-1.312790
88	1	1.465648	7.267814	-2.187250
89	6	-6.184606	1.413808	0.997237
90	1	-5.693781	2.008090	1.763048
91	6	-10.272330	0.013432	-1.072739
92	1	-9.989974	0.287172	-2.097589
93	1	-10.110597	-1.063015	-0.930973
94	1	-11.328928	0.239055	-0.921148
95	45	-1.261416	-0.372073	0.620446
96	17	-1.128438	-0.323807	-1.786289
97	8	-1.674657	-0.687595	3.573477
98	6	-1.493701	-0.552514	2.448689

Cartesian coordinates for $\mathbf{4_A}$:

Center Number	Atomic Number	Coordinates (Angstroms) X	Y	Z
1	6	5.338121	3.738059	1.144867
2	6	5.387701	2.725299	0.163471
3	1	5.381826	2.955616	-0.899772
4	6	5.485798	1.434854	0.648467
5	1	5.563293	0.565830	-0.001381
6	6	5.521570	1.317969	2.054325
7	6	5.766795	0.161753	2.783554
8	6	5.719318	0.293480	4.188454
9	6	6.036075	-0.729316	5.155701
10	1	6.398117	-1.721224	4.925188
11	6	5.815789	-0.171771	6.369789
12	1	5.963991	-0.618161	7.342342
13	6	5.367301	1.196278	6.137042
14	6	4.978029	2.016772	7.209195
15	6	4.662154	3.381436	7.173639
16	6	4.111081	4.170711	8.200934
17	1	3.806109	3.803465	9.168969
18	6	4.025315	5.458873	7.719692
19	1	3.637055	6.330369	8.224288
20	6	4.523161	5.442380	6.402898
21	6	4.668696	6.522494	5.522209
22	6	4.990930	6.480810	4.155380
23	6	5.246619	7.709567	3.412804
24	1	5.295122	8.697591	3.847137
25	6	5.439367	7.358979	2.119290
26	1	5.676921	7.993159	1.277060
27	6	5.299277	5.923554	2.074282
28	6	5.390165	5.108415	0.925161
29	45	5.268681	3.045768	3.009623
30	7	5.352311	1.460675	4.793687
31	8	4.885567	4.167571	6.081942
32	7	5.063623	5.402931	3.313917
33	6	6.092492	-1.136148	2.145646
34	6	7.173699	-1.250453	1.264204
35	1	7.792903	-0.378461	1.073454
36	6	7.491544	-2.462843	0.652733
37	1	8.345114	-2.511320	-0.014158
38	8	6.933133	-4.824701	0.379012
39	6	6.716946	-3.596250	0.917483
40	6	5.630629	-3.499176	1.797971
41	1	5.035854	-4.387456	1.986826
42	6	5.327967	-2.288547	2.401134
43	1	4.475778	-2.223549	3.072181
44	6	8.015994	-4.975542	-0.515806
45	1	8.007414	-6.021175	-0.827498

46	1	7.903479	-4.335877	-1.400921
47	1	8.976219	-4.752663	-0.032631
48	6	4.849092	1.389687	8.558151
49	6	5.663045	1.797217	9.628973
50	1	6.410257	2.568544	9.464298
51	6	5.543272	1.218158	10.882651
52	1	6.180621	1.522077	11.707055
53	8	4.550267	-0.289566	12.367109
54	6	4.593174	0.211837	11.106736
55	6	3.770743	-0.204747	10.055112
56	1	3.023100	-0.976330	10.200683
57	6	3.907564	0.383807	8.798120
58	1	3.262392	0.058474	7.986813
59	6	3.618357	-1.315562	12.643953
60	1	3.756342	-1.573257	13.695034
61	1	2.585327	-0.977850	12.489472
62	1	3.800997	-2.205916	12.028527
63	6	4.415440	7.858515	6.139671
64	6	3.339370	8.658882	5.719363
65	1	2.683555	8.295042	4.933455
66	6	3.097364	9.896230	6.295273
67	1	2.262474	10.512436	5.976731
68	8	3.616659	11.598351	7.810716
69	6	3.933386	10.376043	7.313185
70	6	5.010128	9.594880	7.745028
71	1	5.678922	9.944334	8.523559
72	6	5.236359	8.349148	7.159929
73	1	6.080967	7.752676	7.493825
74	6	4.431418	12.131468	8.835285
75	1	4.006153	13.105008	9.083462
76	1	4.423666	11.497090	9.731151
77	1	5.467817	12.266850	8.500037
78	6	5.562343	5.699653	-0.423399
79	6	4.640147	6.617977	-0.937978
80	1	3.777173	6.896493	-0.339075
81	6	4.790415	7.170569	-2.209642
82	1	4.048012	7.871605	-2.574575
83	8	6.134981	7.280247	-4.245365
84	6	5.887783	6.806550	-2.996145
85	6	6.823168	5.891606	-2.494205
86	1	7.676007	5.630521	-3.112998
87	6	6.659955	5.348214	-1.229663
88	1	7.401904	4.657310	-0.839620
89	6	5.226803	8.212753	-4.793564
90	1	5.609883	8.461750	-5.784439
91	1	4.219407	7.787983	-4.894580
92	1	5.170573	9.128122	-4.189934
93	8	2.269744	2.802624	2.963325
94	6	3.415491	2.892274	2.971951
95	17	7.616941	3.245193	3.074764

Cartesian coordinates for **4$_{A}$-H**:

Center Number	Atomic Number	Coordinates (Angstroms) X	Y	Z
1	8	3.838436	13.515020	5.186279
2	8	8.123885	4.522408	13.871918
3	7	2.254550	7.326581	9.614210
4	6	5.697312	5.360360	11.227280
5	1	5.644573	5.492681	10.150434
6	6	0.008047	4.449903	11.859906
7	6	3.754143	9.005079	9.600979
8	1	4.198121	9.967048	9.389066
9	6	2.133167	5.049652	11.632169
10	6	3.257919	12.524768	5.911318
11	6	3.230436	7.002290	10.526092
12	6	4.177189	8.071594	10.515665
13	1	5.025320	8.141241	11.181522
14	6	2.078607	12.664877	6.650141
15	1	1.554796	13.613245	6.693463
16	6	1.675013	9.183636	8.112393
17	6	2.536121	8.538246	9.027451
18	6	1.572799	11.575405	7.357112
19	1	0.671972	11.697314	7.951749
20	6	2.218577	10.332053	7.349872
21	6	2.121925	3.800262	12.275491
22	1	3.001523	3.248193	12.570582
23	6	3.244626	5.862049	11.345192
24	6	4.530776	5.500828	11.989877
25	6	6.993612	4.842140	13.196498
26	6	0.804641	3.429153	12.420099
27	1	0.423196	2.520160	12.858424
28	6	6.918571	5.032695	11.812563
29	1	7.794625	4.920848	11.183782
30	6	3.206402	14.778737	5.161430
31	1	3.824382	15.413171	4.524224
32	1	3.148658	15.223475	6.163378
33	1	2.195826	14.718389	4.737089
34	6	3.408420	10.215825	6.606861
35	1	3.922505	9.259104	6.574423
36	6	5.836653	4.985822	13.976709
37	1	5.916393	4.851235	15.050757
38	6	3.920145	11.289105	5.895862
39	1	4.829993	11.196191	5.311111
40	6	4.629145	5.308155	13.380851
41	1	3.745882	5.437922	13.999505
42	6	9.323012	4.375474	13.136311
43	1	9.598173	5.304930	12.621721
44	1	9.249601	3.564238	12.400817
45	1	10.095410	4.128058	13.865716
46	8	-3.653329	0.401515	15.081731
47	8	-8.327964	9.063444	6.397022
48	7	-2.079670	6.246035	10.208718
49	6	-4.986572	9.334476	7.936427
50	1	-4.284529	10.084093	8.290187
51	6	0.334685	8.767839	8.024509
52	6	-3.757950	5.180603	11.368067
53	1	-4.226376	4.543228	12.103527
54	6	-2.139724	8.037888	8.225050
55	6	-3.140682	1.336552	14.242200
56	6	-3.301251	6.651631	9.749563
57	6	-4.365612	6.011607	10.488993
58	1	-5.423043	6.192608	10.357614
59	6	-3.224467	1.285943	12.847050
60	1	-3.718894	0.461900	12.345050
61	6	-1.400187	4.480107	11.850663
62	6	-2.310413	5.310171	11.167559
63	6	-2.660970	2.309603	12.084693
64	1	-2.727600	2.264584	11.001128
65	6	-2.005498	3.389236	12.682823
66	6	-1.863484	8.887659	7.130933
67	1	-2.635588	9.194972	6.429371

68	6	-3.380559	7.564002	8.678461
69	6	-4.668629	7.979025	8.087236
70	6	-7.121614	8.782258	6.953266
71	6	-0.543342	9.280387	7.026010
72	1	-0.199073	9.925949	6.220398
73	6	-6.197404	9.741592	7.378508
74	1	-6.410943	10.801136	7.292635
75	6	-4.330330	-0.702253	14.514862
76	1	-4.660751	-1.316860	15.353480
77	1	-5.206225	-0.385738	13.933949
78	1	-3.668888	-1.297339	13.871921
79	6	-1.925305	3.419986	14.084496
80	1	-1.425260	4.253329	14.570460
81	6	-6.817792	7.420905	7.094390
82	1	-7.541690	6.690049	6.747754
83	6	-2.487113	2.414351	14.856201
84	1	-2.437800	2.441286	15.940200
85	6	-5.612132	7.029394	7.653033
86	1	-5.378663	5.971655	7.735539
87	6	-8.680489	10.421020	6.226020
88	1	-8.731395	10.951155	7.185944
89	1	-7.976264	10.942832	5.565031
90	1	-9.669563	10.420748	5.765384
91	45	-0.605925	7.489442	9.221903
92	17	0.084638	5.667498	7.864597
93	17	-0.894725	9.170764	10.816264
94	1	1.651227	6.630685	9.159177
95	8	0.838157	5.419461	11.360674

Cartesian coordinates for **8**:

Center Number	Atomic Number	Coordinates (Angstroms) X	Y	Z
1	6	4.500086	3.699450	1.016695
2	6	5.589311	4.526889	1.242694
3	6	6.028990	5.404443	0.242762
4	6	3.814496	3.714296	-0.210867
5	6	4.263606	4.601526	-1.195316
6	6	5.357491	5.440870	-0.982929
7	1	6.113272	4.519907	2.193416
8	1	4.156564	3.039676	1.808595
9	1	3.756795	4.628159	-2.156525
10	1	5.677087	6.106751	-1.776887
11	6	-3.161179	4.871964	0.469860
12	6	-4.250685	5.663024	0.824228
13	6	-5.477542	5.485906	0.174695
14	6	-3.255537	3.894424	-0.533492
15	6	-4.510111	3.716286	-1.154310
16	6	-5.597976	4.500510	-0.817823
17	1	-4.138345	6.393647	1.617273
18	1	-2.226444	4.988966	1.009244
19	1	-4.611600	2.960886	-1.928334
20	1	-6.557784	4.374963	-1.308790
21	6	-3.644317	-4.319560	0.658563
22	6	-4.798615	-5.021850	0.949267
23	6	-6.009668	-4.683200	0.324901
24	6	-3.655679	-3.257508	-0.272087
25	6	-4.881521	-2.916843	-0.868240
26	6	-6.047079	-3.618842	-0.586571
27	1	-4.800287	-5.823678	1.680564
28	1	-2.724936	-4.542657	1.191004
29	1	-4.910353	-2.102082	-1.586166
30	1	-6.970491	-3.337435	-1.079790
31	6	3.506648	-4.758190	0.682680
32	6	4.563708	-5.654046	0.682322
33	6	5.627418	-5.491025	-0.216596
34	6	3.478789	-3.671473	-0.206953
35	6	4.545563	-3.521036	-1.097695
36	6	5.614421	-4.417045	-1.111723
37	1	4.594828	-6.487671	1.376801
38	1	2.693312	-4.880380	1.393060
39	1	4.539591	-2.693538	-1.802034
40	1	6.421434	-4.268321	-1.820304
41	6	3.866535	0.675134	0.032791
42	6	3.746018	-0.675669	0.175940
43	6	2.471426	-1.307663	-0.120021
44	6	2.717789	1.453397	-0.351319
45	1	4.814667	1.169964	0.226263
46	1	4.595649	-1.279110	0.486606
47	6	2.324732	-2.731599	-0.250739
48	6	0.531122	-4.601210	-0.838302
49	6	-0.835237	-4.518941	-0.829833
50	6	-1.187090	-3.134131	-0.615277
51	7	-0.016415	-2.394343	-0.480145
52	6	1.013492	-3.264115	-0.582352
53	1	1.142939	-5.476967	-1.006267
54	1	-1.537466	-5.319250	-1.023841
55	6	2.644501	2.833457	-0.458948
56	6	-0.349686	4.896971	-0.857305
57	6	1.003351	4.847714	-0.728659
58	6	1.369222	3.436793	-0.751957
59	7	0.289375	2.678543	-0.940654
60	6	-0.811965	3.516917	-0.952325
61	1	-0.975856	5.777555	-0.902035
62	1	1.687306	5.678025	-0.614801
63	6	-2.438239	-2.520436	-0.596070
64	6	-2.119990	3.041815	-0.912605
65	6	-1.836784	0.859778	-2.150831
66	6	-1.900704	-0.549467	-2.039582
67	6	-2.486298	-1.105942	-0.922993
68	6	-2.355747	1.625956	-1.133825
69	1	-1.336084	1.336569	-2.988790
70	1	-1.464618	-1.193111	-2.798530
71	52	-3.229206	0.395825	0.324854
72	52	1.053757	0.224578	-0.688375
73	45	1.104188	-2.051056	1.436395
74	6	-6.537827	7.200600	1.432931
75	1	-7.532461	7.646636	1.472633
76	1	-6.292889	6.778877	2.415917
77	1	-5.804434	7.976772	1.180157
78	6	-8.338396	-5.112174	0.110734
79	1	-9.047901	-5.821632	0.538412
80	1	-8.649321	-4.091738	0.367171
81	1	-8.330379	-5.224708	-0.980752
82	6	7.717645	-6.293142	-1.011768
83	1	8.383956	-7.125230	-0.779403
84	1	7.412854	-6.360312	-2.064329
85	1	8.253483	-5.348332	-0.852861
86	6	7.582788	7.077404	-0.413480
87	1	8.434392	7.587374	0.039645
88	1	7.917082	6.557878	-1.321174
89	1	6.822737	7.821336	-0.686484

90	8	7.103848	6.173527	0.560053
91	8	6.613803	-6.420234	-0.138917
92	8	-7.080739	-5.430095	0.676349
93	8	-6.599142	6.197860	0.436728
94	17	-0.763125	-2.305679	2.826523
95	6	2.211717	-1.979343	2.877474
96	8	2.914155	-1.953653	3.793766

Cartesian coordinates for **8**-1:

Center Number	Atomic Number	Coordinates (Angstroms) X	Y	Z
1	6	3.546334	4.874218	0.466475
2	6	4.614967	5.725820	0.694862
3	6	5.808076	5.572652	-0.024978
4	6	3.630090	3.838678	-0.483057
5	6	4.831790	3.695984	-1.186849
6	6	5.913861	4.548880	-0.970948
7	1	4.556386	6.515125	1.437709
8	1	2.637723	4.987304	1.050234
9	1	4.915660	2.912832	-1.935115
10	1	6.822080	4.410622	-1.546825
11	6	-3.063458	4.653023	0.572346
12	6	-4.079241	5.422927	1.130496
13	6	-5.398690	5.252949	0.694117
14	6	-3.328617	3.701330	-0.424161
15	6	-4.667765	3.532914	-0.835248
16	6	-5.684517	4.298433	-0.295547
17	1	-3.835914	6.130683	1.914654
18	1	-2.049122	4.758069	0.945509
19	1	-4.893697	2.804051	-1.608208
20	1	-6.712767	4.183524	-0.623322
21	6	-3.677245	-4.575914	0.323824
22	6	-4.796275	-5.314777	0.655253
23	6	-6.068000	-4.910588	0.216556
24	6	-3.776911	-3.409913	-0.470304
25	6	-5.061733	-3.022971	-0.897861
26	6	-6.195558	-3.752225	-0.561622
27	1	-4.722387	-6.201858	1.276165
28	1	-2.705128	-4.876933	0.701971
29	1	-5.159347	-2.142223	-1.525803
30	1	-7.165008	-3.423954	-0.919114
31	6	3.225384	-4.629220	0.513052
32	6	4.274677	-5.522015	0.652477
33	6	5.471826	-5.319266	-0.048818
34	6	3.333494	-3.499151	-0.320359
35	6	4.536811	-3.313635	-1.013393
36	6	5.597995	-4.207172	-0.888580
37	1	4.196378	-6.384823	1.306326
38	1	2.307387	-4.790920	1.070770
39	1	4.637144	-2.462775	-1.681445
40	1	6.506894	-4.032897	-1.453491
41	6	3.703581	0.816432	-0.083086
42	6	3.587773	-0.544648	0.020152
43	6	2.391780	-1.183119	-0.428867
44	6	2.611280	1.564534	-0.598069
45	1	4.589761	1.333261	0.276300
46	1	4.370961	-1.124818	0.501020
47	6	2.202234	-2.559791	-0.467418
48	6	0.546721	-4.402680	-1.180962
49	6	-0.824414	-4.442648	-1.237463
50	6	-1.295661	-3.151536	-0.832658
51	7	-0.217449	-2.381364	-0.473362
52	6	0.899284	-3.104058	-0.690303
53	1	1.243349	-5.170766	-1.489159
54	1	-1.445108	-5.233894	-1.638433
55	6	2.488593	2.924870	-0.729958
56	6	-0.600409	4.814671	-1.153540
57	6	0.756818	4.838288	-1.078218
58	6	1.182939	3.451241	-1.042158
59	7	0.142819	2.621285	-1.200440
60	6	-0.982530	3.415384	-1.197278
61	1	-1.274034	5.661275	-1.186922
62	1	1.398416	5.708659	-1.037323
63	6	-2.607486	-2.625636	-0.828168
64	6	-2.263647	2.882075	-1.010583
65	6	-2.705071	-1.237888	-1.158201
66	6	-2.500944	1.490831	-1.256236
67	52	1.019178	0.341983	-1.164483
68	45	-0.161813	-0.807416	0.900775
69	6	-6.235131	6.915912	2.171334
70	1	-7.210552	7.349383	2.396103
71	1	-5.824023	6.462488	3.081939
72	1	-5.559334	7.708432	1.825896
73	6	-8.409483	-5.321688	0.220196
74	1	-9.073800	-6.075341	0.644986
75	1	-8.676594	-4.336718	0.622992
76	1	-8.528529	-5.310622	-0.870617
77	6	7.670801	-6.085701	-0.521518
78	1	8.295947	-6.926048	-0.216089
79	1	7.545106	-6.109379	-1.611813
80	1	8.164995	-5.147702	-0.237226
81	6	8.020279	6.343133	-0.421443
82	1	8.660947	7.138128	-0.036723
83	1	8.500087	5.373466	-0.234788
84	1	7.892548	6.477260	-1.503626
85	8	6.793178	6.460274	0.268235
86	8	6.438366	-6.250502	0.150082
87	8	-7.099037	-5.699631	0.597170
88	8	-6.460721	5.948057	1.164024
89	52	-3.086273	0.222089	0.280765
90	6	-2.129685	0.752662	-2.373920
91	6	-2.228529	-0.644526	-2.320022
92	1	-1.783754	1.263392	-3.268772
93	1	-1.947770	-1.264712	-3.167766
94	17	-0.101026	1.146389	2.209684
95	6	-0.815052	-1.612782	2.430631
96	8	-1.260658	-2.000770	3.420421

Cartesian coordinates for **[9]**:

Center Number	Atomic Number	Coordinates (Angstroms) X	Y	Z
1	8	3.173916	12.960359	3.959434
2	8	7.719287	5.072558	15.019610
3	7	2.565775	7.587985	9.630357
4	6	5.827182	6.050876	12.005924
5	1	5.981776	6.197638	10.941544
6	6	-0.493120	4.729351	11.854130
7	6	4.212309	9.077654	9.182780
8	1	4.762591	9.874042	8.700771
9	6	2.081687	5.957110	11.943071

10	6	2.902239	12.065703	4.947205
11	6	3.521547	7.410287	10.593492
12	6	4.587806	8.359484	10.278136
13	1	5.479311	8.499636	10.873855
14	6	3.051944	12.318100	6.313402
15	1	3.397123	13.282235	6.670297
16	6	1.974190	8.995477	7.815136
17	6	2.903078	8.602121	8.786838
18	6	2.738032	11.320766	7.237849
19	1	2.834082	11.536089	8.298709
20	6	2.282227	10.060226	6.834378
21	6	3.390525	6.557871	11.689925
22	6	4.529709	6.175455	12.535007
23	6	6.732930	5.429664	14.156535
24	6	6.917756	5.697412	12.797102
25	1	7.895340	5.606565	12.336511
26	6	3.655158	14.233492	4.333788
27	1	3.815146	14.782682	3.404465
28	1	4.606203	14.164940	4.878423
29	1	2.928593	14.778082	4.951452
30	6	2.133096	9.830618	5.453765
31	1	1.789704	8.856761	5.116581
32	6	5.442137	5.507962	14.698452
33	1	5.308786	5.282800	15.752080
34	6	2.436844	10.812626	4.524961
35	1	2.331854	10.631238	3.459738
36	6	4.368729	5.867178	13.903102
37	1	3.380249	5.919020	14.347817
38	6	9.035083	4.970317	14.516024
39	1	9.389264	5.926469	14.108909
40	1	9.113465	4.199189	13.738519
41	1	9.662505	4.688065	15.362934
42	8	-2.845877	-0.593741	14.710051
43	8	-8.323291	10.300231	7.304171
44	7	-2.590313	5.612167	9.906905
45	6	-4.899577	9.457535	8.395196
46	1	-4.010831	9.938487	8.792879
47	6	0.691991	8.354709	7.874458
48	6	-4.230782	4.743284	11.182926
49	1	-4.712241	4.208675	11.990637
50	6	-2.441890	7.423702	7.960679
51	6	-2.653304	0.504709	13.934684
52	6	-3.793825	6.240581	9.532132
53	6	-4.806205	5.693196	10.380649
54	1	-5.834522	6.028071	10.406500
55	6	-3.355859	0.787913	12.759242
56	1	-4.108112	0.105431	12.379083
57	6	-1.811141	4.089685	11.707159
58	6	-2.803770	4.697790	10.933365
59	6	-3.064115	1.943396	12.039615
60	1	-3.584183	2.117231	11.102205
61	6	-2.095154	2.870105	12.469721
62	6	-1.830602	8.520621	7.397100
63	1	-2.511147	9.317252	7.092035
64	6	-3.741292	7.227960	8.553449
65	6	-4.916901	8.067475	8.231056
66	6	-7.182823	9.636287	7.625680
67	6	-0.448215	8.846494	7.251738
68	1	-0.279466	9.759089	6.678840
69	6	-6.013520	10.241435	8.095988
70	1	-5.960042	11.314046	8.245361
71	6	-3.835302	-1.520321	14.313143
72	1	-3.833462	-2.307736	15.068445
73	1	-4.831314	-1.059763	14.276282
74	1	-3.608493	-1.961905	13.333884
75	6	-1.384522	2.543480	13.646659
76	1	-0.613335	3.218283	14.004095
77	6	-7.219339	8.245218	7.463238
78	1	-8.132711	7.790119	7.093073
79	6	-1.656108	1.392295	14.363696
80	1	-1.108583	1.155578	15.270811
81	6	-6.102945	7.478206	7.758436
82	1	-6.137989	6.402659	7.608510
83	6	-8.334212	11.706384	7.441718
84	1	-8.168852	12.014066	8.482542
85	1	-7.579165	12.182486	6.802649
86	1	-9.326285	12.033307	7.126591
87	52	1.259075	4.436469	10.640031
88	6	-0.168559	5.627113	12.825661
89	6	1.134698	6.251511	12.871553
90	1	-0.929569	5.932866	13.541317
91	1	1.319243	7.044763	13.593457
92	45	0.799619	6.729264	9.136335
93	52	-1.323194	5.657525	8.280709
94	6	-0.077816	7.763591	10.402752
95	8	-0.603971	8.471025	11.135152
96	17	1.804113	5.553312	7.282211

Cartesian coordinates for **9**:

Center Number	Atomic Number	Coordinates (Angstroms) X	Y	Z
1	8	3.018404	12.986221	3.960784
2	8	7.498516	5.552900	15.115080
3	7	2.305546	7.582086	9.559012
4	6	5.623404	6.269314	12.023803
5	1	5.789419	6.369419	10.955971
6	6	-0.527509	4.555095	11.766041
7	6	3.948230	9.114777	9.116517
8	1	4.471928	9.922075	8.622928
9	6	1.897360	5.961653	11.886126
10	6	2.728676	12.051210	4.904880
11	6	3.278594	7.466368	10.532088
12	6	4.324705	8.442668	10.236215
13	1	5.190317	8.633695	10.854766
14	6	2.704201	12.291539	6.281735
15	1	2.913935	13.277516	6.681772
16	6	1.797458	8.851597	7.630198
17	6	2.677957	8.555316	8.699386
18	6	2.392443	11.251207	7.157623
19	1	2.355055	11.454973	8.224690
20	6	2.101908	9.961108	6.697182
21	6	3.173774	6.630979	11.636321
22	6	4.312978	6.356151	12.526121
23	6	6.515203	5.812047	14.219553
24	6	6.713729	6.009610	12.849283
25	1	7.702790	5.938707	12.410749
26	6	3.320557	14.294714	4.395270
27	1	3.523076	14.874621	3.493299
28	1	4.208377	14.314362	5.041286
29	1	2.478054	14.750094	4.932623
30	6	2.132668	9.743150	5.306340
31	1	1.924019	8.747886	4.923247

32	6	5.211847	5.857246	14.736608
33	1	5.070666	5.684676	15.798907
34	6	2.438689	10.767195	4.424158
35	1	2.467805	10.595617	3.352625
36	6	4.137376	6.111623	13.904324
37	1	3.138728	6.128720	14.326752
38	6	8.830209	5.487641	14.643278
39	1	9.145429	6.436147	14.190044
40	1	8.961549	4.678455	13.913621
41	1	9.450264	5.284803	15.517551
42	8	-2.979618	-0.610055	14.820753
43	8	-7.520229	10.873955	7.978241
44	7	-2.446594	5.247292	9.668640
45	6	-4.199065	9.349425	8.498668
46	1	-3.185176	9.625974	8.772689
47	6	0.635092	8.039531	7.607244
48	6	-4.175690	4.478307	10.958491
49	1	-4.698425	3.966303	11.755110
50	6	-2.281553	6.989751	7.620437
51	6	-2.748604	0.430878	13.990287
52	6	-3.568861	5.915733	9.332866
53	6	-4.662316	5.479314	10.163797
54	1	-5.662229	5.891269	10.164811
55	6	-3.398650	0.636631	12.766861
56	1	-4.133572	-0.070069	12.398370
57	6	-1.797723	3.844471	11.573212
58	6	-2.747855	4.381867	10.694502
59	6	-3.072473	1.742647	11.991911
60	1	-3.545651	1.861463	11.022334
61	6	-2.113905	2.688746	12.408145
62	6	-1.773720	8.074057	6.834966
63	1	-2.508739	8.659035	6.282030
64	6	-3.460573	6.947627	8.341324
65	6	-4.498933	8.006072	8.252520
66	6	-6.490018	9.994194	8.084695
67	6	-0.466638	8.481574	6.781943
68	1	-0.271496	9.322303	6.116935
69	6	-5.177176	10.340572	8.416604
70	1	-4.904125	11.370084	8.619642
71	6	-3.959522	-1.562242	14.449457
72	1	-3.989040	-2.293563	15.257984
73	1	-4.949255	-1.101029	14.343082
74	1	-3.693687	-2.069766	13.513864
75	6	-1.446823	2.435261	13.628617
76	1	-0.677541	3.122166	13.964342
77	6	-6.805952	8.652004	7.835313
78	1	-7.827382	8.400783	7.567142
79	6	-1.761492	1.339072	14.406949
80	1	-1.254305	1.154049	15.348491
81	6	-5.823707	7.677072	7.917661
82	1	-6.078764	6.643783	7.697859
83	6	-7.249990	12.241569	8.206254
84	1	-6.891348	12.420068	9.228503
85	1	-6.512098	12.633773	7.494263
86	1	-8.196787	12.764917	8.063926
87	52	1.214536	4.480625	10.534064
88	6	-0.289112	5.436700	12.777274
89	6	0.950820	6.167784	12.837954
90	1	-1.073474	5.652481	13.500471
91	1	1.074426	6.954807	13.578508
92	45	0.713325	6.498718	8.864823
93	52	-1.107978	5.222969	7.692893
94	17	-0.389572	5.376662	5.241528

Cartesian coordinates for **10**:

Center Number	Atomic Number	Coordinates (Angstroms) X	Y	Z
1	8	3.291547	13.759662	5.263739
2	8	8.612840	4.437446	13.280328
3	7	2.355600	8.004538	10.320511
4	6	5.232979	4.504689	11.810093
5	1	4.533706	3.851204	11.296239
6	6	-0.294079	5.024375	12.133968
7	6	3.970429	9.069549	9.124419
8	1	4.423549	9.711985	8.382414
9	6	2.373498	5.825615	11.929699
10	6	2.876462	12.774161	6.098428
11	6	3.558104	7.528261	10.707922
12	6	4.602808	8.205201	9.970419
13	1	5.664594	8.017851	10.053777
14	6	2.827130	12.878524	7.492250
15	1	3.123518	13.791065	7.996971
16	6	1.516431	9.456753	8.496174
17	6	2.534368	8.897927	9.324093
18	6	2.385870	11.794400	8.250123
19	1	2.344960	11.884855	9.332216
20	6	1.974550	10.600075	7.648614
21	6	2.054965	4.642383	12.605863
22	1	2.845000	4.014819	13.009406
23	6	3.601092	6.405474	11.576901
24	6	4.899595	5.849144	12.011854
25	6	7.391609	4.829053	12.842731
26	6	0.717986	4.224776	12.688737
27	1	0.490618	3.252134	13.118205
28	6	6.463648	3.991247	12.214725
29	1	6.690090	2.947862	12.026512
30	6	3.700047	14.987368	5.834455
31	1	3.986249	15.627808	4.998967
32	1	4.563016	14.857457	6.500103
33	1	2.884806	15.465900	6.391990
34	6	2.032550	10.513715	6.246973
35	1	1.735782	9.590359	5.756662
36	6	7.073306	6.178938	13.049113
37	1	7.801510	6.813492	13.544020
38	6	2.479337	11.579324	5.481131
39	1	2.535145	11.511577	4.399201
40	6	5.849638	6.678607	12.635829
41	1	5.607503	7.721905	12.817310
42	6	8.981569	3.082977	13.109758
43	1	9.014680	2.802738	12.049044
44	1	8.297848	2.407881	13.640127
45	1	9.981079	2.986957	13.535894
46	8	-3.437565	0.384292	15.238254
47	8	-8.391876	8.493526	5.715906
48	7	-2.340234	6.177388	10.117384
49	6	-5.308465	9.048997	7.659195
50	1	-4.762623	9.848464	8.151721
51	6	0.184297	8.997308	8.393498
52	6	-4.014948	5.023776	11.147287
53	1	-4.509393	4.388900	11.868187
54	6	-2.356085	8.071736	8.189632
55	6	-3.039648	1.367467	14.394754

56	6	-3.531453	6.471996	9.506880
57	6	-4.595122	5.753504	10.152984
58	1	-5.644874	5.826399	9.905977
59	6	-3.183715	1.330180	13.003665
60	1	-3.629829	0.474293	12.509793
61	6	-1.648673	4.667831	12.003528
62	6	-2.585615	5.288238	11.123813
63	6	-2.740733	2.406183	12.236047
64	1	-2.847281	2.363854	11.155551
65	6	-2.142871	3.528220	12.822474
66	6	-2.023455	9.164198	7.383846
67	1	-2.747165	9.657648	6.737762
68	6	-3.571790	7.428818	8.460123
69	6	-4.830770	7.735751	7.748946
70	6	-7.229645	8.318953	6.391042
71	6	-0.722023	9.646097	7.511251
72	1	-0.424641	10.538089	6.964626
73	6	-6.494884	9.346657	6.992148
74	1	-6.840318	10.373760	6.960478
75	6	-4.059859	-0.759054	14.686215
76	1	-4.304360	-1.405942	15.529804
77	1	-4.982983	-0.499185	14.152500
78	1	-3.388298	-1.296217	14.004090
79	6	-1.997169	3.541367	14.222448
80	1	-1.547815	4.407088	14.700802
81	6	-6.765026	6.998362	6.473851
82	1	-7.345333	6.214393	5.997672
83	6	-2.443626	2.484892	14.998085
84	1	-2.349576	2.502143	16.079194
85	6	-5.587267	6.714672	7.145328
86	1	-5.230771	5.689228	7.191086
87	6	-8.908985	9.805482	5.604946
88	1	-9.136758	10.235077	6.588738
89	1	-8.215918	10.469781	5.073123
90	1	-9.832174	9.717682	5.030559
91	45	-0.841181	7.473988	9.345809
92	17	-1.638227	9.170938	10.914221
93	8	0.251789	5.578435	7.300497
94	6	-0.172836	6.296845	8.089917
95	45	-0.270275	8.736864	12.983241
96	52	0.596562	6.848175	11.449333
97	17	-1.283188	10.507569	14.073547
98	6	0.710478	8.360050	14.438829
99	8	1.343142	8.109351	15.377557

Cartesian coordinates for **10**-1:

Center Number	Atomic Number	Coordinates (Angstroms) X	Y	Z
1	8	3.418266	13.408555	4.752869
2	8	7.986914	5.691581	14.640255
3	7	2.280782	7.863498	9.948755
4	6	5.018126	4.814325	12.648135
5	1	4.467346	3.942835	12.305546
6	6	-0.217024	4.314068	11.247828
7	6	3.974191	8.467590	8.527543
8	1	4.468666	8.886236	7.661643
9	6	2.265394	5.531355	11.501230
10	6	3.006543	12.440530	5.607845
11	6	3.453372	7.302347	10.376621
12	6	4.532232	7.702452	9.521247
13	1	5.556533	7.364066	9.602282
14	6	3.364002	12.366186	6.958647
15	1	4.004247	13.114660	7.411680
16	6	1.553399	9.244893	8.087037
17	6	2.566402	8.558895	8.815786
18	6	2.886184	11.320200	7.744213
19	1	3.150896	11.273759	8.798679
20	6	2.032244	10.341143	7.211808
21	6	1.783813	4.740795	12.552773
22	1	2.332966	4.691639	13.489278
23	6	3.434891	6.300817	11.389916
24	6	4.608873	6.093258	12.239238
25	6	6.881346	5.750611	13.861359
26	6	0.537430	4.129455	12.425705
27	1	0.092340	3.610722	13.269395
28	6	6.141458	4.634511	13.447689
29	1	6.439382	3.630851	13.729287
30	6	4.270413	14.421151	5.252813
31	1	4.476248	15.084046	4.411158
32	1	5.215899	14.006803	5.625048
33	1	3.789156	14.995217	6.054590
34	6	1.683049	10.433704	5.851282
35	1	1.045403	9.672648	5.409993
36	6	6.483654	7.035813	13.462160
37	1	7.061077	7.887563	13.807101
38	6	2.165595	11.460859	5.058061
39	1	1.913550	11.526385	4.004323
40	6	5.368318	7.206370	12.662477
41	1	5.038299	8.206313	12.387497
42	6	8.423623	4.425202	15.093398
43	1	8.699685	3.769313	14.257819
44	1	7.659060	3.928919	15.704513
45	1	9.305917	4.612949	15.706730
46	8	-3.606645	0.122907	14.709960
47	8	-8.627563	9.764832	7.113946
48	7	-2.302097	5.816767	9.553233
49	6	-5.267959	9.412965	8.594514
50	1	-4.569834	9.956039	9.225238
51	6	0.180736	8.903739	8.163592
52	6	-3.916957	4.336540	10.197555
53	1	-4.365948	3.456483	10.635089
54	6	-2.410085	8.185218	8.264983
55	6	-3.074407	0.996553	13.821093
56	6	-3.523418	6.260051	9.118421
57	6	-4.546068	5.329364	9.507121
58	1	-5.601913	5.426604	9.298117
59	6	-2.051545	0.689743	12.916809
60	1	-1.620068	-0.304006	12.875747
61	6	-1.582991	3.997715	11.113635
62	6	-2.505768	4.669968	10.269165
63	6	-1.580672	1.674305	12.050549
64	1	-0.787297	1.428289	11.350008
65	6	-2.117777	2.967246	12.045758
66	6	-2.127860	9.392498	7.625886
67	1	-2.904664	10.000585	7.165798
68	6	-3.623360	7.526553	8.488975
69	6	-4.930555	8.129655	8.148408
70	6	-7.410524	9.295633	7.486467
71	6	-0.786892	9.771944	7.607750
72	1	-0.496692	10.737802	7.200162
73	6	-6.492392	9.996951	8.274428
74	1	-6.719861	10.987716	8.651311

75	6	-3.097308	-1.196018	14.737808
76	1	-3.656640	-1.718620	15.514922
77	1	-3.248632	-1.708691	13.779230
78	1	-2.029391	-1.212219	14.990557
79	6	-3.139200	3.258095	12.968962
80	1	-3.549910	4.262767	13.008581
81	6	-7.085469	8.011111	7.027903
82	1	-7.804244	7.485462	6.407171
83	6	-3.609901	2.292480	13.843807
84	1	-4.388975	2.518675	14.564863
85	6	-5.866583	7.440103	7.356069
86	1	-5.620430	6.452869	6.974734
87	6	-9.003307	11.057821	7.545673
88	1	-9.046572	11.122706	8.640457
89	1	-8.317669	11.826536	7.166633
90	1	-9.999434	11.234464	7.137487
91	45	-0.783687	7.207800	8.912486
92	17	-0.874894	8.236545	11.062073
93	8	-0.482825	6.120301	6.132661
94	6	-0.596518	6.513238	7.206446
95	45	1.274988	9.284300	11.331301
96	52	0.972291	5.321843	9.848545
97	6	0.560686	10.536483	12.415245
98	8	0.112382	11.349239	13.108199
99	17	3.370668	10.318917	11.633668

Cartesian coordinates for **10**-2:

Center Number	Atomic Number	Coordinates (Angstroms) X	Y	Z
1	8	3.292422	13.997527	5.547082
2	8	8.560233	4.181456	12.817241
3	7	2.357740	8.293365	10.552100
4	6	5.079697	4.449081	11.635323
5	1	4.307446	3.842431	11.171002
6	6	-0.368120	5.173536	12.319957
7	6	3.883508	8.986667	9.012755
8	1	4.288222	9.479604	8.139915
9	6	2.309036	5.894060	11.948828
10	6	2.870183	12.987734	6.347486
11	6	3.531773	7.614467	10.758830
12	6	4.515399	8.080208	9.824280
13	1	5.524773	7.703394	9.732805
14	6	2.958806	12.987431	7.743657
15	1	3.383083	13.828801	8.279687
16	6	1.455528	9.638202	8.652591
17	6	2.503592	9.055533	9.433046
18	6	2.497228	11.888988	8.465487
19	1	2.572247	11.891015	9.550890
20	6	1.920181	10.784505	7.822076
21	6	2.003739	4.720153	12.640164
22	1	2.793607	4.066839	13.000397
23	6	3.546524	6.448794	11.565389
24	6	4.837678	5.806381	11.880627
25	6	7.330373	4.644438	12.487845
26	6	0.658118	4.342465	12.791115
27	1	0.420312	3.354650	13.178737
28	6	6.309992	3.865786	11.929516
29	1	6.465220	2.815711	11.709029
30	6	3.862164	15.138576	6.159342
31	1	4.126335	15.816247	5.346214
32	1	4.767314	14.884624	6.725379
33	1	3.148883	15.637688	6.827302
34	6	1.850718	10.797342	6.417003
35	1	1.445925	9.935938	5.892531
36	6	7.104498	6.006384	12.734267
37	1	7.905156	6.594344	13.171440
38	6	2.320093	11.878202	5.688135
39	1	2.281524	11.886636	4.603366
40	6	5.880337	6.577308	12.430170
41	1	5.708156	7.629533	12.639257
42	6	8.841675	2.813216	12.597651
43	1	8.771885	2.551111	11.534106
44	1	8.169684	2.164483	13.173963
45	1	9.866644	2.658339	12.937183
46	8	-3.493241	0.588443	15.488617
47	8	-8.208873	8.108808	5.351564
48	7	-2.330306	6.183362	10.149750
49	6	-5.295567	8.855400	7.485439
50	1	-4.834536	9.685838	8.012191
51	6	0.136022	9.142676	8.555309
52	6	-4.036262	5.035488	11.144329
53	1	-4.557485	4.427626	11.869765
54	6	-2.323151	8.064162	8.210682
55	6	-3.097079	1.550439	14.621174
56	6	-3.489834	6.410399	9.460668
57	6	-4.571133	5.686675	10.074938
58	1	-5.604248	5.712320	9.757959
59	6	-3.224971	1.471396	13.230254
60	1	-3.653920	0.595592	12.756552
61	6	-1.718485	4.797362	12.150666
62	6	-2.615248	5.345217	11.192431
63	6	-2.785342	2.529175	12.436075
64	1	-2.875142	2.452121	11.356206
65	6	-2.209763	3.677043	12.995534
66	6	-2.024839	9.195090	7.440899
67	1	-2.745708	9.648502	6.763585
68	6	-3.511476	7.346214	8.395262
69	6	-4.739343	7.575884	7.604509
70	6	-7.082066	8.007190	6.099069
71	6	-0.776038	9.765863	7.655827
72	1	-0.518085	10.697527	7.158044
73	6	-6.454422	9.078098	6.744317
74	1	-6.862630	10.080983	6.689549
75	6	-4.096597	-0.578309	14.964143
76	1	-4.343612	-1.202153	15.824121
77	1	-5.016143	-0.344753	14.412556
78	1	-3.411009	-1.127754	14.306357
79	6	-2.075891	3.729157	14.396079
80	1	-1.636456	4.610567	14.854465
81	6	-6.540072	6.718989	6.213134
82	1	-7.038237	5.900472	5.703194
83	6	-2.517734	2.690900	15.197698
84	1	-2.432390	2.738850	16.278587
85	6	-5.390056	6.510152	6.956723
86	1	-4.972162	5.509475	7.026950
87	6	-8.797703	9.385983	5.203057
88	1	-9.115040	9.800240	6.168563
89	1	-8.114106	10.092592	4.715250
90	1	-9.674584	9.241870	4.570342
91	45	-0.845239	7.548340	9.443092
92	17	-1.866951	9.138360	10.908448
93	8	0.481425	5.707282	7.470711

94	6	-0.027516	6.399558	8.234108
95	45	1.605798	9.195284	12.482765
96	52	0.521305	6.977041	11.624444
97	8	0.417598	10.057197	15.051950
98	6	0.888517	9.731198	14.046200
99	17	3.069676	10.987813	12.411808

Table S5. Transition states cartesian coordinates: **2_A‡, 3_A‡** (B3PW91/SDD for Rh and Te, and 6-31G(d,p) for the rest of atoms):

Cartesian coordinates for **2_A**‡:

Center Number	Atomic Number	Coordinates (Angstroms) X	Y	Z
1	8	6.629524	-6.359987	0.758253
2	8	5.946431	7.048180	0.620972
3	7	2.354804	0.126639	-0.234588
4	6	4.357595	3.748211	1.043636
5	1	4.357687	2.900763	1.722681
6	6	-1.494947	2.736943	-0.548917
7	6	4.592461	-0.415772	-0.107321
8	1	5.478891	-1.027693	-0.014229
9	6	1.225992	2.867598	-0.548192
10	6	5.739795	-5.356940	0.537406
11	6	3.105943	1.276707	-0.290996
12	6	4.516210	0.937476	-0.226033
13	1	5.332842	1.642168	-0.300003
14	6	5.647270	-4.621229	-0.648160
15	1	6.300645	-4.828634	-1.488289
16	6	2.799092	-2.233866	0.144102
17	6	3.227458	-0.895969	-0.091714
18	6	4.692308	-3.611109	-0.761365
19	1	4.615551	-3.055230	-1.691770
20	6	3.816136	-3.310532	0.287331
21	6	0.506369	3.958342	-0.020345
22	1	1.028908	4.761686	0.491374
23	6	2.598398	2.594991	-0.344539
24	6	3.498391	3.736706	-0.063566
25	6	5.178196	5.942625	0.459964
26	6	-0.883437	3.891622	-0.020780
27	1	-1.480787	4.641202	0.490653
28	6	5.190672	4.832675	1.311264
29	1	5.830506	4.805629	2.186147
30	6	7.524269	-6.694105	-0.282599
31	6	3.924680	-4.061937	1.469615
32	1	3.263059	-3.840179	2.302539
33	6	4.317852	5.953167	-0.647905
34	1	4.323756	6.820706	-1.300180
35	6	4.869835	-5.068535	1.597175
36	1	4.959247	-5.644839	2.512765
37	6	3.489766	4.872308	-0.896636
38	1	2.832963	4.887730	-1.761680
39	6	6.837482	7.087378	1.718045
40	8	-6.597043	6.448282	0.600272
41	8	-5.988976	-6.962737	0.776924
42	7	-2.355729	-0.098883	-0.232000
43	6	-4.323488	-4.046361	-0.752617
44	1	-4.300360	-3.488778	-1.684880
45	6	1.443758	-2.519806	0.220661
46	6	-4.585108	0.500313	-0.227059
47	1	-5.465612	1.123079	-0.303266
48	6	-1.194580	-2.645745	0.222956
49	6	-5.725813	5.421153	0.444231
50	6	-3.126105	-1.200449	-0.088522
51	6	-4.530983	-0.853778	-0.105964
52	1	-5.354589	-1.547944	-0.012815
53	6	-5.634878	4.316597	1.297780
54	1	-6.272167	4.229978	2.170633
55	6	-2.835236	2.334324	-0.346316
56	6	-3.213909	0.973500	-0.291181
57	6	-4.700937	3.316358	1.035082
58	1	-4.622122	2.474054	1.715948
59	6	-3.840934	3.385155	-0.069433
60	6	-0.502125	-3.876350	0.351944
61	1	-1.038638	-4.821100	0.392663
62	6	-2.571166	-2.490786	0.148406
63	6	-3.480170	-3.659789	0.294825
64	6	-5.199117	-5.880062	0.552309
65	6	0.871915	-3.810785	0.350610
66	1	1.495951	-4.700205	0.390286
67	6	-5.177379	-5.142884	-0.635747
68	1	-5.807910	-5.414714	-1.474954
69	6	-7.492096	6.403726	1.693860
70	6	-3.938155	4.515036	-0.904719
71	1	-3.283016	4.591673	-1.767757
72	6	-4.360590	-5.506320	1.610755
73	1	-4.394343	-6.085547	2.528241
74	6	-4.866566	5.512187	-0.660781
75	1	-4.953237	6.374065	-1.314825
76	6	-3.516104	-4.414339	1.479527
77	1	-2.878564	-4.127626	2.311408
78	45	0.054358	-1.117431	-0.061963
79	17	0.083818	-1.771369	-2.337168
80	8	0.019579	-0.365269	2.854804
81	6	0.032327	-0.639324	1.739763
82	52	-0.072395	1.514497	-1.441009
83	1	8.138997	-7.511490	0.097644
84	1	6.993169	-7.032452	-1.181844
85	1	8.173863	-5.849357	-0.546948
86	1	7.355140	8.045377	1.652063
87	1	6.303761	7.032401	2.675561
88	1	7.574662	6.275685	1.669915
89	1	-6.959395	6.402110	2.653516
90	1	-8.099162	7.307409	1.623755
91	1	-8.147649	5.524841	1.644772
92	6	-6.848798	-7.383495	-0.261953
93	1	-7.383367	-8.253941	0.121648
94	1	-7.575367	-6.604742	-0.528577
95	1	-6.288708	-7.673333	-1.160558

Cartesian coordinates for **3_A**‡:

Center Number	Atomic Number	Coordinates (Angstroms) X	Y	Z
1	8	-6.576982	-6.527609	-0.168827
2	8	-6.390408	6.708506	0.167426
3	7	-2.204129	0.030144	0.001235

4	6	-4.431878	3.797347	-0.972106
5	1	-4.312451	3.155133	-1.840272
6	6	1.341634	2.774647	0.261405
7	6	-4.429636	-0.615075	-0.040681
8	1	-5.287541	-1.269840	-0.092047
9	6	-1.263709	2.811104	0.261621
10	6	-5.671155	-5.519075	-0.095315
11	6	-3.022448	1.137978	0.067971
12	6	-4.410574	0.737533	0.047386
13	1	-5.249636	1.416159	0.100241
14	6	-5.497885	-4.683491	1.012843
15	1	-6.095989	-4.809811	1.908316
16	6	-2.683315	-2.419134	-0.170436
17	6	-3.053316	-1.054284	-0.064159
18	6	-4.534157	-3.676412	0.974329
19	1	-4.394361	-3.040640	1.844194
20	6	-3.730521	-3.477289	-0.153303
21	6	-0.638294	4.071380	0.304257
22	1	-1.211188	4.995508	0.292356
23	6	-2.614050	2.492053	0.171796
24	6	-3.630945	3.579276	0.153959
25	6	-5.513242	5.674938	0.094438
26	6	0.751663	4.051930	0.304073
27	1	1.350080	4.959732	0.291955
28	6	-5.366949	4.831053	-1.011320
29	1	-5.964271	4.970914	-1.905300
30	6	-7.398630	-6.766699	0.955908
31	6	-3.915870	-4.329151	-1.255056
32	1	-3.309011	-4.188432	-2.145371
33	6	-4.716233	5.470206	1.228904
34	1	-4.847305	6.130370	2.080529
35	6	-4.871819	-5.333139	-1.231371
36	1	-5.023978	-5.986323	-2.084867
37	6	-3.789024	4.439663	1.253373
38	1	-3.183815	4.284800	2.142458
39	6	-7.200198	6.974396	-0.959898
40	8	6.575020	6.527339	0.165369
41	8	6.385079	-6.710196	-0.164865
42	7	2.203049	-0.031422	0.001027
43	6	4.427990	-3.801646	0.973416
44	1	4.306047	-3.162372	1.843391
45	6	-1.342647	-2.775903	-0.262755
46	6	4.428661	0.613135	0.045735
47	1	5.286756	1.267579	0.097903
48	6	1.262491	-2.812462	-0.262748
49	6	5.670288	5.517746	0.093320
50	6	3.021107	-1.139531	-0.064891
51	6	4.409298	-0.739472	-0.042375
52	1	5.248219	-1.418322	-0.094431
53	6	5.501766	4.676747	-1.011475
54	1	6.103044	4.799328	-1.905342
55	6	2.682483	2.417867	0.171143
56	6	3.052468	1.052789	0.067096
57	6	4.538080	3.669688	-0.972084
58	1	4.401839	3.029932	-1.839583
59	6	3.729476	3.476044	0.153006
60	6	0.637089	-4.072592	-0.311535
61	1	1.209923	-4.996807	-0.304764
62	6	2.612717	-2.493495	-0.170759
63	6	3.629708	-3.580716	-0.153720
64	6	5.510908	-5.675527	-0.097094
65	6	-0.752870	-4.053088	-0.311553
66	1	-1.351428	-4.960857	-0.304837
67	6	5.362240	-4.836447	1.011663
68	1	5.954242	-4.985593	1.907853
69	6	7.412650	6.750525	-0.950821
70	6	3.909799	4.333690	1.251155
71	1	3.299278	4.197209	2.139617
72	6	4.715421	-5.469759	-1.232450
73	1	4.848370	-6.127365	-2.085768
74	6	4.865828	5.337552	1.226685
75	1	5.014282	5.994915	2.077620
76	6	3.788584	-4.439209	-1.255023
77	1	3.184101	-4.282093	-2.144198
78	45	-0.019502	-1.329758	-0.188901
79	17	-0.022803	-1.594042	2.151862
80	8	-0.021485	-1.510128	-3.202113
81	6	-0.018897	-1.300121	-2.075902
82	1	-8.034998	-7.612303	0.690757
83	1	-6.806547	-7.026612	1.842912
84	1	-8.031186	-5.899982	1.187893
85	1	-7.805290	7.843864	-0.698747
86	1	-6.596962	7.209284	-1.846336
87	1	-7.864108	6.131075	-1.190649
88	1	6.834228	7.012988	-1.846092
89	1	8.056066	7.589788	-0.682557
90	1	8.037181	5.874970	-1.170882
91	6	7.221743	-6.926131	0.952890
92	1	7.805746	-7.818683	0.724618
93	1	7.905150	-6.081477	1.113510
94	1	6.640336	-7.095914	1.868853
95	45	0.018410	1.328164	0.190546
96	6	0.018341	1.302623	2.077321
97	8	0.021568	1.514787	3.203146
98	17	0.021303	1.590902	-2.150980

References

1. B. Sathyamoorthy, A. Axelrod, V. Farwell, S. M. Bennett, B. D. Calitree, J. B. Benedict, D. K. Sukumaran, M. R. Detty, *Organometallics* 2010, **29**, 15, 3431–3441.
2. D. P. Sweat, C. E. Stephens, *Synthesis* 2009, **19**, 3214–3218.
3. C.-H. Lee, H.-J. Kim, D.-W. Yoon, *Bull. Korean Chem. Soc.* 1999, **20,** 276–280.
4. CrysAlisPro 1.171.41.56a, Rigaku Oxford Diffraction (Poland): Wrocław, 2013.
5. SIR2011, M. C. Burla, R. Caliandro, M. Camalli, B. Carrozzini, G. L. Cascarano, C. Giacovazzo, M. Mallamo, A. Mazzone, G. Polidori and R. Spagna, *J. Appl. Cryst*., 2012, **45**, 357–361; SIR2014, M.C. Burla, R. Caliandro, , B. Carrozzini, G. L. Cascarano, C. Cuocci, C. Giacovazzo, M. Mallamo, A. Mazzone, and G. Polidori, *J. Appl. Cryst*., 2015, **48**, 306-309, SHELXL, G. M. Sheldrick, *Acta Cryst.* 2008, **A64**, 112-122.
6. M. J. Frisch, G. W. Trucks, H. B. Schlegel, G. E. Scuseria, M. A. Robb, J. R. Cheeseman, G. Scalmani, V. Barone, G. A. Petersson, H. Nakatsuji, X. Li, M. Caricato, A. V. Marenich, J. Bloino, B. G. Janesko, R. Gomperts, B. Mennucci, H. P. Hratchian, J. V. Ortiz, A. F. Izmaylov, J. L. Sonnenberg, D. Williams-Young, F. Ding, F. Lipparini, F. Egidi, J. Goings, B. Peng, A. Petrone, T. Henderson, D. Ranasinghe, V. G. Zakrzewski, J. Gao, N. Rega, G. Zheng, W. Liang, M. Hada, M. Ehara, K. Toyota, R. Fukuda, J. Hasegawa, M. Ishida, T. Nakajima, Y. Honda, O. Kitao, H. Nakai, T. Vreven, K. Throssell, J. A. Montgomery, Jr., J. E. Peralta, F. Ogliaro, M. J. Bearpark, J. J. Heyd, E. N. Brothers, K. N. Kudin, V. N. Staroverov, T. A. Keith, R. Kobayashi, J. Normand, K. Raghavachari, A. P. Rendell, J. C. Burant, S. S. Iyengar, J. Tomasi, M. Cossi, J. M. Millam, M. Klene, C. Adamo, R. Cammi, J. W. Ochterski, R. L. Martin, K. Morokuma, O. Farkas, J. B. Foresman, and D. J. Fox, *Gaussian, Inc.*, Wallingford CT, 2016**.**
7. E. Pacholska-Dudziak, G. Vetter, A. Góratowska, A. Białońska, L. Latos-Grażyński, *Chem. Eur. J.* 2020, **26,** 16011–16018.
8. S. J. Thompson, M. R. Brennan, S. Y. Lee, S. J. Thompson, S. J. Thompson, M. R. Brennan, G. Dong, *Chem. Soc. Rev.* 2018, **47**, 929–981.
9. A. Takenaka, Y. Sasada, T. Omura, H. Ogoshi, Z.-I. Yashida, *J. Chem. Soc. Chem. Commun.* 1973, 792–793.
10. A. Srinivasan, H. Furuta, A. Osuka, *Chem. Commun.* 2001, *1*, 1666–1667.
11. E. Pacholska-Dudziak, M. Szczepaniak, A. Książek, L. Latos-Grażyński, *Angew. Chem. Int. Ed.* 2013, **52**, 8898–8903.
12. A. J. Arce, A. Karam, Y. De Sanctis, R. Machado, M. V. Capparelli, J. Manzur, *Inorganica Chim. Acta* 1997, **254**, 119–130.
13. M. Hernández, G. Miralrio, A. Arévalo, S. Bernès, J. J. García, C. López, P. M. Maitlis, F. Del Rio, *Organometallics* 2001, **20**, 4061–4071.
